# Functional Characterization of VS-186B, a Novel HDAC Inhibitor with Anticancer Activity

**DOI:** 10.3390/ijms262311354

**Published:** 2025-11-24

**Authors:** Laura A. Sanchez-Michael, Vijayalakshmi Sudarshan, Allison Elias, Denisse A. Gutierrez, Jose A. Lopez-Saenz, Jaqueline Pena-Zacarias, Gabriela C. Torres, Armando Varela-Ramirez, Sujeet Kumar, Subhas S. Karki, Renato J. Aguilera

**Affiliations:** 1 Department of Biological Sciences, Border Biomedical Research Center, The University of Texas at El Paso, El Paso, TX 79968, USA; lasanchez17@miners.utep.edu (L.A.S.-M.); aelias10@miners.utep.edu (A.E.); dagutierrez18@miners.utep.edu (D.A.G.); jalopez62@miners.utep.edu (J.A.L.-S.); jpenazacar@miners.utep.edu (J.P.-Z.); torresg7@uthscsa.edu (G.C.T.); avarela2@utep.edu (A.V.-R.); 2 Department of Pharmaceutical Chemistry, KLE College of Pharmacy, Bengaluru 560010, Karnataka, India; vijayalakshmis.py.ph@msruas.ac.in; 3 KLE Academy of Higher Education and Research, KLE Deemed to be University, Belagavi 590010, Karnataka, India; 4 Department of Pharmaceutical Chemistry, Faculty of Pharmacy, M.S. Ramaiah University of Applied Sciences, Bengaluru 560054, Karnataka, India; 5 Department of Pharmaceutical Chemistry, NITTE College of Pharmaceutical Sciences, Nitte-Deemed to be University, Bengaluru 560064, Karnataka, India; klempharma@gmail.com

**Keywords:** cytotoxicity, apoptosis, HDAC inhibitor, anti-cancer, selectivity, curcumin, Vorinostat, leukemia, Jurkat

## Abstract

Histone acetylation and deacetylation are key regulators of gene expression and are frequently dysregulated in cancer, contributing to tumorigenesis and drug resistance. Overexpression of histone deacetylases (HDACs) in many cancer types leads to silencing of tumor suppressor genes and uncontrolled proliferation. Tumors often rely on epigenetic mechanisms to escape therapy and develop resistance. This study aimed to identify novel compounds that selectively target cancer cells while minimizing toxicity to non-cancerous cell lines. A series of novel HDAC inhibitors was evaluated using the Differential Nuclear Staining (DNS) assay, flow cytometry, and HDAC inhibition assays. These assays assessed cytotoxicity, selectivity, and mechanisms of cell death. Among seven compounds tested, VS-186B exhibited the highest cytotoxicity and Selective Cytotoxicity Index (SCI), particularly against the human Jurkat T-cell leukemia cell line. Flow cytometry experiments (Annexin V-FITC, ROS, JC-1, and Caspase-3/7 assays) revealed that VS-186B induced apoptosis. VS-186B was more cytotoxic than Curcumin and Vorinostat across most of the cell lines tested and was more specific to hematological cells. Connectivity Map (CMap) analysis showed strong similarity to genes affected by known HDAC inhibitors. Subsequently, HDAC enzymatic assays confirmed that VS-186B inhibits Class I and II HDACs in a dose-dependent manner. VS-186B exhibits promising anticancer potential as a selective HDAC inhibitor since it induces apoptosis in cancer cells without significant cytotoxicity to non-cancerous lines with a similar gene expression profile to known HDAC inhibitors. These findings support further development of VS-186B as an epigenetic treatment for leukemia/lymphoma.

## 1. Introduction

Cancer is defined as uncontrolled cell proliferation and is not only driven by genetic mutations but is also affected by epigenetic alterations, which influence gene expression without altering the DNA sequence [1]. When core histones become acetylated, the positive charge on lysine residues is neutralized, weakening interactions with the negatively charged DNA molecule [2]. While histone acetylation is typically associated with an open chromatin (euchromatin) state, allowing access to transcription factors and promoting gene expression, deacetylation is associated with closed chromatin (heterochromatin), leading to gene silencing [2,3].

HDACs are often overexpressed in various cancers, driving cancer cell proliferation and repressing tumor suppressor genes such as p53 and p21 [2,4]. HDAC inhibitors can potentially reactivate tumor suppressor genes, induce apoptosis, and inhibit tumor growth [5]. Several HDAC inhibitors, including SAHA (Vorinostat), Belinostat, Panobinostat, and Romidepsin, have been approved by the U.S Food and Drug Administration (FDA) for cancer treatment. In contrast, others are being evaluated in clinical trials [6]. Unfortunately, many existing HDAC inhibitors face significant limitations, including drug resistance, toxicity, and reduced efficacy in solid tumors [7].

Despite the global use of chemotherapy, the lack of selectivity of these treatments remains a significant limitation, contributing to widespread toxicity and the development of drug resistance [8]. Cancer cells develop resistance through specific mechanisms, including the activation of efflux pumps, mutations that alter drug binding sites, and the repair of treatment-related damage [9]. These mechanisms affect treatment outcomes, leading to relapses and poor prognosis [9,10]. These negative outcomes highlight the critical need for novel therapeutic drugs that selectively target cancer cells while leaving normal tissues unaffected. Specific targeted inhibitors, such as HDAC inhibitors, offer a more selective approach and are known to reduce toxicity while overcoming resistance mechanisms [7].

In this context, natural compounds and their analogues have garnered special interest due to their reduced toxicity and diverse range of biological functions. Notably, over fifty percent of the currently available anticancer medications either originate from natural sources or replicate the mechanisms of action found in natural substances [11]. Among these, curcumin has gained prominence due to its multi-targeted activity and relatively low toxicity profile. Curcumin, a naturally occurring polyphenolic substance, is a linear diarylheptanoid natural product that contains two oxygen-substituted aryl groups connected by a seven-carbon chain [12]. This phytochemical exhibits a wide range of biological activities, such as antiarthritis, antiatherosclerosis, antibacterial, antidiabetic, anti-fungal, antihypertensive, antihyperlipidemic, anti-inflammatory, antitumour, antiphlogistic, antipsoriatic, antithrombotic, and antihepatotoxic properties [13].

Curcumin’s capacity to inhibit stromal protection and avoid chemotherapy resistance makes it a promising treatment option for leukemia. It also has immunopotentiating properties and shields lymphocytes from genotoxic effects, which are known to cause secondary malignancies [14,15,16]. Curcumin’s poor oral bioavailability and weak pharmacokinetic profile limit its usefulness as a drug candidate [17,18,19], and to address these drawbacks, medicinal chemists have focused on the structural modification of curcumin, leading to the synthesis of its analogues—i.e., new chemical entities (NCEs) based on the curcumin scaffold. Modifications commonly target four key regions, such as the aromatic side chain, linker chain, diketo functionality, and active methylene group [20,21,22].

**Figure 1:**
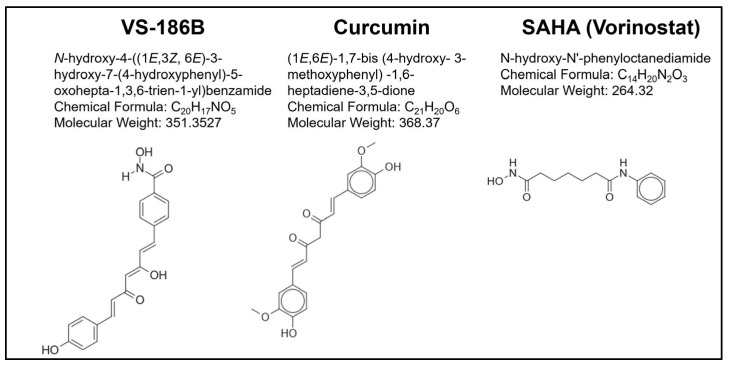
Chemical structures, International Union of Pure and Applied Chemistry (IUPAC) names, chemical formulas, and molecular weights of the novel HDAC inhibitor VS-186B, as well as Curcumin and the FDA-approved HDAC inhibitor Vorinostat. Curcumin and Vorinostat share structural similarities with VS-186B and exhibit HDAC inhibitory activity. Both were included for comparative purposes. Structures of other novel HDAC inhibitors are shown in Supplementary Figure S2. Structures were drawn using Marvin JS version 25.1.0 (ChemAxon).

Adding a 4-CONHOH group to one side of the curcumin linker and making changes to the other aromatic ring of curcumin was an approach taken in this study. This study further aims to address the above challenges by characterizing novel HDAC inhibitors to determine their selectivity, mechanisms of action, and potential as a more efficient treatment as depicted in Figure 1.

Our study evaluates the cytotoxic activity and selectivity of VS-186B across a diverse range of hematological and solid tumor cell lines. The mechanism of cell death induced by VS-186B was investigated by measuring apoptosis, oxidative stress, and mitochondrial dysfunction. Our data reveals VS-186B to exhibit higher cytotoxicity than FDA-approved Vorinostat in hematological cell lines, with low toxicity towards non-cancerous cells. Our research aimed to contribute to the development of more effective and less toxic therapies.

## 2. Results

### 2.1. Chemistry

**Figure 2:**
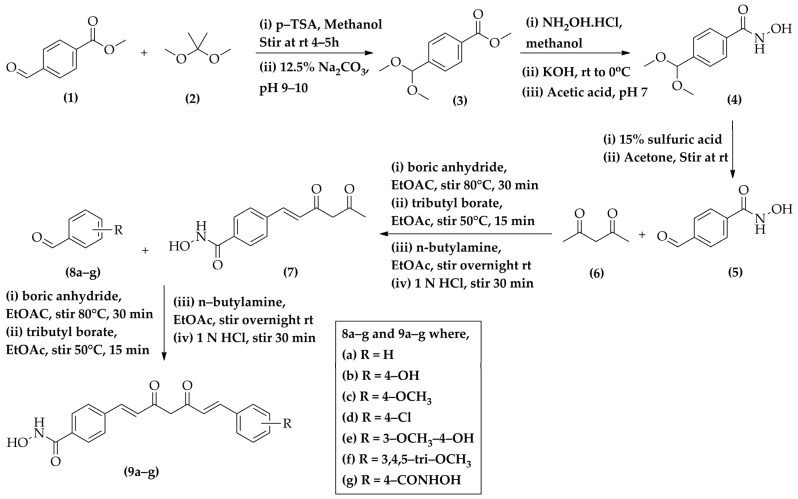
Synthesis of curcuminoids **9a**–**g**.

A set of seven potential HDAC inhibitors, **9a**–**g**, was synthesized according to Figure 2. A crucial intermediate **7** is created when 2.5 mM of 4-formyl-N-hydroxybenzamide (**5**) and 10 mM of 2,4-pentanedione (**6**) combine with boric anhydride, tributyl borate, and n-butylamine. Furthermore, in the presence of boric anhydride, tributyl borate, and n-butylamine, compound (**7**) reacts with various benzaldehydes **8a**–**g** to form compounds **9a**–**g**, with yields ranging from 15% to 25%.

All synthesized curcuminoids **9a**–**g** were characterized by their Fourier Transform Infra-Red (FTIR), proton/carbon nuclear magnetic resonance (^1^H/^13^C NMR), and HR-MS spectral data (Supplementary Tables S1 and S2 and Supplementary Figures S3–S31). The FTIR spectra of **9a**–**g** showed stretching absorption bands for C-H aromatic in the range of 3113–3010 cm^−1^, C-H aliphatic in 2967–2849 cm^−1^, C=O in 1708–1605 cm^−1^, and C=C aromatic in 1586–1498 cm^−1^. The analog **9b** exhibited characteristic stretching vibration in 1707 and 1605 cm^−1^ for C=O.

The ^1^H NMR spectra of **9a**–**g** showed significant signals for OH alcoholic protons between δ 11.32–11.28 ppm, NH protons between δ 9.13–9.08 ppm, ethylene protons between δ 7.09–6.86 ppm, and for enolic OH protons between δ 6.23–6.10 ppm. The aromatic protons were visible in the range of 7.86–7.41 ppm, and the methoxy protons of **9c**, **9e**, and **9f** were found to be between δ 3.84 and 3.67 ppm. For curcuminoid **9b**, prominent signals were observed at δ 11.28 ppm for alcoholic OH proton, at δ 10.08 ppm for aromatic OH proton, at δ 9.08 ppm for NH proton, between δ 7.82–7.53 ppm for aromatic protons, between δ 7.00–6.70 ppm for ethylene protons, and at δ 6.10 ppm for enolic OH proton.

The ^13^C NMR spectra of **9a**–**g**, exhibited peaks in the range of δ 186–183 ppm for C=O of carbonyl groups, peaks in the range of δ 164–163 ppm for C=O of amide groups, peaks in the range of 130–102 ppm for aromatic carbons and the peaks in the range of δ 56–55 ppm for methoxy carbons of **9c**, **9e** and **9f** on benzene ring. Ethylene carbon peaks of -CH=CH of the analogs were shown between δ 142–138 ppm. For curcuminoid **9b**, prominent signals were noted at δ 186.12 ppm for C=O of the carbonyl group, at 164.04 ppm for C=O of the amide group, between 142.08–138.82 ppm for -CH=CH of the ethylene carbons, and between δ 128.95 and 102.38 ppm for aromatic carbons.

The HR-MS spectrum of curcuminoids **9a**–**g** showed a molecular ion peak corresponding to their molecular weights; hence, the identity of all the compounds was confirmed. For curcuminoid **9b**, the mass was corroborated by HRMS with the molecular peak exhibited at 350.1026 (calc. 351.1107).

### 2.2. VS-186B Exhibits Potent Cytotoxicity and Selectivity Against Hematological and Solid Tumor Cell Lines

Seven potential HDAC inhibitors were tested across various cancer cell lines, and VS-186B was identified as the most potent compound based on its CC_50_ (concentration that kills 50% of the cells) values. Lower CC_50_ values indicate higher drug potency, while higher values reflect reduced cytotoxicity. Curcumin, a natural compound with anticancer properties [23] and Vorinostat, an FDA-approved HDAC inhibitor used in standard treatment [24] served as reference compounds. To assess the potency of VS-186B, we compared its CC_50_ values to those of Curcumin and Vorinostat across seven hematological cancer cell lines (Jurkat, CCRF-CEM, KMS II, HL-60, MM.1S, Ramos, and NALM6) at 48 or 72 h.

VS-186B was also evaluated across a range of solid tumor cell lines, including five breast cancer cell lines line (MDA-MB-468, MDA-MB-231, MDA-MB-231-LM2-4, T-47D, MCF-7), one liver cancer cell line (HEP G2), one colorectal cancer cell line (Caco-2), one pancreatic cancer cell line (PANC-1), and one lung cancer cell line (A549). In nearly all tested cell lines, VS-186B exhibited greater cytotoxicity than both Curcumin and Vorinostat, including both hematological and solid tumors (Table 1). These results suggest that lower doses of VS-186B may be effective in clinical settings, potentially reducing patient side effects.

**Figure 3:**
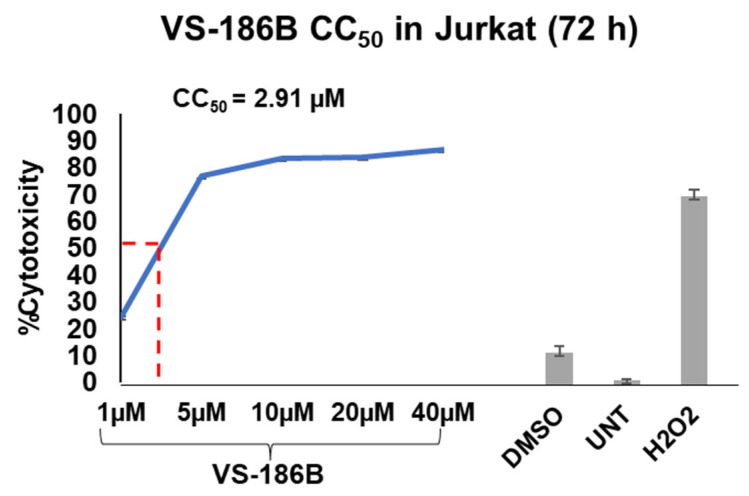
Dose–response curve of VS-186B: Cells were exposed for 72 h to VS-186B at various concentrations, and the DNS assay was used to determine a CC_50_ of 2.91 µM. Increasing concentrations as well as vehicle (DMSO), negative (Untreated), and positive (H_2_O_2_) controls are depicted on the x-axis. At the same time, the percentage of cytotoxicity (percentage of dead cells) is displayed on the y-axis. VS-186B exhibited the highest cytotoxicity in Jurkat cells at 72 h. The red dotted line indicates the CC_50_ value.

VS-186B exhibited higher potency in hematological cancer cell lines than solid tumor cell lines (Table 1). Solid tumors required a higher Vorinostat concentration to achieve comparable cytotoxicity compared to hematological cancer cell lines. At 48 h, VS-186B was the most cytotoxic to CEM, Ramos, and Jurkat cells. At 72 h, VS-186B displayed the most potency towards Jurkat with a CC_50_ of 2.91 µM (Figure 3) Ramos, HL-60, NALM6, and KMS-11 were only tested at 48 h due to their sensitivity when exposed for longer incubation times (Table 1). Therefore, the only hematological cell lines tested at 72 h were Jurkat, CEM, and MM.1S (Table 1).

Interestingly, VS-186B displayed similar CC_50s_ at 24 and 48 h of ~4 µM (Supplementary Table S3). However, at 72 h, there was a significant increase in cytotoxicity (Table 1). This suggests that VS-186B’s maximal cytotoxic effect requires more prolonged exposure and could be related to epigenetic mechanisms such as HDAC inhibition.

Our analysis revealed VS-186B to be very cytotoxic to multiple cancer cell lines, specifically hematological cell lines like Jurkat. Next, we aimed to determine whether VS-186B exhibits selectivity towards cancer cells. The Differential Nuclear Staining (DNS) Assay was performed on non-cancerous controls Hs27 and MCF 10A cell lines by exposing them to various concentrations for 72 h, and CC_50_ values were obtained (Table 2). The CC_50__s_ of each breast cancer cell line were divided by the CC_50_ value obtained for the MCF 10A non-cancerous breast epithelial cell line to obtain the Selective Cytotoxicity Index (SCI) of each cancer cell line. Similarly, the CC_50_ value of the Hs27 non-cancerous human foreskin fibroblast cell line was used to determine the Selective Cytotoxicity Index (SCI) values of all other cancer cell lines (see Table 2) [25].

The Jurkat T-Cell Leukemia cell line exhibited the highest SCI of 16.15 at 72 h (Table 2). This value indicates that it takes 16 times more of VS-186B to kill 50% of normal cells than it does to kill 50% of Jurkat cells. These findings suggest that VS-186B holds strong therapeutic promise for acute lymphoblastic leukemia (ALL), an aggressive cancer with limited treatment options.

### 2.3. VS-186B Induces Intrinsic Apoptotic Cell Death

**Figure 4:**
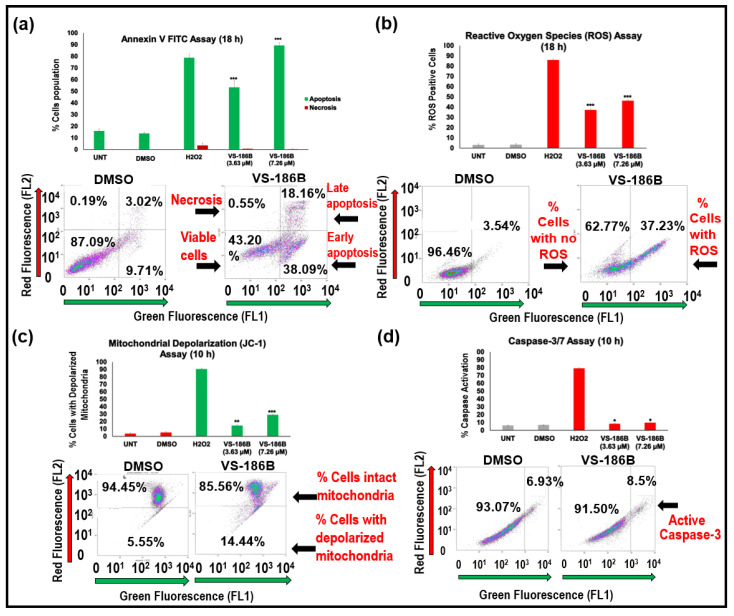
(**a**) Phosphatidylserine externalization was observed after 18 h of exposure to VS-186B, indicating apoptotic cell death. Cells were stained with Annexin V FITC and PI and analyzed through flow cytometry. The x-axis represents negative (untreated), vehicle (DMSO), and positive (H_2_O_2_) controls, as well as VS-186B at its 24 h CC_50_ (3.63 µM) and CC_50_ × 2 (7.26 µM). The y-axis dictates the percentage of cells undergoing apoptosis. The summation of both early and late apoptotic subpopulations determines the total percentage of apoptotic cells. Representative flow cytometry plots demonstrate viable cells, early apoptotic cells, late apoptotic cells, and necrotic cells after CC_50_ and DMSO treatment at 24 h. (**b**) After 18 h of exposure to VS-186B, there is significant ROS production. Flow cytometry plots indicate the percentage of cells induced by ROS after CC_50_ and DMSO treatment. Gray represents the negative and vehicle controls and red represents the positive control and treatments. (**c**) Jurkat cells were treated with VS-186B for 10 h, and mitochondrial membrane depolarization was assessed by JC-1 staining and flow cytometry analysis. Flow plots indicate percentage of cells with depolarized mitochondria after CC_50_ and DMSO treatment. (**d**) Treatment of VS-186B for 10 h resulted in caspase-3/7 activation, supporting the activation of intrinsic apoptotic cell death. Flow plots represent active caspase percentage comparing CC_50_ and DMSO. Gray represents the negative and vehicle controls and red represents the positive control and treatments. Data represents the mean ± standard deviation (SD) of three independent measurements. A two-tailed Student’s paired *t*-test was performed by comparing DMSO to VS-186B samples. * *p* < 0.05, ** *p* < 0.01, *** *p* < 0.001. Black arrows point to the cell subpopulations shown in each flow cytometry plot.

Our findings confirm that VS-186B induces apoptosis through the intrinsic pathway in Jurkat cells, as indicated by phosphatidylserine externalization, elevated reactive oxygen species (ROS) levels, mitochondrial depolarization, and caspase-3/7 activation (Figure 4). These results suggest that the compound not only initiates early apoptotic events, as shown by the Annexin V FITC assay (Figure 4a), but also leads to the activation of caspases and other downstream molecules, resulting in programmed cell death (Figure 4d).

The significant increase in ROS levels and the loss of mitochondrial membrane potential suggest the involvement of oxidative stress as a contributor to cell death (Figure 4b,c). Caspase-3/7 activity following VS-186B treatment supports its role in promoting cell death, demonstrating that the compound not only initiates apoptosis but also effectively drives cells through the final phase. Annexin V FITC and ROS Assay were performed at 18 h, while JC-1 and Caspase-3/7 were conducted at 10 h. Flow cytometry plots are shown in Supplementary Figure S32 for Annexin/PI, Supplementary Figure S33 for ROS, Supplementary Figure S34 for JC-1, Supplementary Figure S35 for Caspase-3/7, and Supplementary Figure S36 for Cell Cycle.

### 2.4. VS-186B Does Not Alter Cell Cycle Progression

**Figure 5:**
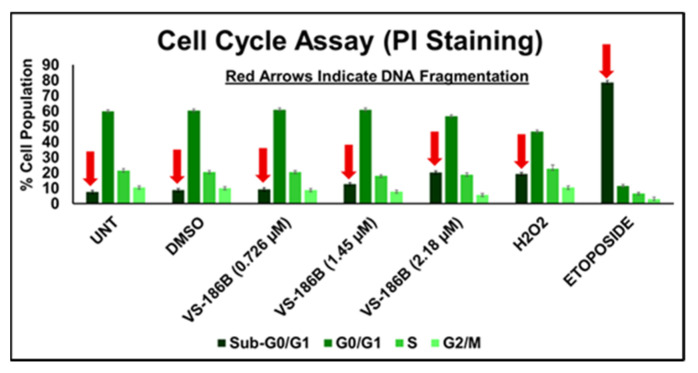
Cell Cycle Analysis of Jurkat Cells Treated with VS-186B: Jurkat cells were treated for 24 h with VS-186B at CC_10_ (0.726 µM), CC_20_ (1.45 µM), and CC_30_ (2.18 µM). No cell cycle phase arrests were observed at these concentrations; however, Sub-G_0_-G_1_ populations indicate DNA fragmentation. PI was used to measure DNA content by flow cytometry. Data represents the mean ± standard deviation (SD) of technical triplicates.

To further explore the mechanism of cell death induced by VS-186B, we conducted a cell cycle analysis using PI staining (Figure 5). While VS-186B treatment did not significantly alter the distribution of cells across G_0_/G_1_, S, or G_2_/M phases at the CC_10_ (0.726 µM), CC_20_ (1.45 µM), or CC_30_ (2.18 µM), there was an increase in the sub-G_0_/G_1_ population. This increase indicates DNA fragmentation, a hallmark of apoptosis [26]. These findings suggest that VS-186B does not induce cell cycle arrest at the tested concentrations but promotes apoptosis through DNA fragmentation.

### 2.5. VS-186B Displays Similar Transcriptional Profile to Known HDAC Inhibitors

A transcriptome and Connectivity Map (CMap) analyses were performed in the MDA-MB-231 breast cancer cell line to compare the effects of VS-186B treatment with the gene expression profiles of cells treated with known inhibitors. Treatment of VS-186B at 6 h was compared to a database of cancer cell lines also treated at 6 h with known perturbagens. As shown in Table 3, the gene signatures of cells treated with VS-186V revealed that the top 15 matches were to known HDAC inhibitors. Compound matches were filtered by perturbagen type (“Compound”) in the CLUE platform to retain only small-molecule drugs and other perturbagen classes (knockdown or overexpression of genes) were excluded. Some of these hits included Vorinostat, Trichostatin-A (TSA), and Panobinostat, all of which are major treatment options for cancer patients today. This data suggests that VS-186B likely acts as an HDAC inhibitor, as evidenced by eliciting a gene expression profile similar to a variety of HDAC inhibitors. Gene expression changes from VS-186B-treated MDA-MB-231 cells were compared against reference profiles from PC3, VCAP, A375, A549, JA1E, HCC515, HT29, MCF-7, and HEP G2 cell lines in the CMap database. Connectivity scores closer to 100 represent similar gene expression profiles to VS-186B treatment. The top-ranking compounds identified by this analysis are shown in Table 3.

### 2.6. VS-186B Inhibits HDAC Enzymatic Activity in a Dose-Dependent Manner

**Figure 6:**
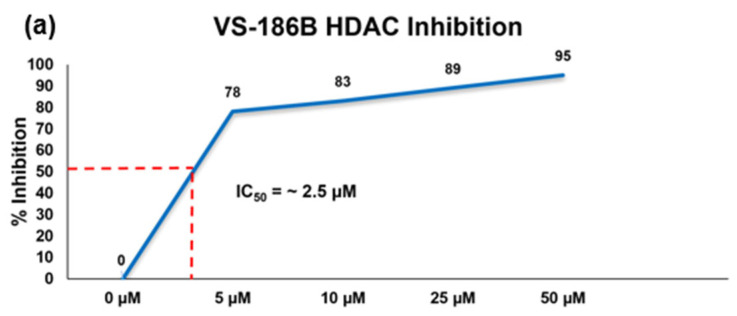

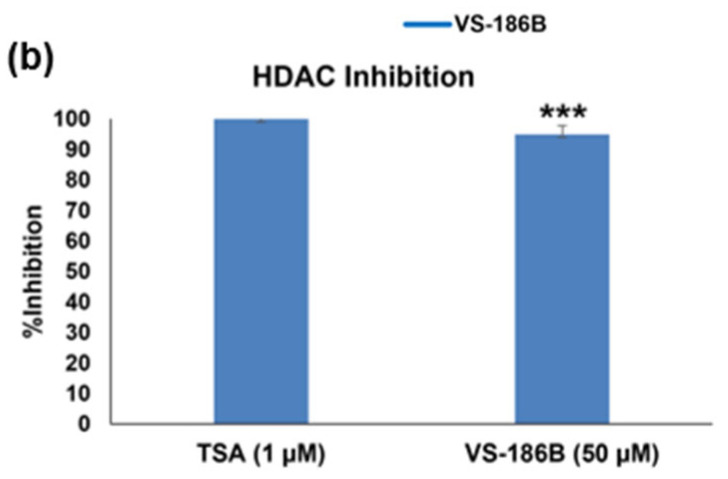
HDAC Inhibition by VS-186B (**a**) HDAC enzymatic assays confirmed that VS-186B inhibits HDAC activity in a dose-dependent manner. (**b**) At 50 µM, VS-186B achieved approximately 95% inhibition, indicating strong functional activity as an HDAC inhibitor. The IC_50_ is approximately 2.5 µM. Two-tailed *t*-tests were performed. Error bars = SD, *** *p* < 0.001. The red dotted line indicates the IC_50_ value.

To confirm VS-186B’s HDAC inhibitory activity, we performed a fluorometric HDAC activity assay. As shown in Figure 6a, VS-186B inhibited HDAC enzymatic activity in a dose-dependent manner, and an IC_50_ of approximately 2.5 µM was generated. At higher concentrations, VS-186B reached a maximal inhibition plateau of ~95% inhibition of HDAC activity, in comparison to the positive control Trichostatin A (TSA, 1 µM) (Figure 6b). These findings demonstrate that VS-186B functions as an HDAC inhibitor and support its role as an epigenetic regulator in cancer cells.

## 3. Discussion

The rationale behind the structural design and synthesis of curcumin analogues in this study is to address curcumin’s limitations while exploiting known reactivity patterns and pharmacophores to enhance anticancer efficacy. Curcumin, though bioactive, suffers from limited bioavailability due to its chemical and biological instability when faced with physiological conditions [18,20]. Therefore, modifications that replace either the 1,3-dicarbonyl chain or the aromatic side chain, or both, with chemically robust alternatives, can significantly improve pharmacokinetics and biological activity.

In the current study, the curcumin structure, which has a bis(4-hydroxy-3-methoxy) substitution on the benzene ring, was replaced on one side by a 4-CONHOH group [26] and by different groups like H, 4-OH, 4-OCH_3_, 4-Cl, 4-hydroxy-3-methoxy, 3,4,5-trimethoxy, and a4-CONHOH group on the other side. The aldehydes containing the above substitutions, when fused with 2,4-pentane dione linker through two steps, gave rise to the formation of unsymmetrical, highly conjugated analogs **9a**–**g**. All the novel HDAC inhibitors were evaluated for their cytotoxic activity. Among them, VS-186B demonstrated the highest cytotoxicity, and the study focused on comparing its effects to Curcumin and Vorinostat.

Our results suggest that VS-186B could be administered in lower doses in clinical settings, which may help minimize side effects often associated with higher-dose HDAC inhibitor treatments. Interestingly, VS-186B was more cytotoxic than both Curcumin and Vorinostat in five out of seven hematological cancer cell lines and eight out of nine solid tumor cell lines (Table 1). VS-186B also exhibited a selective cytotoxicity index (SCI) greater than 1 in 9 out of 12 cell lines, showing promising potential as an HDAC inhibitor with strong selectivity. A previous study using three curcumin derivatives, curcumin-like dienone, induced apoptosis in human triple-negative breast cancer MDA-MB-231 cells and exhibited CC_50__s_ of 0.72 to 0.91 µM and an SCI of 45 to 111 [25].

Of the hematological cancer cell lines tested, VS-186B displayed the lowest CC_50_ of 2.91 µM in the Jurkat cell line at 72 h (Table 1 and Figure 3). Jurkat cells also exhibited the highest selectivity for VS-186B, with an SCI value of 16.15 when compared to the non-cancerous Hs27 cell line (Table 2). Clinical studies have shown hematological cancers to be more responsive to single-agent HDAC inhibitor therapy when compared to solid tumors [2,27]. To this day, the exact reasons are not understood, but various factors can contribute to this difference. Blood-based cancers consist of cancer cells that rapidly proliferate and circulate in the bloodstream and bone marrow, or reside in lymphatic tissues, which makes them more accessible to drugs that are administered systemically [28].

Solid tumors contain cells growing at different rates, some actively dividing and some in the quiescent phase. Some solid tumor cells may grow more slowly due to limited nutrients, hypoxia, and stress signals [29]. Many solid tumors also contain dense stromal tissue, which can cause a physical barrier, making it hard for drugs to reach cancer cells [30] and can also block immune cells from getting into the tumor [31]. Dense stromal tissue can contribute to drug resistance [32] and often contains fibroblasts that secrete growth factors, further stimulating cancer cell growth [33]. Additionally, solid tumors can undergo epithelial-to-mesenchymal transition (EMT), an invasive process that contributes to drug resistance and reduced tumor selectivity [34]. Poor vascularization is also common in solid tumors, leading to hypoxia and reduced drug penetration and efficacy [9,28]. These anatomical and physiological barriers reduce drug distribution and penetration, limiting tumor selectivity in solid tumors compared to the more accessible environment of hematological cancers.

Our results show that VS-186B may have advantages over existing approved HDAC inhibitors. VS-186B also demonstrates potential in treating solid tumors, with the lowest CC_50__s_ in breast cancer and highest selectivity in liver, lung, and pancreatic cancer (Table 2). Future studies are needed to gain more insight into VS-186B’s effect on solid tumors.

Flow cytometry experiments, including Annexin V FITC, ROS, mitochondrial depolarization, and caspase-3/7 activation assays, revealed that VS-186B induces cell death primarily through the intrinsic apoptotic pathway (Figure 4), and cell cycle analysis (Figure 5) showed that treatment of VS-186B at its 24 h CC_10_, CC_20_, and CC_30_ did not significantly alter cell cycle distribution across G_0_/G_1_, S, and G_2_/M phases. However, VS-186B treatment resulted in DNA fragmentation (Figure 4), suggesting that apoptosis occurs independently of cell cycle arrest.

Connectivity map (CMap) analysis exhibited similar transcriptomic profiles between VS-186B and other known HDAC inhibitors, including Vorinostat and Trichostatin A (TSA), further supporting its HDAC inhibitory potential (Table 3 and Supplementary Figure S37). Table 3 summarizes the top drug hits showing similarity to VS-186B in the MDA-MB-231 cell line, while Supplementary Figure S37 presents a heatmap of compounds with comparable transcriptional signatures across multiple cell lines. Future studies, however, are needed to validate this in hematological cell lines. These studies will confirm whether these transcriptional responses are consistent across both solid and hematological cancers.

Subsequent HDAC enzymatic assays confirmed that VS-186B inhibits HDAC activity in a dose-dependent manner (Figure 6). VS-186B inhibited HDAC activity with a CC_50_ ~ 2.5 µM, consistent with its cytotoxicity in cancer cells. Although ~95% HDAC inhibition was observed at 50 µM, this high concentration primarily defines the assay plateau. Potency is interpreted from the low micromolar CC_50_, and future isoform-specific assays will further clarify its inhibitory profile.

BOILED-Egg Model analysis compared pharmacokinetic dynamics of VS-186B, Vorinostat, and Curcumin, and revealed favorable properties for drug-likeness and oral absorption (Supplementary Figure S38 and Supplementary Table S4). VS-186B, Vorinostat, and Curcumin are all predicted to have high gastrointestinal absorption (HIA), suggesting they can be passively absorbed by the human intestine, supporting good bioavailability. All three compounds remain outside the blood–brain barrier permeable zone (yellow region), reducing Central Nervous System (CNS) exposure. The red dots indicate that these compounds are not substrates of P-glycoprotein (P-gp), meaning they are not likely to be pumped out of cells by efflux transporters. Results showed drug-likeness and indicated they were good candidates for oral absorption. VS-186B produced a bioavailability score of 0.56, supporting its drug-likeness and potential as an orally administered drug. While these computational predictions provide valuable preliminary insight, experimental validation in vitro and in vivo will be required to confirm these properties and fully characterize the pharmacokinetic behavior of VS-186B.

All current experiments are conducted in vitro and may not fully reflect drug response in an in vivo model. Planned future studies will include mouse xenograft models to evaluate VS-186B and determine its in vivo activity, based on in vitro data. We also plan to investigate isoform-specific HDAC inhibition to better understand the selectivity of VS-186B. One potential limitation is the use of DMSO, which is the solvent for our compounds and can be cytotoxic at high concentrations. To address this issue, future studies will focus on identifying a solvent suitable for in vivo administration. Overall, our study highlights VS-186B as a promising novel HDAC inhibitor with potent cytotoxic activity and selectivity and favorable pharmacokinetic predictions, and an anticancer drug that can be studied further in preclinical development.

## 4. Materials and Methods

### 4.1. Chemicals and Instruments

Solvents and reagents were pretested for purity. Silica gel 60 GF_254_ plates from Merck (Darmstadt, Germany) were used to monitor the reaction progress by thin-layer chromatography (TLC). The melting point was measured using a DBK melting point apparatus (Mumbai, India). FTIR spectra were obtained using IR-grade KBr (Loba Chemie, Mumbai, India), and the diffuse reflectance technique was employed on the Jasco FTIR 460+ (Tokyo, Japan). NMR ^1^H/^13^C spectra were recorded between 400 and 500/100 MHz in DMSO-d_6_ and CDCl_3_ on Bruker (Ultraspec AMX 400, Karlsruhe, Germany) and JEOL RESONANCE (JNM-ECZ400S, Akishima City, Tokyo, Japan). All values of chemical shift (δ) are reported in ppm, with TMS as the reference. The HR-MS spectra were obtained at the Institution of Excellence, Vijnana Bhavan, Mysuru, using a Waters, (Xevo G2-XS QTof, Milford, CT, USA).

### 4.2. General Procedure for Synthesis of Methyl 4-(Dimethoxymethyl)benzoate (***3***)

2,2-dimethoxypropane (**2**) (0.158 mol, 19.35 mL) and a catalytic amount of p-toluenesulfonic acid (0.0018 mol, 0.34 g) were added to a solution of methyl-4-formyl benzoate (**1**) (0.0304 mol, 4.99 g) in absolute methanol (70 mL). The mixture was stirred at room temperature (rt) for 4–5 h, with continuous monitoring by TLC (15% ethyl acetate:hexane). To the reaction mixture, a 12.5% sodium carbonate solution was added until the pH reached 9–10, and the mixture was stirred for 30 min. It was then extracted three times using dichloromethane, and the organic extracts were dried over sodium sulphate, filtered, and concentrated under reduced pressure to obtain the product in a liquid form. Yield 91.5%, b.p.: 143–146 °C (lit. 144 °C)

### 4.3. General Procedure for the Synthesis of 4-(Dimethoxymethyl)-N-hydroxybenzamide (***4***)

Hydroxylamine hydrochloride (0.209 mol, 14.52 g) was mixed with 100 mL of methanol until a clear solution was obtained. Potassium hydroxide (0.313 mol, 17.56 g) was slowly added at rt (increasing to 42 °C) and subsequently cooled to 0 °C. After 1 h of cooling, the reaction mixture was filtered, and the filtrate was added to methyl 4-(dimethoxymethyl)benzoate (**3**) (0.026 mol, 5.46 g) at rt. The mixture was stirred at rt with continuous monitoring by TLC (80% ethyl acetate:hexane). Once the starting material was absent, the methanol was concentrated in a vacuum at 50 °C to obtain a crude product. To this crude product, around 30 mL of distilled water was added, and the pH was adjusted to 7 using acetic acid. The resulting solution was extracted three times using ethyl acetate, and the organic extracts were dried over sodium sulfate, filtered, and concentrated under reduced pressure to yield a light pink solid of 4-(dimethoxymethyl)-*N*-hydroxybenzamide (**4**). Yield 92.7%, m.p.: 76–80 °C (lit. 73–77 °C) [26].

### 4.4. General Procedure for the Synthesis of 4-Formyl-N-hydroxybenzamide (***5***)

A total of 80 mL of a 15% sulfuric acid solution in water (*w*/*v*) was slowly added to a solution of 4-(dimethoxymethyl)-N-hydroxybenzamide (**4**) (0.023 mol, 4.85 g) in acetone (80 mL). The temperature of the resulting mixture increased to 35 °C. The reaction mixture was stirred at rt with continuous monitoring by TLC (80% ethyl acetate:hexane). Once the starting material was absent, 250 mL of water was added, and then the mixture was extracted three times using ethyl acetate. The organic extracts were dried over sodium sulphate, filtered, and concentrated under reduced pressure at 10–20 °C to yield an off-white solid of 4-formyl-*N*-hydroxybenzamide (**5**). Yield 59.8%, m.p: 185–186 °C (lit. 200 °C) [26]), HR-MS *m*/*z*: [C_8_H_7_NO_3_]^−^, 164.0701 (calc. 165.0426). (Figure S1).

### 4.5. General Procedure for the Synthesis of 4-(3,5-Dioxohex-1-en-1-yl)-N-hydroxybenzamide (***7***)

A mixture of 2,4-pentanedione (**6**) (10 mmol, 1 mL) and boric anhydride (20 mmol, 1.39 g) was dissolved in ethyl acetate (15 mL). The mixture was stirred at 80 °C for 30 min, and a solution of 4-formyl-*N*-hydroxybenzamide (**5**) (2.5 mmol, 0.41 g) and tributyl borate (40 mmol, 10.78 mL) in ethyl acetate (15 mL) was added to the reaction solution. The solution was stirred for 15 min at 50 °C with dropwise addition of n-butylamine (5 mmol, 0.48 mL) in 2 mL of ethyl acetate. The reaction mixture was stirred overnight at room temperature, and 1 N HCl (20 mL) was added. The mixture was then stirred for an additional 30 min. The organic layers were separated, and the aqueous fraction was extracted with ethyl acetate (30 mL). The organic extract was washed with saturated sodium bicarbonate solution and brine, dried over anhydrous sodium sulphate, filtered, and evaporated to afford 4-(3,5-dioxohex-1-en-1-yl)-*N*-hydroxybenzamide (**7**) as a light-brown solid. Yield 68%, m.p.: >290 °C, HR-MS *m*/*z*: [C_13_H_13_NO_4_]^−^, 246.0762 (calc. 247.0845; Figure S2).

### 4.6. General Procedure for the Synthesis of N-Hydroxy-4-((1E,6E)-7-(substitutedphenyl)-3,5-dioxohepta-1,6-dien-1-yl)benzamide (***9a***–***g***)

A mixture of 4-(3,5-dioxohex-1-en-1-yl)-*N*-hydroxybenzamide (**7**) (10 mmol, 2.47 g) and boric anhydride (20 mmol, 1.39 g) was dissolved in ethyl acetate (15 mL). The mixture was stirred at 80 °C for 30 min, and a solution of aldehydes **8a**–**g** (2.5 mmol) and tributyl borate (40 mmol, 10.78 mL) in ethyl acetate (15 mL) was added to the reaction. The solution was stirred for 15 min at 50 °C with a dropwise addition of n-butylamine (5 mmol, 0.48 mL) in 2 mL of ethyl acetate. The reaction mixture was stirred overnight at room temperature, and 1 N HCl (10 mL) was added, followed by stirring for an additional 30 min. The organic layers were separated, and the aqueous fraction was extracted with ethyl acetate (30 mL). The organic extract was washed with saturated sodium bicarbonate solution and brine, dried over anhydrous sodium sulphate, filtered, and evaporated to afford the resulting compounds **9a**–**g**, which were recrystallised from DMF-EtOH.

A summary of the chemical characterization (IR, NMR, HRMS) is shown in Table 4.

#### 4.6.1. N-Hydroxy-4-((1E,3Z,6E)-3-hydroxy-5-oxo-7-phenylhepta-1,3,6-trien-1-yl)benzamide (9a/VS-186C)

Light brown-colored, yield 20%, m.p. 216–218 °C.

#### 4.6.2. N-Hydroxy-4-((1E,3Z,6E)-3-hydroxy-7-(4-hydroxyphenyl)-5-oxohepta-1,3,6-trien-1-yl)benzamide (9b/VS-186B)

Dark brown-colored, yield 25%, m.p. 186–188 °C.

#### 4.6.3. N-Hydroxy-4-((1E,6E)-7-(4-methoxyphenyl)-3,5-dioxohepta-1,6-dien-1-yl)benzamide (9c/VS-183A)

Golden yellow-colored, yield 15%, m.p. 196–198 °C.

#### 4.6.4. 4-((1E,6E)-7-(4-Chlorophenyl)-3,5-dioxohepta-1,6-dien-1-yl)-N-hydroxybenzamide (9d/VS-183D)

Dark yellow-colored, yield 17%, m.p. 204–206 °C.

#### 4.6.5. N-Hydroxy-4-((1E,3Z,6E)-3-hydroxy-7-(4-hydroxy-3-methoxyphenyl)-5-oxohepta-1,3,6-trien-1-yl)benzamide (9e/VS-186A)

Wine red-colored, yield 18%, m.p. 232–234 °C.

#### 4.6.6. N-Hydroxy-4-((1E,3Z,6E)-3-hydroxy-5-oxo-7-(3,4,5-trimethoxyphenyl)hepta-1,3,6-trien-1-yl)benzamide (9f/VS-186E)

Rust brown-colored, yield 20%, m.p. 224–226 °C.

#### 4.6.7. 4,4′-((1E,3Z,6E)-3-Hydroxy-5-oxohepta-1,3,6-triene-1,7-diyl)bis(N-hydroxybenzamide) (9g/VS-169B)

Light brown-colored, yield 18%, m.p. 240–242 °C.

### 4.7. Cell Culture

All media used for cell growth were supplemented with 100 U/mL penicillin and 100 µg/mL streptomycin (Corning, Christiansburg, VA, USA). Hematological cancer cell lines (CCRF-CEM, Jurkat Clone E6-1, MM.1S, KMS-11, NALM6, and Ramos) were cultured in Roswell Park Memorial Institute (RPMI-1640) medium (Cytiva-HyClone, Logan, UT, USA). 10% fetal bovine serum (FBS) (Cytiva-HyClone, UT, USA) was added to the medium for CCRF-CEM, Jurkat, MM.1S, NALM6, and Ramos. KMS-11 was cultured in RPMI-1640 medium supplemented with 20% FBS. CCRF-CEM, Jurkat, KMS-11, NALM6, and Ramos are non-adherent hematologic cell lines maintained in suspension, while MM.1S is semi-adherent and requires gentle scraping for media changes and passaging. Prior to experimental treatments, cell viability for all non-adherent lines was assessed by PI staining and flow cytometry. 400 µL of cell suspension was distributed into two flow cytometry tubes (unstained and PI-stained) and 5 µL of Propidium Iodide (PI; MP Biomedicals, Solon, OH, USA) at a final concentration of 5 µg/mL was added to each tube, and samples were analyzed by flow cytometry. Only cultures with ≥95% viable cells were used for subsequent assays. All cell lines were used within passages 2–10 after thawing.

Adherent breast cancer cell lines (MDA-MB-468, MDA-MB-231, MDA-MB-231-LM2-4, and MCF-7), as well as the liver cancer cell line HEP G2, pancreatic cancer cell line PANC-1, and the non-cancerous human fibroblast cell line Hs27 were cultured in Dulbecco’s Modified Eagle’s Medium (DMEM, Corning, New York, NY, USA) supplemented with 10% FBS. T-47D breast cancer cells were maintained in RPMI-1640 medium (Cytiva-HyClone, Logan, UT, USA), supplemented with 10% FBS. The non-tumorigenic epithelial breast cell line MCF 10A was maintained in DMEM/F12 medium, supplemented with 10% FBS, 10 µg/mL insulin, 20 ng/mL epidermal growth factor (EGF), and 0.5 µg/mL hydrocortisone. The adherent colorectal cancer cell line Caco-2 was cultured in Eagle’s Minimum Essential Medium (EMEM), supplemented with 20% FBS. The A549 non-small cell lung cancer cell line was maintained in F-12K Medium with 10% FBS. All cells were incubated at 37 °C in a humidified incubator with 5% CO_2_.

### 4.8. Differential Nuclear Staining Assay

The Differential Nuclear Staining (DNS) assay was performed to evaluate cytotoxicity through live-cell imaging using the two fluorescent DNA intercalators Hoechst 33342 (Invitrogen, Waltham, MA, USA) and PI at a final concentration of 5 µg/mL for both dyes [35]. The DNS assay is suitable and robust for high-throughput screening initiatives with a Z’-factor of 0.86 [35]. Hoechst stains the nuclei of both live and dead cells, while PI selectively stains the nuclei of dead cells with compromised membranes. Cells were cultured in T-75 flasks, counted using a hemocytometer, and seeded into 96-well plates at a density of 10,000 cells per well in 100 µL of medium. For VS-186B, 1 mg was weighed out (2.85 × 10^−6^ mol, MW = 351.35 g/mol) and dissolved in 285 µL of DMSO to prepare a 10 mM stock solution, from which subsequent dilutions were prepared. Curcumin (1 mg; 2.71 × 10^−6^ mol, MW = 368.37 g/mol) was dissolved in 271 µL of DMSO to prepare a 10 mM stock solution. Vorinostat (1 mg; 3.78 × 10^−6^ mol, MW = 264.32 g/mol) was dissolved in 378 µL of DMSO to prepare a 10 mM stock solution. Molecular weights for the fullVS compound series are shown in Table S2.

Treatments were performed in triplicate using a range of compound concentrations (1–40 µM) and incubated for 24, 48, or 72 h. Controls included 1 mM hydrogen peroxide (H_2_O_2_) as a positive control, 1% *v*/*v* DMSO as a vehicle control, and untreated cells as a negative control. Two hours prior to the live-cell imaging, a final concentration of 5 µg/mL Hoechst 33342 and PI were added to each well. Fluorescent images were acquired using the ImageXpress Pico system (Molecular Devices, San Jose, CA, USA) with a 4× objective and a 2 × 2montage covering 100% of the well area per fluorescence channel. Image analysis and quantification of live and dead cells were performed using Cell Reporter Xpress Software version 2.9.4.1690. CC_50_ values were calculated using linear interpolation (https://www.johndcook.com/interpolator.html, accessed on 18 November 2025).

### 4.9. Annexin V FITC Assay

The Annexin V-FITC apoptosis assay was performed to evaluate the mechanism of VS-186B–induced cell death through the detection of phosphatidylserine externalization, an early marker of apoptosis. Jurkat cells were seeded into 24-well plates at a density of 100,000 cells/mL in RPMI-1640 culture medium and incubated overnight at 37 °C in a 5% CO_2_ atmosphere [36]. Treatments were applied in triplicate and included VS-186B at its 24 h CC_50_ concentration (3.63 µM), 1 mM H_2_O_2_ as a positive control, 1% *v*/*v* DMSO as a vehicle control, and untreated cells as a negative control. After 18 h of incubation, cells were transferred to flow cytometry tubes and centrifuged at 1200 rpm for 5 min. The supernatant was discarded, and cells were washed once with 500 µL of ice-cold PBS. Cells were stained using Annexin V-FITC and PI (Beckman Coulter, Miami, FL, USA), in 1× binding buffer (diluted from 10× stock provided in the kit), which contains calcium to support Annexin V binding, and the Annexin V-FITC Apoptosis Kit was used (Beckman Coulter, Brea, CA, USA). Samples were incubated on ice in the dark for 30 min, and 400 µL of 1× binding buffer was added to each tube, followed by immediate analysis via flow cytometry (Cytomics FC 500, Beckman Coulter). Annexin V-FITC and PI fluorescence were measured in FL1 and FL2 channels to detect live, early/late apoptotic, and necrotic cells. Early and late apoptotic populations were combined and compared against DMSO control. A total of 10,000 events per sample were collected, and data were analyzed using Kaluza 1.3 software.

### 4.10. Reactive Oxygen Species (ROS) Assay

Intracellular reactive oxygen species (ROS) levels were measured in VS-186B–treated Jurkat cells using the fluorescent probe 6-carboxy-2′,7′-dichlorodihydrofluorescein diacetate (DCFDA; Invitrogen, C400). Jurkat cells were seeded into 24-well plates at a density of 100,000 cells/mL in RPMI-1640 medium and treated in triplicate. After 18 h of treatment, cells were transferred to flow cytometry tubes and centrifuged at 1200 rpm for 5 min. The supernatant was discarded, and 1 mL of 1× PBS containing 10 µM DCFDA was added to the cell pellets. Samples were incubated at 37 °C in 5% CO_2_for 1 h. Following incubation, cells were centrifuged again at 1200 rpm for 5 min, the supernatant was removed, and 500 µL of pre-warmed 1× PBS was added. Cells were incubated for an additional 20 min at 37 °C, followed by immediate analysis using the Gallios Flow Cytometer (Beckman Coulter). The FL1 detector (green fluorescence, ~530 nm) was used to quantify intracellular ROS accumulation. Ten thousand events per sample were collected, and data were analyzed using Kaluza 1.3 software.

### 4.11. JC-1 Mitochondrial Depolarization Assay

Mitochondrial membrane depolarization following treatment with VS-186B and control compounds was assessed using the JC-1 assay (Molecular Probes, Carlsbad, CA, USA, M34152). Jurkat cells were seeded into 24-well plates at a density of 100,000 cells/well in 1 mL of RPMI-1640 medium and treated in triplicate. Plates were incubated for 10 h at 37 °C in a 5% CO_2_ atmosphere. After incubation, cells were transferred to flow cytometry tubes and centrifuged at 1200 rpm for 5 min. The supernatant was discarded, and cells were resuspended in 500 µL of 1× PBS containing 2 µM JC-1 dye. Samples were incubated at 37 °C for 30 min, then washed once with 2 mL of pre-warmed PBS. Samples were then resuspended with 300 µL of PBS and were analyzed immediately by flow cytometry (Beckman Coulter), with 10,000 events per sample collected. The FL2 detector was used to measure polarized mitochondria, and the FL1/FITC detector was used to measure depolarized mitochondria. Data was analyzed using Kaluza 1.3 software (Beckman Coulter).

### 4.12. Caspase-3/7 Assay

The activation of caspase-3/7 was measured using the NucView 488 Caspase-3 Substrate Assay Kit (Biotium, Hayward, CA, USA). Jurkat cells were plated in 24-well plates at a density of 100,000 cells/well and treated with VS-186B at its 24 h CC_50_ concentration for 10 h. The same positive control (1 mM H_2_O_2_), vehicle control (1% *v*/*v* DMSO), and untreated control were included as described in previous sections. Following incubation, cells were transferred to flow cytometry tubes and centrifuged at 1200 rpm for 5 min. The supernatant was discarded, and 500 µL of PBS containing NucView 488 substrate at a final concentration of 5 µM was added to each pellet. Samples were incubated in the dark at 37 °C in a 5% CO_2_ atmosphere for 30 min. After incubation, 300 µL of PBS was added to each tube, and samples were immediately analyzed using flow cytometry (Beckman Coulter), with 10,000 events per sample collected. A green, fluorescent signal indicated caspase-3 activation. Data was analyzed using Kaluza 1.3 software.

### 4.13. Cell Cycle Analysis

Cell cycle distribution was analyzed using PI staining to evaluate whether VS-186B treatment altered cell cycle progression. The Cell Cycle and Apoptosis Analysis Kit (PI Staining) were used (MCE, HY-K1071). Jurkat cells were seeded into 24-well plates at a density of 100,000 cells/well in 1 mL of RPMI-1640 medium. Cells were treated with VS-186B at CC_10_ (0.726 µM), CC_20_(1.45 µM), and CC_30_ (2.18 µM) for 72 h. Positive (H_2_O_2_), vehicle (DMSO), and untreated controls were included as described in previous sections. Cells were also treated with a high concentration of etoposide (Fisher Scientific, MA, USA; Cat# 34120525MG) for 24 h to serve as a positive control for DNA damage-induced apoptosis. After treatment, cells were fixed in 70% ethanol at −20 °C overnight, then washed with PBS and incubated with RNase A and 50 µg/mL propidium iodide for 30 min at 37 °C. DNA content was analyzed by flow cytometry to determine G_0_/G_1_, S, and G_2_/M phase distribution. One hundred thousand events per sample were collected and analyzed using Kaluza 1.3 software (Beckman Coulter).

### 4.14. Transcriptomic Analysis

1,000,000 MDA-MB-231 breast cancer cells were seeded into 5 mL of DMEM supplemented with 10% FBS in T-25 flasks and incubated overnight at 37 °C in a 5% CO_2_ atmosphere. The following day, treatments were applied in triplicate using VS-186B at 2× the 24 h CC_50_ concentration (5.82 µM). After a 6 h incubation, the media was collected into 15 mL sterile tubes, and cells were detached by adding 1 mL of 0.25% trypsin to each flask.

Following detachment, 1 mL of DMEM with 10% FBS was added to neutralize the trypsin, and the cell suspensions remaining were transferred to 15 mL tubes. Samples were centrifuged at 1200 rpm for 5 min, washed with 1 mL of pre-warmed PBS, and transferred to microcentrifuge tubes. Total RNA was extracted using the RNeasy Mini Kit (Qiagen, Germantown, MD, USA) according to the manual. RNA purity and concentration were assessed using a NanoDrop spectrophotometer (Thermo Fisher Scientific, Waltham, MA, USA), with A260/280 ratios between 1.8 and 2.1. RNA integrity numbers (RINs) were determined using an Agilent TapeStation, Santa Clara, CA, USA, with all samples yielding a RIN of 10.

### 4.15. Connectivity Map (CMap) Analysis

Differentially expressed genes from the transcriptome analyses were used to query the Connectivity Map (CMap) via the NIH LINCS database (https://lincsproject.org/) to identify small molecules with similar or contrasting gene expression signatures. The top 150 differentially expressed genes were uploaded to the CLUE platform for CMap analysis, and the results were ranked by connectivity scores. The database identified gene expression profiles most similar to those following treatment with VS-186B.

### 4.16. HDAC Inhibition Assay

Histone deacetylase (HDAC) activity was evaluated using the HDAC Fluorometric Drug Discovery Kit (Enzo Life Sciences, Farmingdale, NY, USA, BML-AK500). VS-186B was tested at various concentrations (0, 5, 10, 25, and 50 µM) to assess its inhibitory effect on HDAC enzymatic activity. Trichostatin A (TSA, 1 µM) was used as a positive control. Compounds were incubated with the fluorogenic HDAC substrate and HDAC enzyme in a black 96-well plate at 37 °C for 30 min. After incubation, the developer solution was added to release the fluorescent signal. Fluorescence was measured using a Fluoroskan Ascent plate reader (Thermo Fisher, Waltham, MA, USA) at an excitation wavelength of 360 nm and emission at 460 nm. Lower fluorescence compared to the vehicle control indicated that VS-186B significantly inhibited HDAC activity. All reactions were performed in triplicate.

### 4.17. Selective Cytotoxic Index (SCI) Calculation

Selectivity was calculated by dividing the CC_50_ values of non-cancerous cell lines MCF 10A and Hs27 by the CC_50_ values of cancerous cell lines. SCI values greater than 1 indicate preferential selectivity towards cancerous cells.

### 4.18. Statistical Analysis

*p*-values were calculated using Student’s *t*-test, and an average of triplicate values, along with standard deviation (SD), was calculated. * *p* < 0.05, ** *p* < 0.01, and *** *p* < 0.001.

## Figures and Tables

**Table 1:** CC_50_ values (µM) of VS-186B, Curcumin, and Vorinostat across various cancer cell lines.

CC_50_ Values in Hematological Cancer Cell Lines at 48 h (µM) *				
Cell Line	Cell Type	VS-186B	Curcumin	Vorinostat
CEM	T-Lymphoblast	3.18 ± 0.25	16.80 ± 2.88	7.55 ± 3.13
Ramos	B-Lymphocyte	4.17 ± 1.35	9.09 ± 1.48	5.16 ± 1.78
Jurkat	T-Lymphocyte	4.86 ± 0.15	28.96 ± 2.72	16.82 ± 1.66
HL-60	Leukemia	4.91 ± 0.22	13.53 ± 1.24	11.36 ± 1.46
NALM6	Precursor B-Cell Leukemia	5.00 ± 0.70	7.12 ± 0.40	25.95 ± 2.60
KMS-11	Multiple Myeloma	7.31 ± 1.03	6.72 ± 2.79	16.82 ± 1.66
MM.1S	Multiple Myeloma	8.52 ± 0.47	6.59 ± 0.29	14.26 ± 1.07
**CC_50_ Values in Hematological Cancer Cell Lines at 72 h (µM)**				
**Cell Line**	**Cell Type**	**VS-186B**	**Curcumin**	**Vorinostat**
Jurkat	T-Lymphocyte	2.91 ± 0.50	19.01 ± 1.39	9.32 ± 0.31
CEM	T-Lymphoblast	4.55 ± 0.73	20.63 ± 1.63	7.92 ± 2.06
MM.1S	Multiple Myeloma	4.71 ± 0.11	5.65 ± 0.35	12.29 ± 1.27
**CC_50_ Values in Adherent Cancer Cell Lines at 72 h (µM)**				
**Cell Line**	**Cell Type**	**VS-186B**	**Curcumin**	**Vorinostat**
MCF-7	ER+ Breast Cancer	3.18 ± 0.25	16.80 ± 2.88	7.55 ± 3.13
T-47D	ER+ Breast Cancer	5.00 ± 0.70	7.12 ± 0.40	25.95 ± 2.6
MDA-MB-231-LM2-4	TNBC	4.17 ± 1.35	9.09 ± 1.48	5.16 ±1.78
MDA-MB-231	TNBC	6.99 ± 0.15	21.29 ± 0.90	22.85 ± 0.27
MDA-MB-468	TNBC	8.53 ± 0.58	24.77 ± 0.52	24.21 ± 0.40
HEP G2	Liver Cancer	4.86 ± 0.15	28.96 ± 2.72	16.82 ± 1.66
A549	Lung Cancer	7.29 ± 0.44	26.02 ± 1.61	8.58 ± 0.15
PANC-1	Pancreatic Cancer	8.85 ± 0.90	20.25 ± 3.21	5.22 ± 0.50
Caco-2	Colorectal Cancer	22.19 ± 2.07	24.14 ± 1.42	5.09 ± 0.69

* CC_50_ values in µM with ±standard deviation.

**Table 2:** CC_50_ and Selective Cytotoxic Index (SCI) values for VS-186B in various cancer cell lines.

CC_50_ Values of VS-186B in Non-Cancerous Cells at 72 h		
Cell Line	Cell Type	VS-186B (µM) *
Hs27	Foreskin fibroblast	46.82 ± 1.26
MCF 10A	Breast epithelial	4.37 ± 0.73
**SCI of VS-186B in Cancer Cells at 72 h**		
**Cell Line**	**Cell Type**	VS-186B (µM) *
CEM	T-Lymphoblast	10.29
Jurkat	T-Lymphocyte	16.08
MM.1S	Multiple Myeloma	9.94
MCF-7	Breast Cancer (ER+)	1.37
T-47D	Breast Cancer (ER+)	0.87
MDA-MB-231-LM2-4	Triple Negative Breast Cancer (TNBC)	1.04
MDA-MB-231	TNBC	0.62
MDA-MB-468	TNBC	0.51
HEP G2	Liver Cancer	9.63
A549	Lung Cancer	6.42
PANC-1	Pancreatic Cancer	5.29
Caco-2	Colorectal Cancer	2.10

* CC_50_ values in µM with ±standard deviation.

**Table 3:** VS-186B induces a gene expression profile similar to FDA-approved HDAC inhibitors identified by Connectivity Map (CMap) analysis in the MDA-MB-231 cancer cell line.

Rank	Score	Name	Description
1	99.15	THM-I-94	HDAC inhibitor
2	99.08	WT-171	HDAC inhibitor
3	99.08	ISOX	HDAC inhibitor
4	99.01	Trichostatin-A	HDAC inhibitor
5	99.01	Pyroxamide	HDAC inhibitor
6	99.01	Droxinostat	HDAC inhibitor
7	99.01	Belinostat	HDAC inhibitor
8	99.00	Scriptaid	HDAC inhibitor
9	98.98	Givinostat	HDAC inhibitor
10	98.98	NCH-51	HDAC inhibitor
11	98.98	Vorinostat	HDAC inhibitor
12	98.94	HC-toxin	HDAC inhibitor
13	98.94	APHA-compound-8	HDAC inhibitor
14	98.87	Dacinostat	HDAC inhibitor
15	98.66	Panobinostat	HDAC inhibitor
16	98.66	BI-2536	PLK inhibitor
17	98.52	Apicidin	HDAC inhibitor
18	98.33	NVP-AUY922	HSP inhibitor
19	98.24	Piperlongumine	Glutathione transferase inhibitor
20	98.10	HSP90-inhibitor	HSP inhibitor

**Table 4:** IR, NMR and HRMS data of *N*-hydroxy-4-(1*E*,6*E*)-7-(substituted phenyl)-3,5-dioxohepta- 1,6-dien-1-yl)benzamide (**9a**–**g**).

Code	IR (cm^−1^)	^1^H-NMR (δ ppm)	^13^C-NMR (δ ppm)	HRMS Calc. Mass (Obs. Mass) -MS (ESI) *m*/*z*
**9a** **(VS186C)**	3289, 3113, 1708, 1643, 1428, 1189, 1165	6.19 (s, 1H, enolic OH), 6.94–7.04 (m, 2H, ethylene), 7.41–7.44 (m, 3H, Ar, *J* = 12), 7.62–7.65 (m, 2H, Ar), 7.71–7.79 (m, 7H, Ar), 9.08 (s, 1H, NH), 11.28 (s, 1H, OH)	184.58, 182.88, 164.03, 141.31, 139.66, 129.57, 129.54, 128.99, 128.82, 128.00, 126.30, 124.92, 102.71	335.1158 (334.1088)
**9b (VS186B)**	3109, 2850, 1707, 1605, 1522, 1346, 1197, 1105	6.10 (s, 1H, enolic OH), 6.70–7.00 (m, 4H, Ar), 7.53–7.63 (m, 4H, Ar), 7.71–7.82 (m, 5H, Ar), 9.08 (s, 1H, NH), 10.08 (s, 1H, Ar-OH), 11.28 (s, 1H, OH)	191.52, 186.12, 181.06, 164.04, 163.84, 160.59, 142.08, 138.82, 137.92, 134.20, 132.64, 131.15, 128.95, 128.68, 128.55, 127.97, 126.26, 126.18, 121.41, 116.47, 116.36, 102.38	351.1107 (350.1026)
**9c (VS183A)**	3280, 3028, 2914, 1694, 1624, 1498, 1281, 1183, 1142, 1114	3.79 (s, 3H, OCH_3_), 6.14 (s, 1H, enolic OH), 6.83 (d, 1H, *J* = 12), 6.98–7.02 (m, 3H, Ar), 7.59–7.64 (m, 2H, Ar), 7.70 (d, 2H, Ar, *J* = 8), 7.74–7.79 (m, 4H, Ar), 7.86 (s, 1H, Ar), 9.08 (s, 1H, NH), 11.29 (s, 1H, OH)	185.78, 181.69, 163.93, 161.60, 141.69, 139.23, 137.78, 130.84, 130.27, 128.75, 127.81, 127.23, 126.22, 122.46, 115.08, 102.38, 55.89	365.1263 (364.1147)
**9d (VS183D)**	3394, 3106, 3046, 2849, 1707, 1605, 1198, 1105	6.18 (s, 1H, enolic OH), 6.95–7.05 (m, 2H, Ar), 7.50 (d, 2H, Ar, *J* = 4), 7.61–7.66 (m, 2H, Ar), 7.75–7.79 (m, 7H, Ar), 9.10 (s, 1H, NH), 11.30 (s, 1H, OH)	184.02, 183.24, 164.00, 139.80, 139.74, 135.39, 134.14, 130.63, 129.59, 128.84, 127.99, 126.29, 125.62, 102.86	369.0768 (368.0645)
**9e (VS186A)**	3304, 3019, 2938, 1636, 1570, 1286, 1159	3.84 (s, 3H, OCH_3_), 6.15 (s, 1H, enolic OH), 6.81–6.84 (m, 1H, Ar), 6.90–7.05 (m, 2H, Ar), 7.19 (d, 1H, Ar, *J* = 8), 7.35 (s, 1H, Ar), 7.59–7.63 (m, 2H, Ar), 7.77–7.82 (m, 6H, Ar), 9.12 (s, 1H, NH), 9.74 (s, 1H, Ar-OH), 11.32 (s, 1H, OH)	186.09, 181.35, 164.12, 150.34, 148.83, 142.58, 138.92, 134.40, 128.80, 127.94, 126.86, 124.28, 121.69, 116.31, 112.00, 102.31, 56.00	381.1212 (380.1132)
**9f (VS186E)**	3010, 2967, 1683, 1586, 1234, 1127	3.67 (s, 3H, OCH_3_), 3.80 (s, 6H, OCH_3_), 6.15 (s, 1H, enolic OH), 6.86–7.06 (m, 4H, Ar), 7.52–7.64 (m, 2H), 7.71–7.77 (m, 5H, Ar), 9.08 (s, 1H, NH), 11.28 (s, 1H, OH)	199.73, 184.82, 182.52, 176.23, 163.94, 153.65, 141.70, 140.06, 137.81, 130.73, 128.79, 128.53, 127.98, 127.95, 126.32, 124.21, 106.54, 102.50, 102.39, 60.68, 56.56	425.1475 (424.1395)
**9g (VS169B)**	3533, 3157, 3026, 2961, 1708, 1613, 1420, 1206, 1160	6.23 (s, 1H, enolic OH), 7.09 (d, 2H, ethylene, *J* = 16), 7.70 (d, 2H, Ar, J = 16), 7.81 (s, 9H, Ar), 9.13 (s, 2H, NH), 11.32 (s, 2H, OH)	183.09, 163.48, 139.42, 137.20, 133.90, 129.82, 128.33, 127.45, 125.80, 102.43	394.1165 (393.1084)

## Data Availability

All data are included in the manuscript and Supplementary Materials.

## Supplementary Materials

The following supporting information can be downloaded at: https://www.mdpi.com/article/10.3390/ijms262311354/s1.

## Abbreviations

The following abbreviations are used in this manuscript:

HDACs	histone deacetylases
DNS	Differential Nuclear Staining
RNA	Ribonucleic acid
SCI	Selective Cytotoxicity Index
ROS	Reactive oxygen species
JC-1	5,5′,6,6′-Tetrachloro-1,1′,3,3′-tetraethylbenzimidazolocarbocyanine iodide
CMap	Connectivity Map
DNA	Deoxyribonucleic acid
SAHA	Vorinostat
FDA	Food and Drug Administration
NCEs	new chemical entities
IUPAC	International Union of Pure and Applied Chemistry
GF254	SILICA GEL GF 254
TLC	thin-layer chromatography
FTIR	Fourier Transform Infrared Spectroscopy
NMR	Nuclear Magnetic Resonance
DMSO-d6	Deuterated dimethyl sulfoxide
CDCl3	deuterated chloroform
TMS	Tetramethylsilane
HR-MS	High-Resolution Mass Spectrometry
HCl	Hydrochloric acid
DMF	Dimethylformamide
EtOH	Ethanol
KBr	Potassium bromide
RPMI	Roswell Park Memorial Institute
DMEM	Dulbecco’s Modified Eagle’s Medium
FBS	fetal bovine serum
CO_2_	Carbon dioxide
PI	Propidium Iodide
H_2_O_2_	hydrogen peroxide
FITC	Fluorescein Isothiocyanate
DCFDA	6-carboxy-2′,7′-dichlorodihydrofluorescein diacetate
PBS	Phosphate-Buffered Saline
CC_50_	50% cytotoxic concentration
IC_50_	half-maximal inhibitory concentration
mol	Mole
mL	Milli Litre
min	Minute
°C	Degree Celsius
b.p.	Boiling point
m.p.	Melting point
rt	Room temperature
pH	potential of Hydrogen
*w*/*v*	weight by volume
mM	Milli molar
%	Percentage
ppm	Parts per million
h	Hour
µM	Micro molar
SD	Standard deviation
TSA	Trichostatin A
EMT	epithelial-to-mesenchymal transition
HIA	high gastrointestinal absorption
CNS	Central Nervous system
P-gp	P-glycoprotein
