# Supplementary material for: Functional Characterization of VS-186B, a Novel HDAC Inhibitor with Anticancer Activity
Source: Int J Mol Sci. 2025 Nov 24;26(23):11354. doi: 10.3390/ijms262311354 (PMC12692360; doi:10.3390/ijms262311354)
Supplement: Supplementary file 1 [file ijms-26-11354-s001.zip › ijms-3923980-supplementary.pdf]

Article

Functional Characterization of VS-186B, a Novel HDAC Inhibitor with Anticancer Activity

Laura A. Sanchez-Michael [1†], Vijayalakshmi S [2,3†], Allison Elias [1], Denisse A. Gutierrez [1], Jose A. Lopez-Saenz [1], Jaqueline Pena-Zacarias [1], Gabriela C. Torres [1], Armando Varela-Ramirez [1], Sujeet Kumar [4], Subhas S. Karki [2*], Renato J. Aguilera [1*]

1 Department of Biological Sciences and the Border Biomedical Research Center, the University of Texas at El Paso. El Paso, TX. 79968 USA; raguilera@utep.edu

2 Department of Pharmaceutical Chemistry, KLE College of Pharmacy, Bengaluru-560010, KLE Academy of Higher Education and Research, Belagavi-590010, Karnataka, India; subhashskarki@gmail.com

3 Department of Pharmaceutical Chemistry, Faculty of Pharmacy, M.S. Ramaiah University of Applied Sciences, Bengaluru, 560054, Karnataka, India; vijayalakshmis.py.ph@msruas.ac.in

4 Department of Pharmaceutical Chemistry, NITTE College of Pharmaceutical Sciences (Nitte-Deemed to be University, Mangaluru), Yelahanka, Bengaluru, Karnataka 560064, India; klempharma@gmail.com

* Correspondence: subhashskarki@klepharmblr.org (S.S.K.), raguilera@utep.edu (R.J.A.)

† Equal first author

Contents:

HRMS spectra of compound **5**	Figure S1
HRMS spectra of compound **7**	Figure S2
Structure and IUPAC name of N-hydroxy-4-((1E,6E)-7-(substitutedphenyl)-3,5-dioxohepta-1,6-dien-1-yl)benzamide (**9a-g**)	Table S1
Physicochemical properties of **9a-g**	Table S2
IR spectra's of **9a-g**	Figure S3-9
^{1}H-NMR spectra's of **9a-g**	Figure S10-16
D_2O exchange spectra of **9g**	Figure S17
^{13}C-NMR spectra's of **9a-g**	Figure S18-24
HRMS spectra's of **9a-g**	Figure S25-31

Contents:

Annexin V/PI flow cytometry plots	Figure S32
ROS flow cytometry plots	Figure S33
JC-1 mitochondrial membrane potential flow cytometry plots	Figure S34
Caspase-3/7 flow cytometry plots	Figure S35
Cell cycle flow cytometry plots	Figure S36
CC_{50} values of all novel HDAC inhibitors in multiple cell lines	Table S3
CMap Analysis Heatmap	Figure S37
BOILED-Egg pharmacokinetic analysis	Figure S38 and Table S4

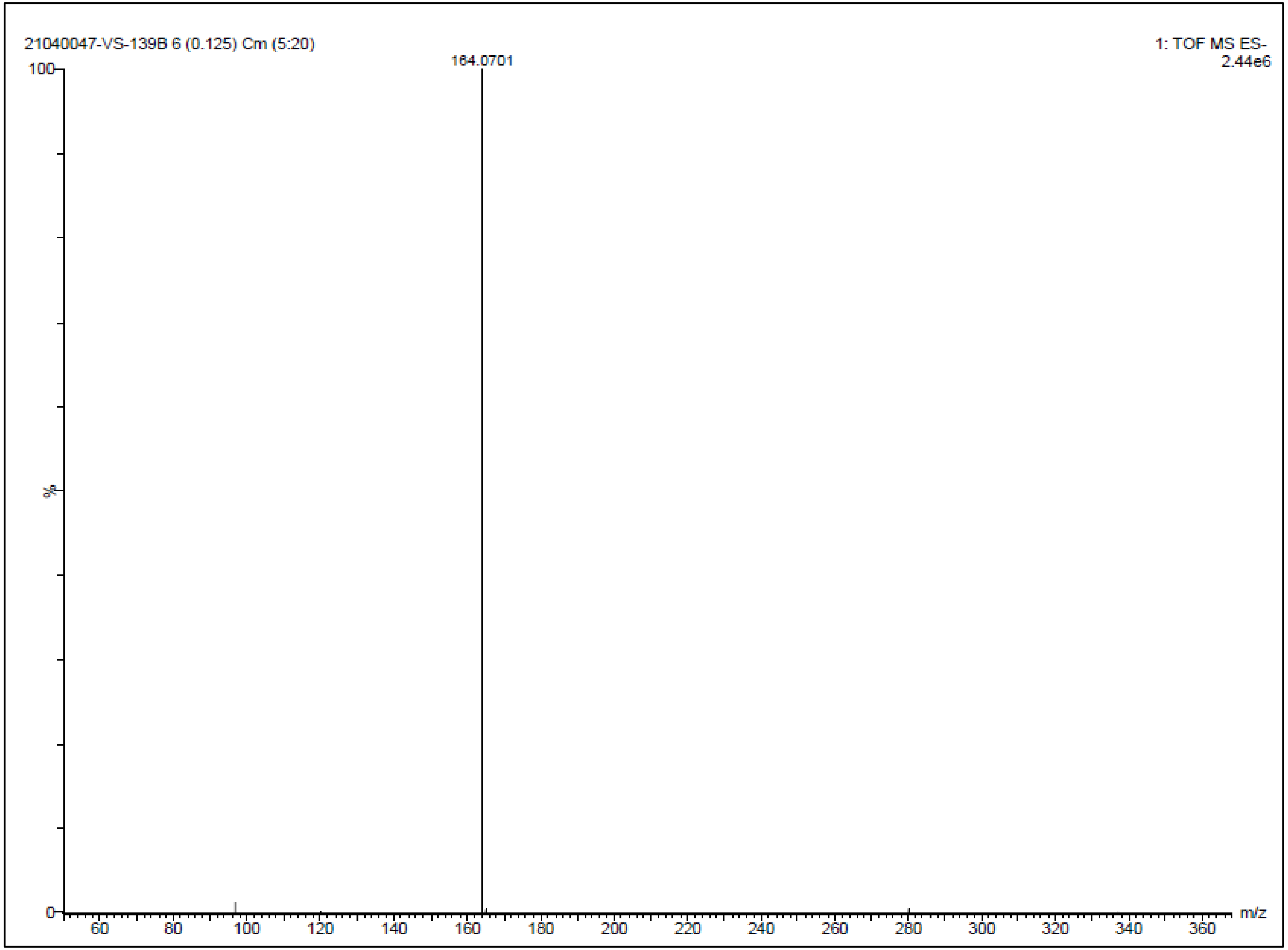

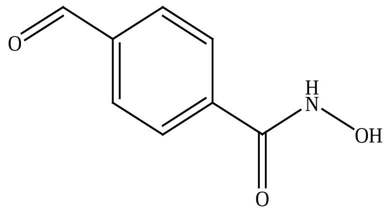

4-formyl-*N*-hydroxybenzamide
Chemical Formula: $C_8H_7NO_3$
calculated Mol weight: 165.0426

5

Figure S1. HRMS spectra of compound 5 / VS-139B

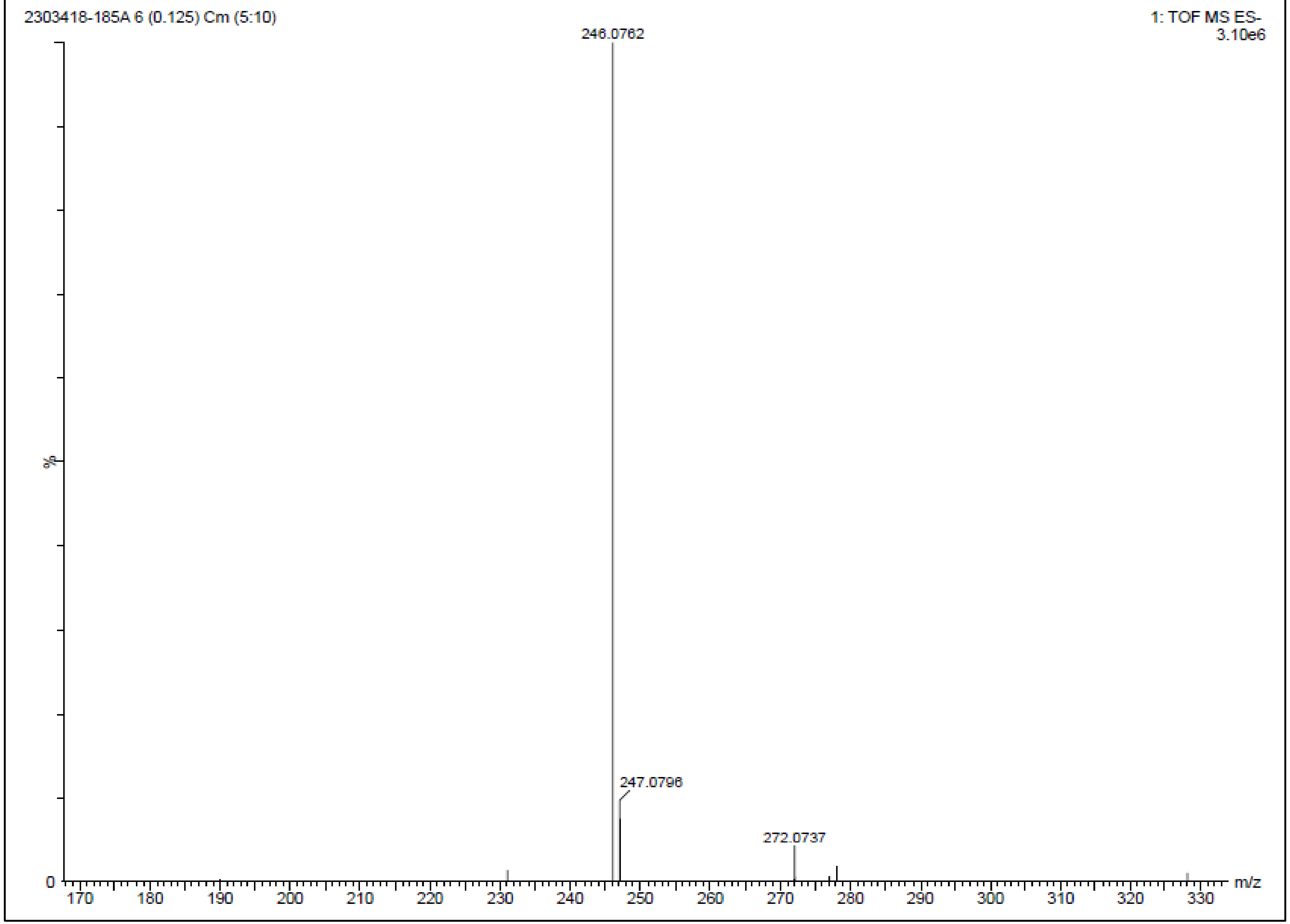

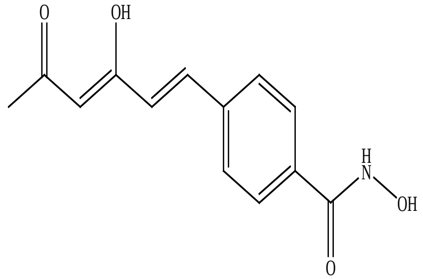

N-hydroxy-4-((1*E*,3*Z*)-3-hydroxy-5-oxohexa-1,3-dien-1-yl)benzamide

Chemical Formula: $C_{13}H_{13}NO_4$

Calculated Mol weight: 247.0845

7

Figure S2. HRMS spectra of compound 7 / VS-185A

Code	Structure and IUPAC name	Code	Structure and IUPAC name
**9a/186C**	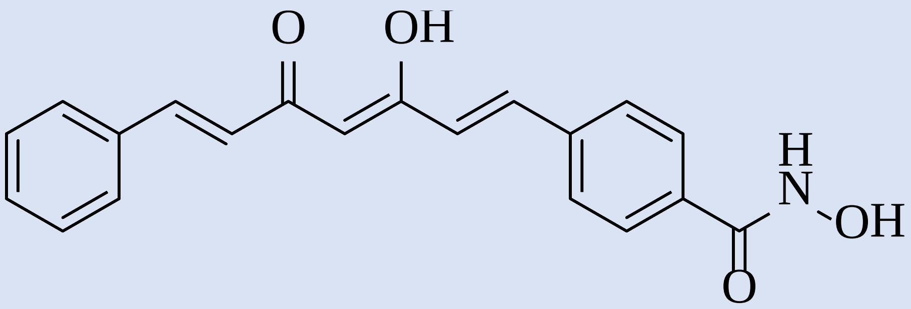<br>*N*-hydroxy-4-((1*E*,3*Z*,6*E*)-3-hydroxy-5-oxo-7-phenylhepta-1,3,6-trien-1-yl)benzamide	**9c/183A**	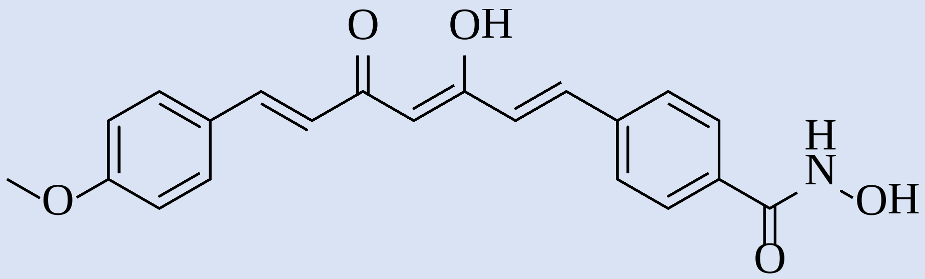<br>*N*-hydroxy-4-((1*E*,6*E*)-7-(4-methoxyphenyl)-3,5-dioxohepta-1,6-dien-1-yl)benzamide
**9b/186B**	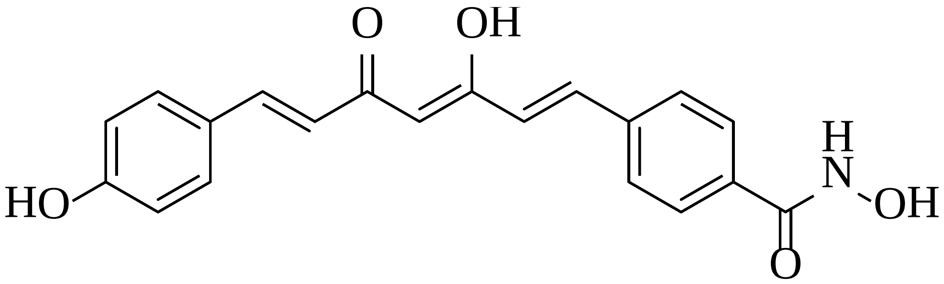<br>*N*-hydroxy-4-((1*E*,3*Z*,6*E*)-3-hydroxy-7-(4-hydroxyphenyl)-5-oxohepta-1,3,6-trien-1-yl)benzamide	**9d/183D**	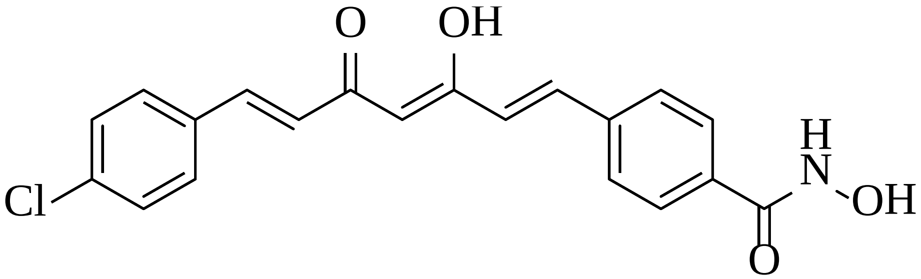<br>4-((1*E*,6*E*)-7-(4-chlorophenyl)-3,5-dioxohepta-1,6-dien-1-yl)-*N*-hydroxybenzamide

Table S1: Structure and IUPAC name of *N*-hydroxy-4-((1*E*,6*E*)-7-(substitutedphenyl)-3,5-dioxohepta-1,6-dien-1-yl)benzamide (9a-g)

Code	Structure and IUPAC name	Code	Structure and IUPAC name
**9e/186A**	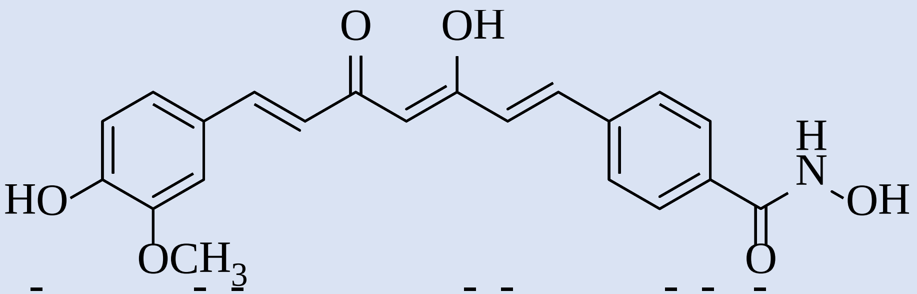<br>*N*-hydroxy-4-((1*E*’3*Z*’6*E*)-3-hydroxy-7-(4-hydroxy-3-methoxyphenyl)-5-oxohepta-1’3’6-trien-1-yl)benzamide	**9g/169B**	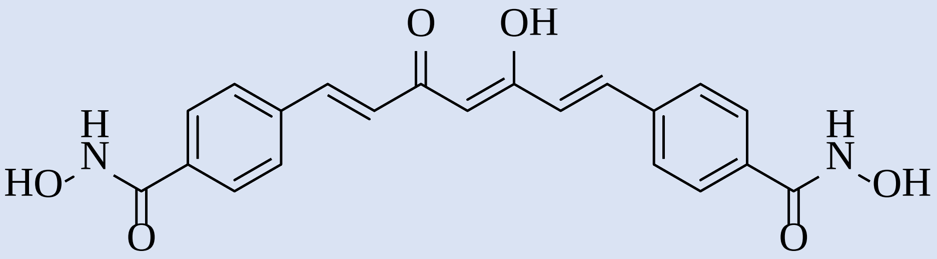<br>4’4'-((1*E*’3*Z*’6*E*)-3-hydroxy-5-oxohepta-1’3’6-triene-1’7-diyl)bis(*N*-hydroxybenzamide)
**9f/186E**	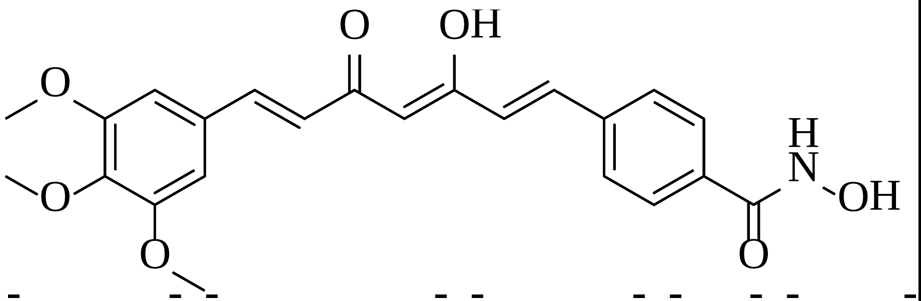<br>*N*-hydroxy-4-((1*E*’3*Z*’6*E*)-3-hydroxy-5-oxo-7-(3’4’5-trimethoxyphenyl)hepta-1’3’6-trien-1-yl)benzamide		

Table S1: Structure and IUPAC name of *N*-hydroxy-4-((1*E*,6*E*)-7-(substitutedphenyl)-3,5-dioxohepta-1,6-dien-1-yl)benzamide (9a-g)

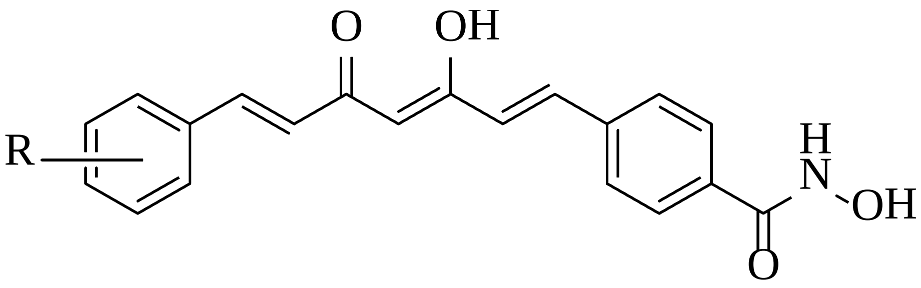

Code	R	Color Of Compound	% Yield	Melting point	Molecular Formula	Molecular weight
**9a / 186C**	H	Light brown	20	216-218	$C_{20}H_{17}NO_4$	335.3533
**9b / 186B**	4-OH	Dark brown	25	186-188	$C_{20}H_{17}NO_5$	351.3527
**9c / 183A**	4-OCH_3	Golden yellow	15	196-198	$C_{21}H_{19}NO_5$	365.3793
**9d / 183D**	4-Cl	Dark yellow	17	204-206	$C_{20}H_{16}ClNO_4$	369.7983
**9e / 186A**	3-OCH_3,4-OH	Wine red	18	232-234	$C_{21}H_{19}NO_6$	381.3787
**9f / 186E**	3,4,5-triOCH_3	Rust brown	20	224-226	$C_{23}H_{23}NO_7$	425.4312
**9g / 169B**	CONHOH	Light brown	18	240-242	$C_{21}H_{18}N_2O_6$	394.3774

Table S2: Physicochemical properties of 9a-g
***9b is 186B which has undergone extensive in vitro studies**

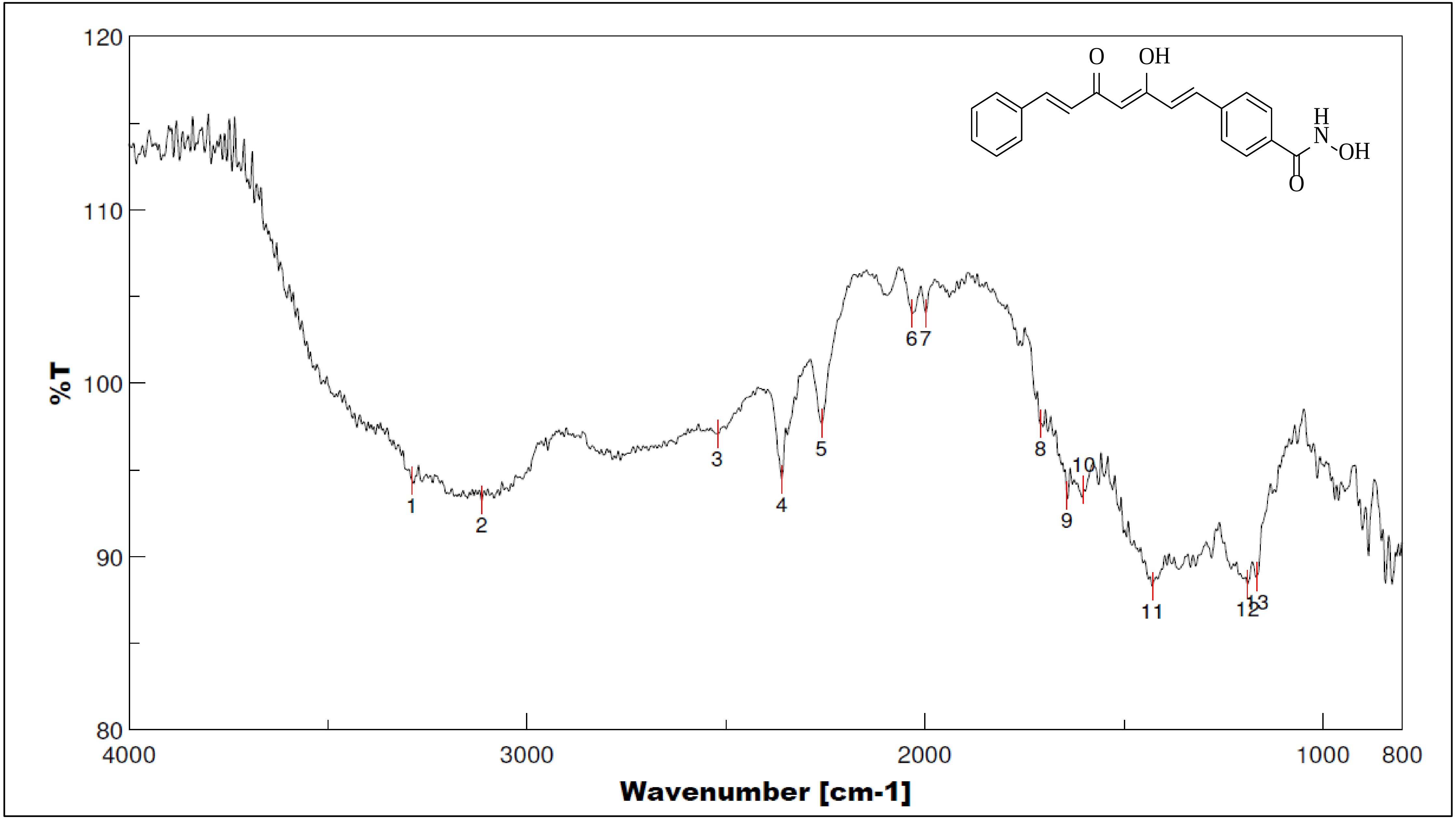

Figure S3. Infrared spectra of 9a / VS-186C

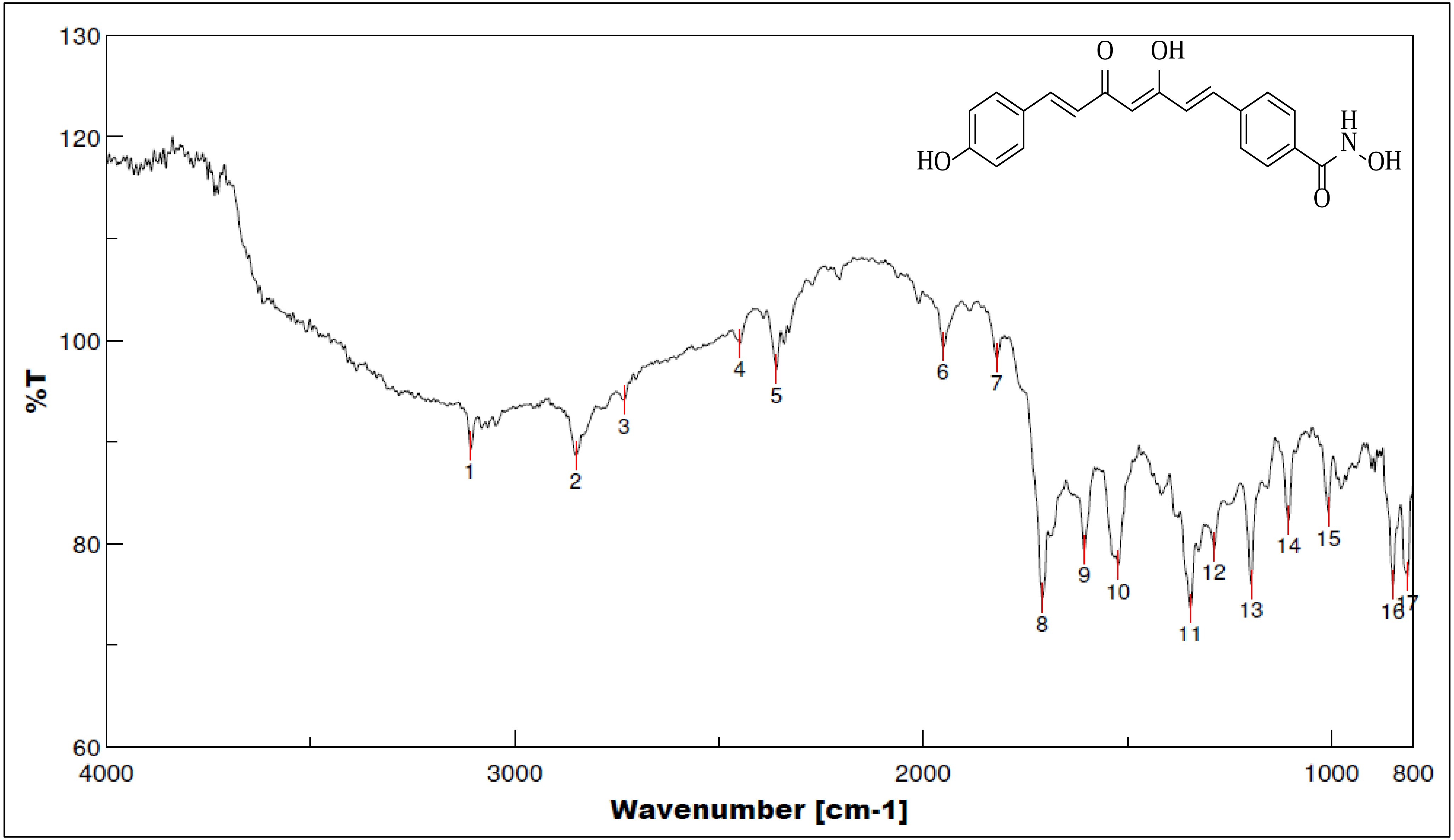

Figure S4. Infrared spectra of 9b / VS-186B

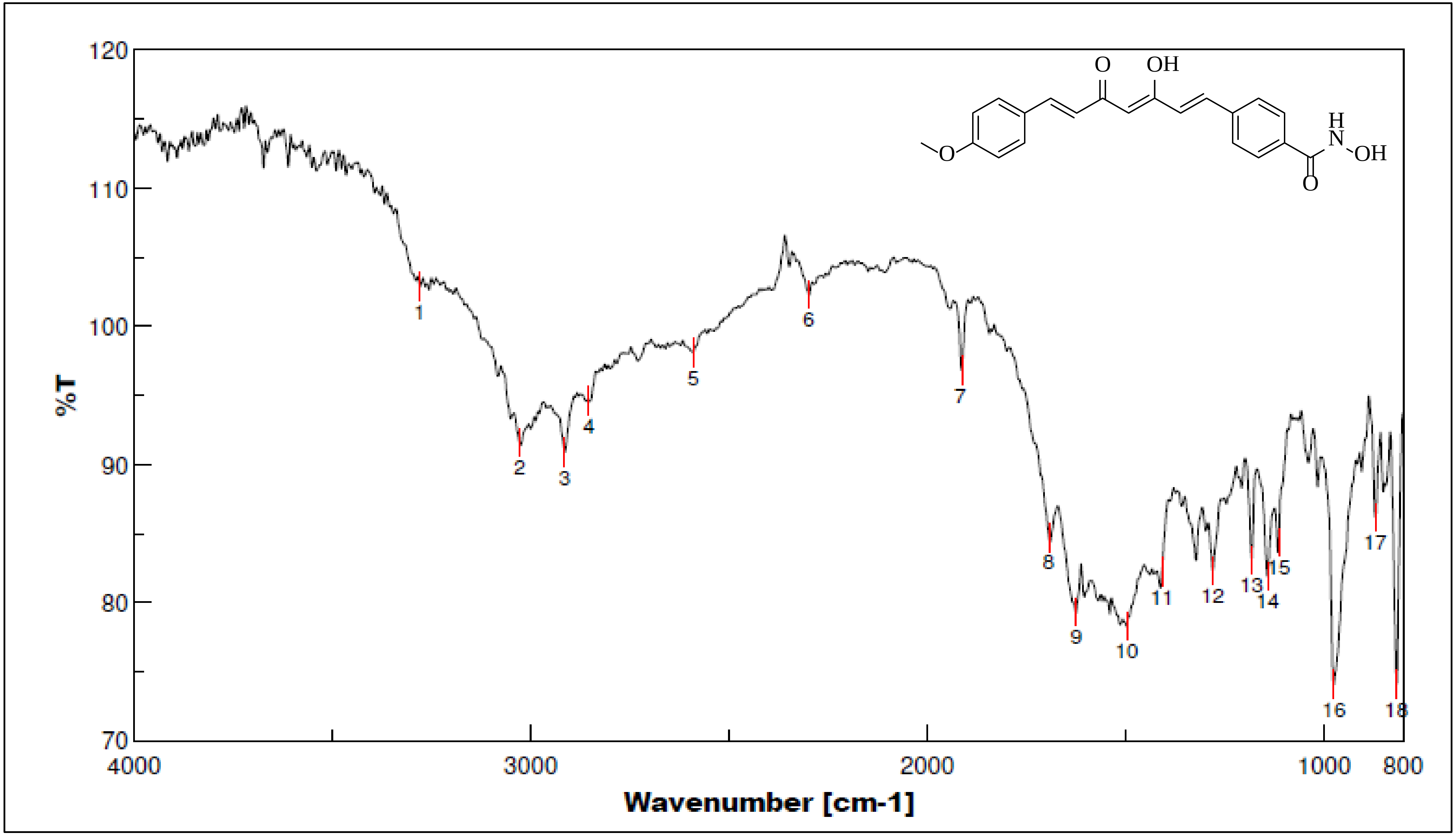

Figure S5. Infrared spectra of 9c / VS-183A

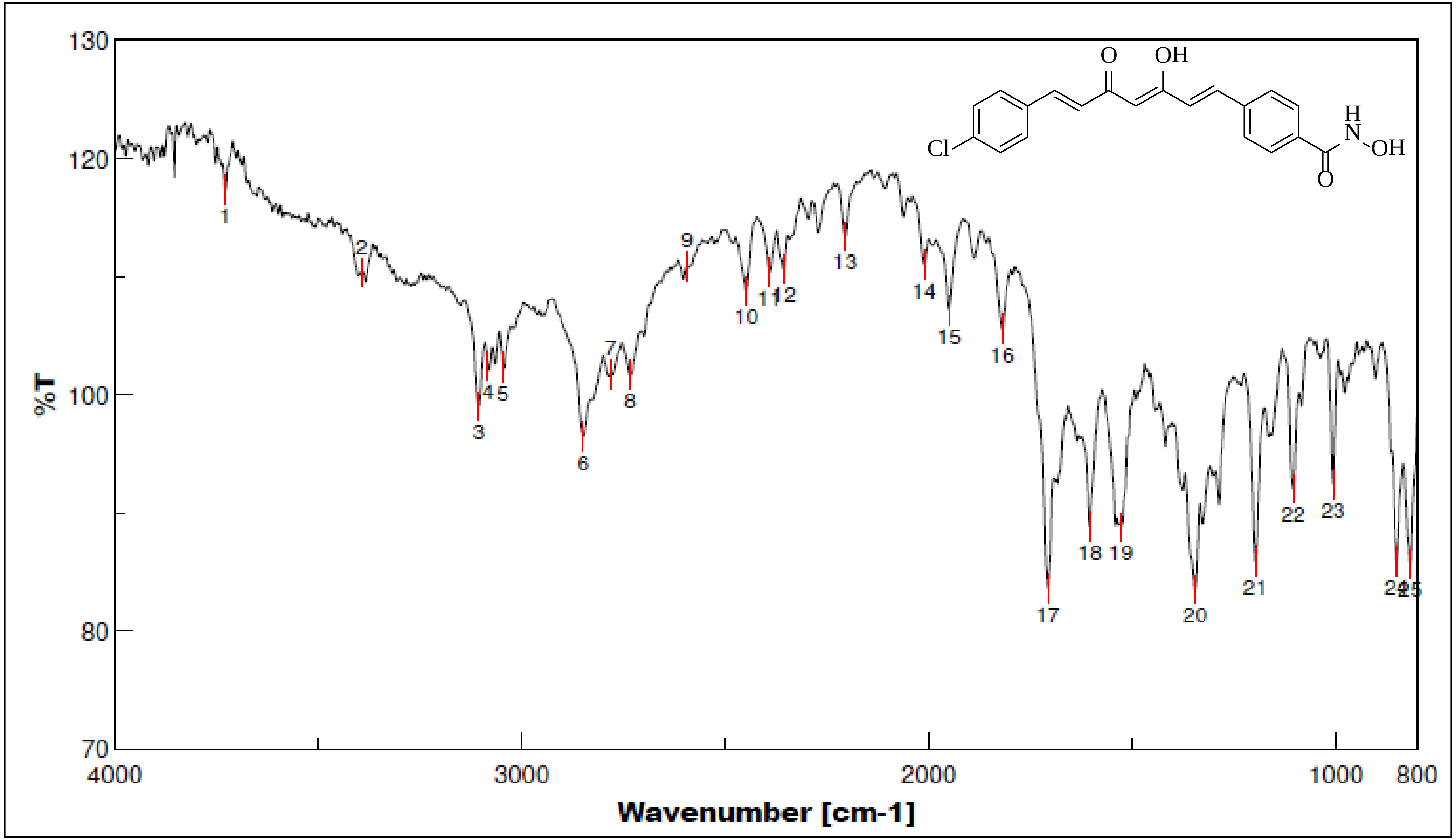

Figure S6. Infrared spectra of 9d / VS-183D

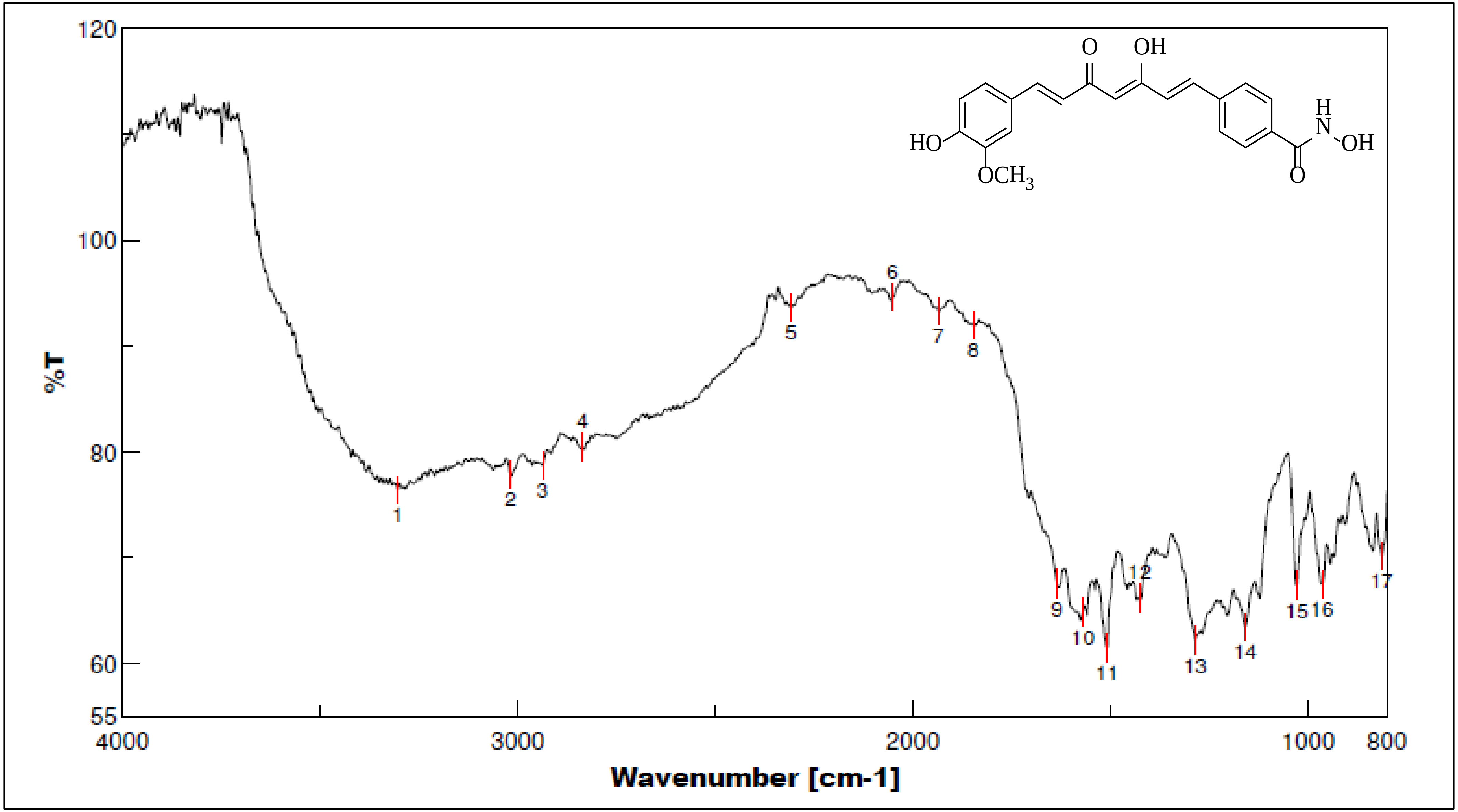

Figure S7. Infrared spectra of 9e / VS-186A

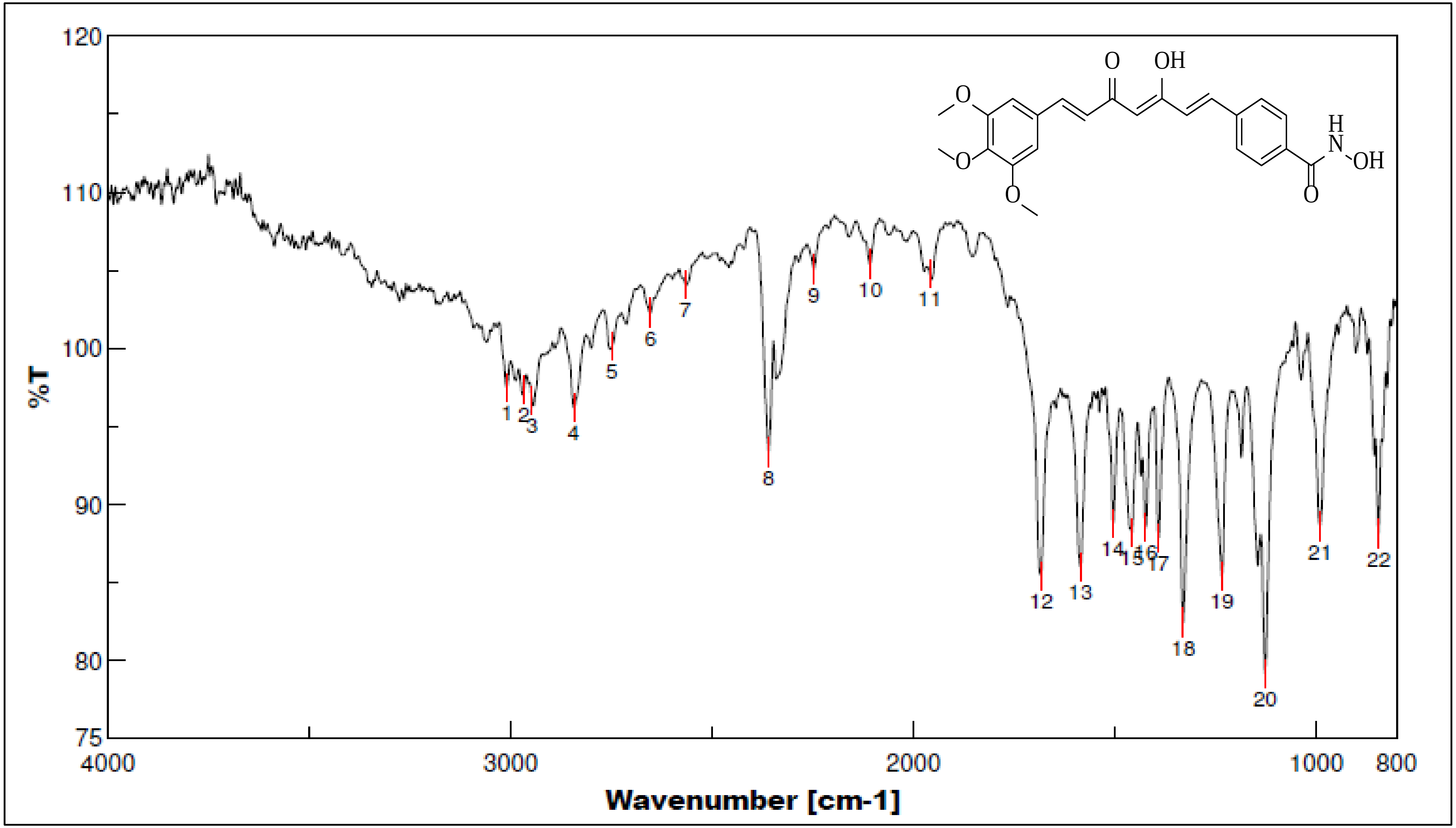

Figure S8. Infrared spectra of 9f / VS-186E

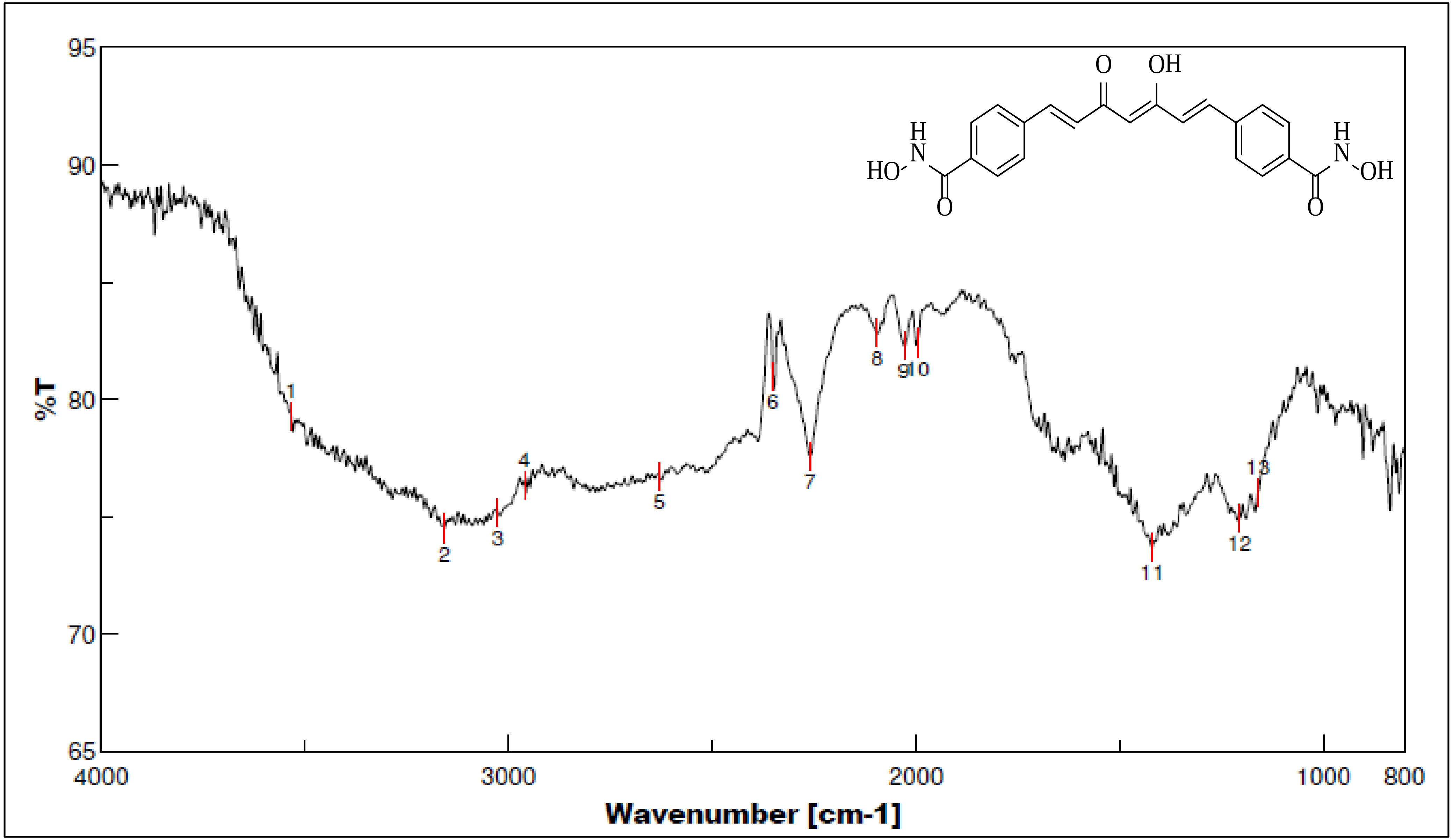

Figure S9. Infrared spectra of 9g / VS-169B

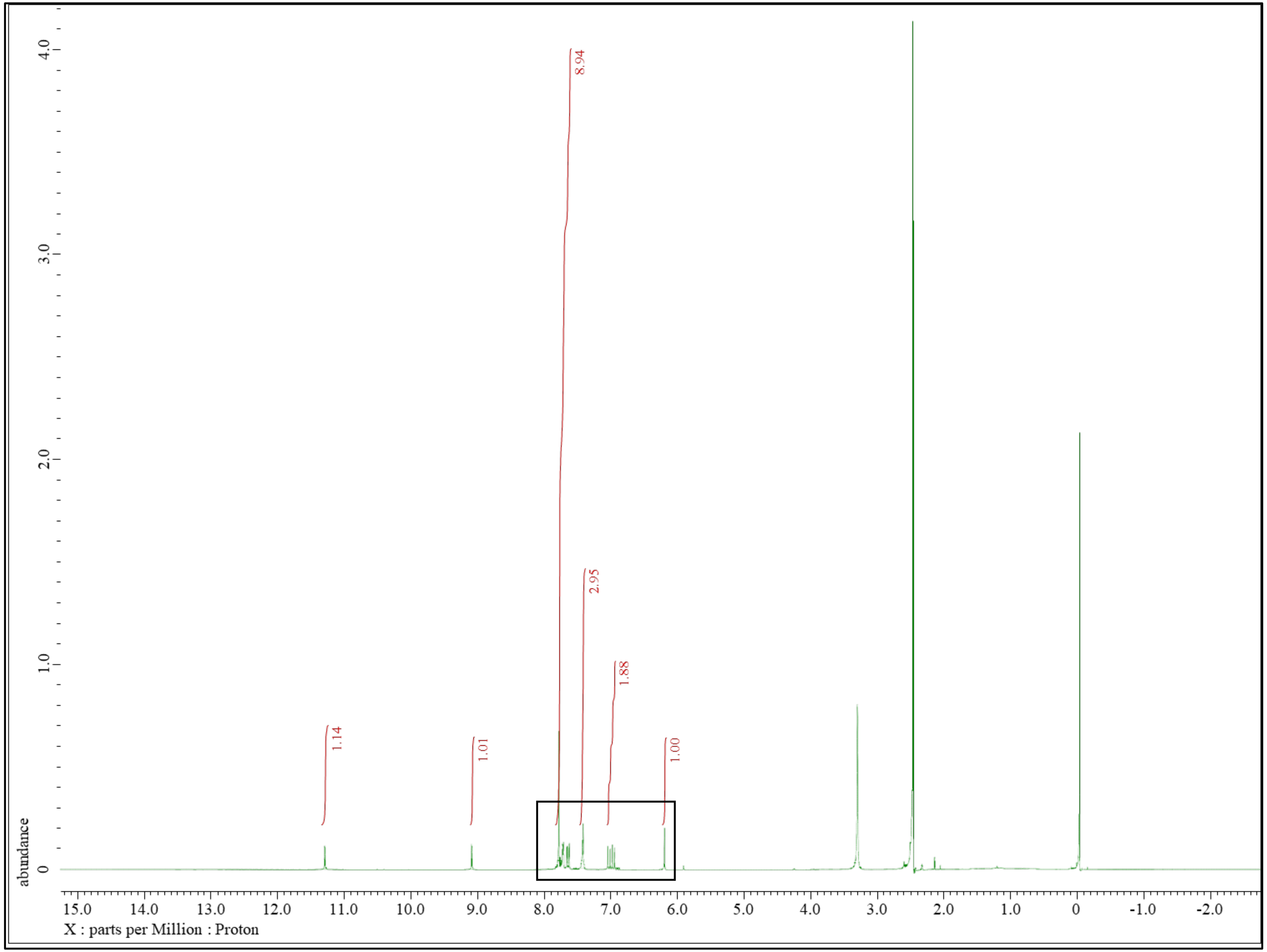

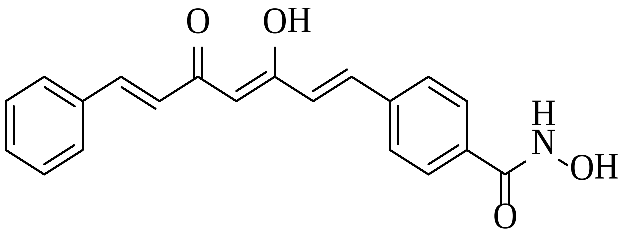

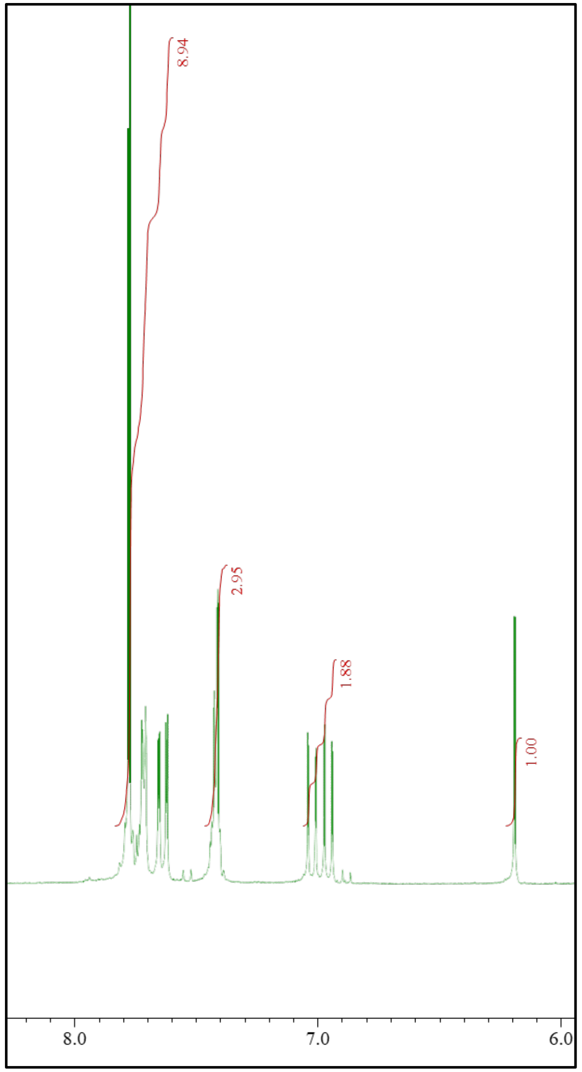

Figure S10. ^{1}H-NMR spectra of 9a / VS-186C

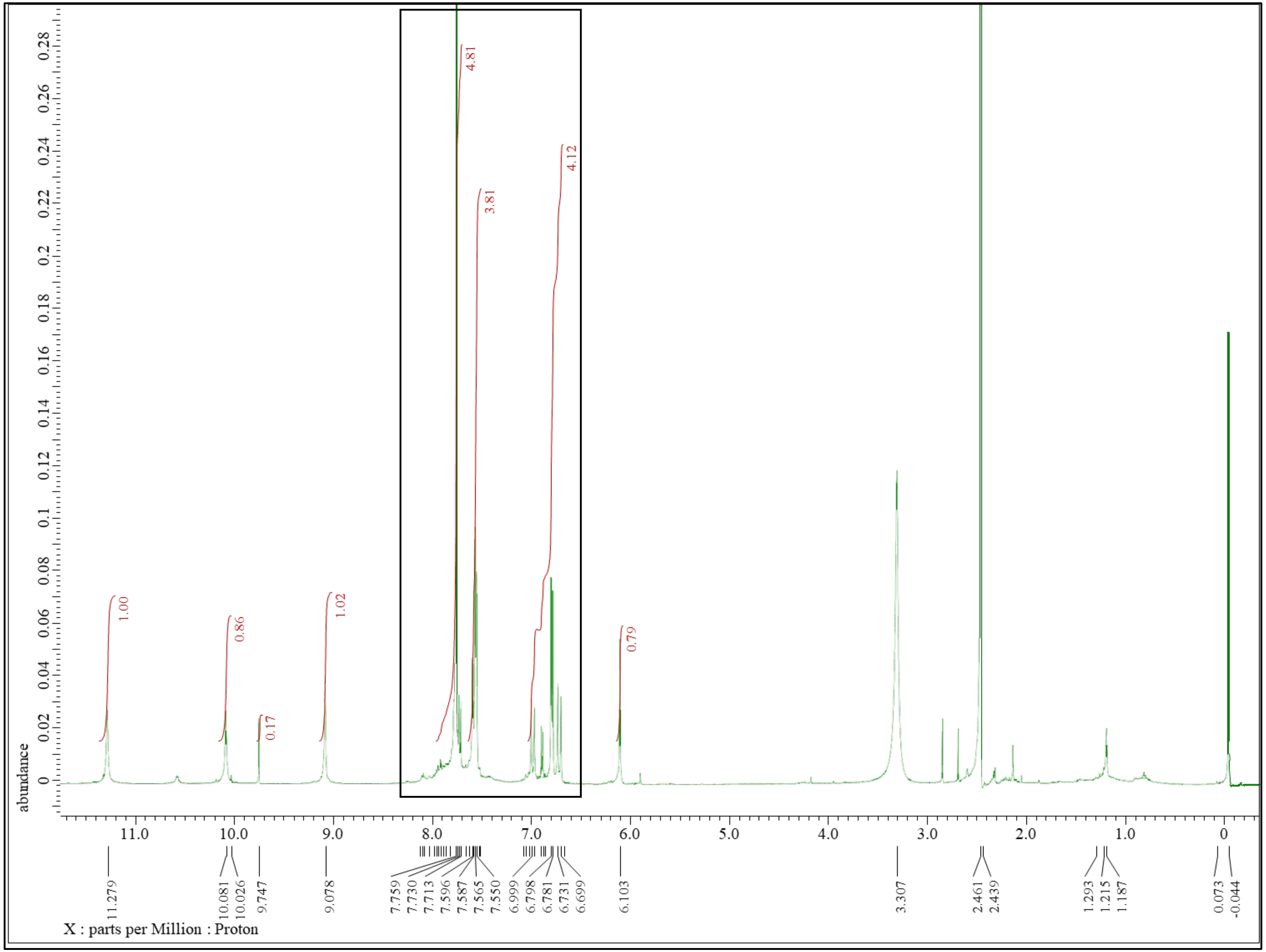

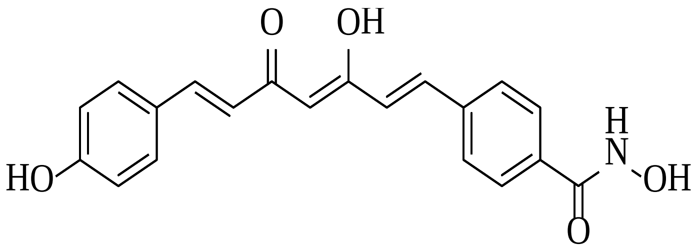

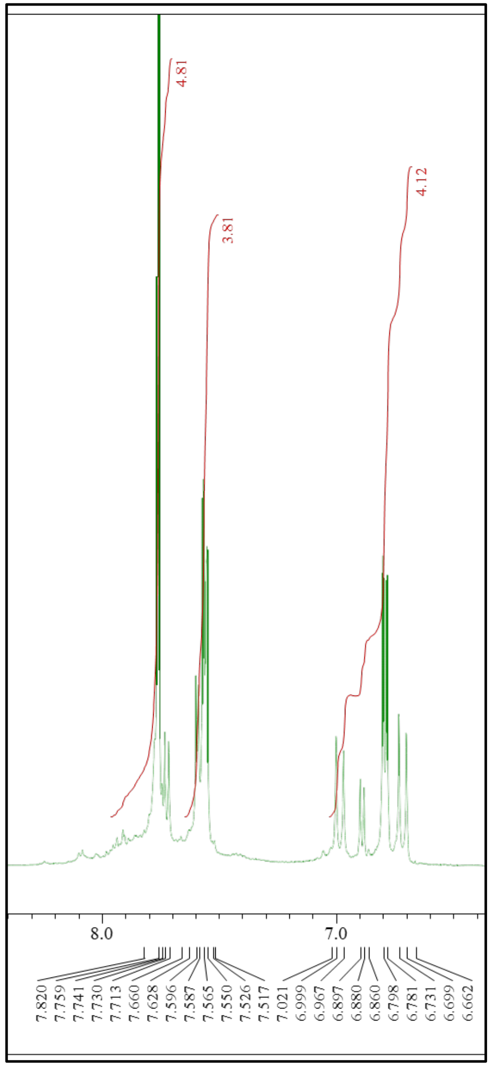

Figure S11. ¹H-NMR spectra of 9b / VS-186B

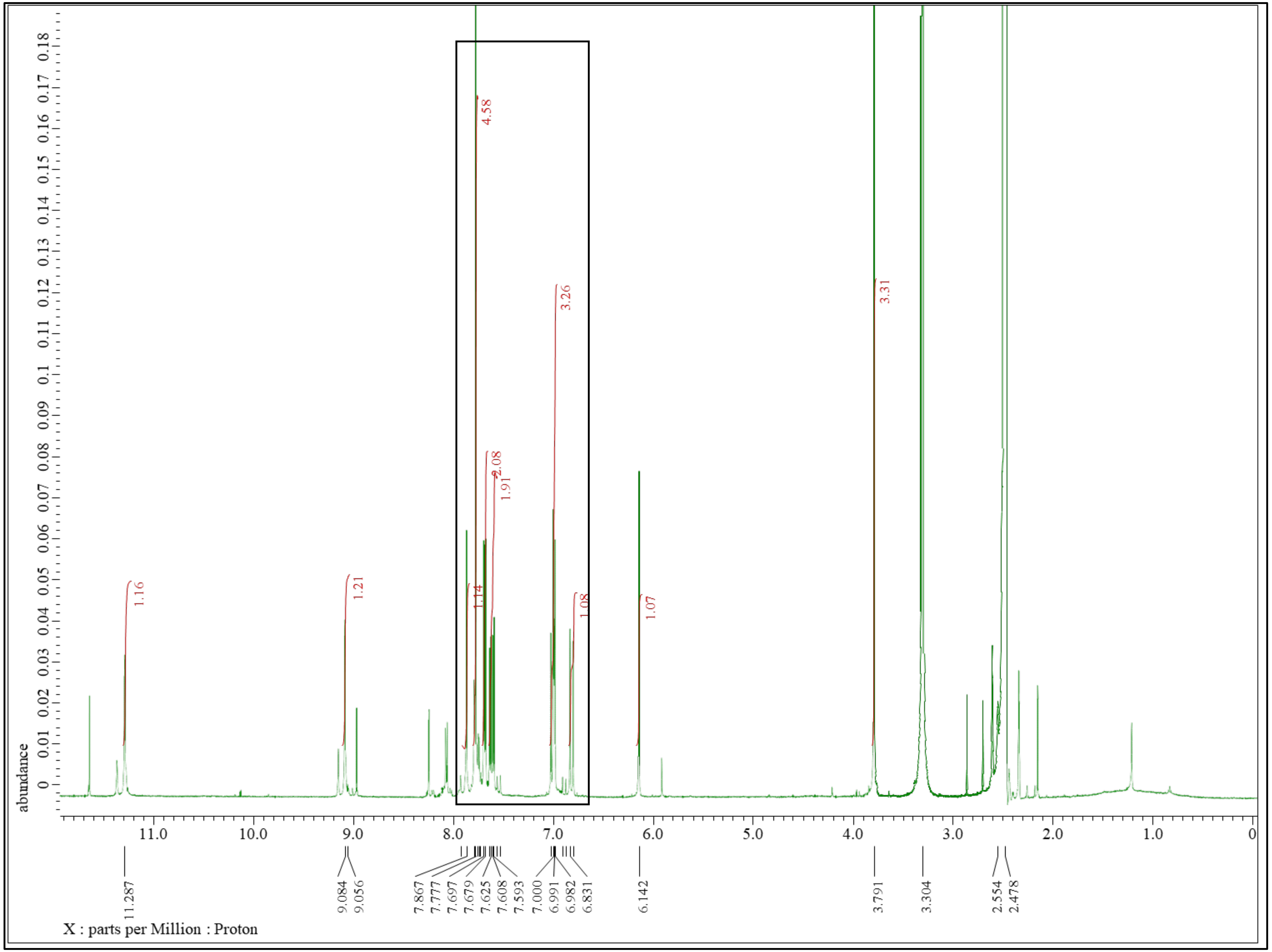

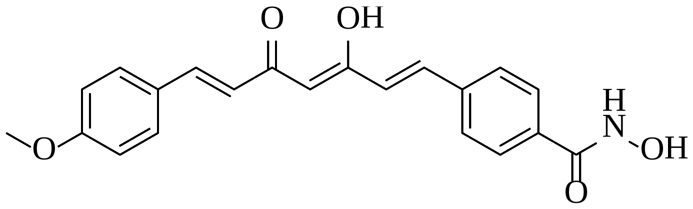

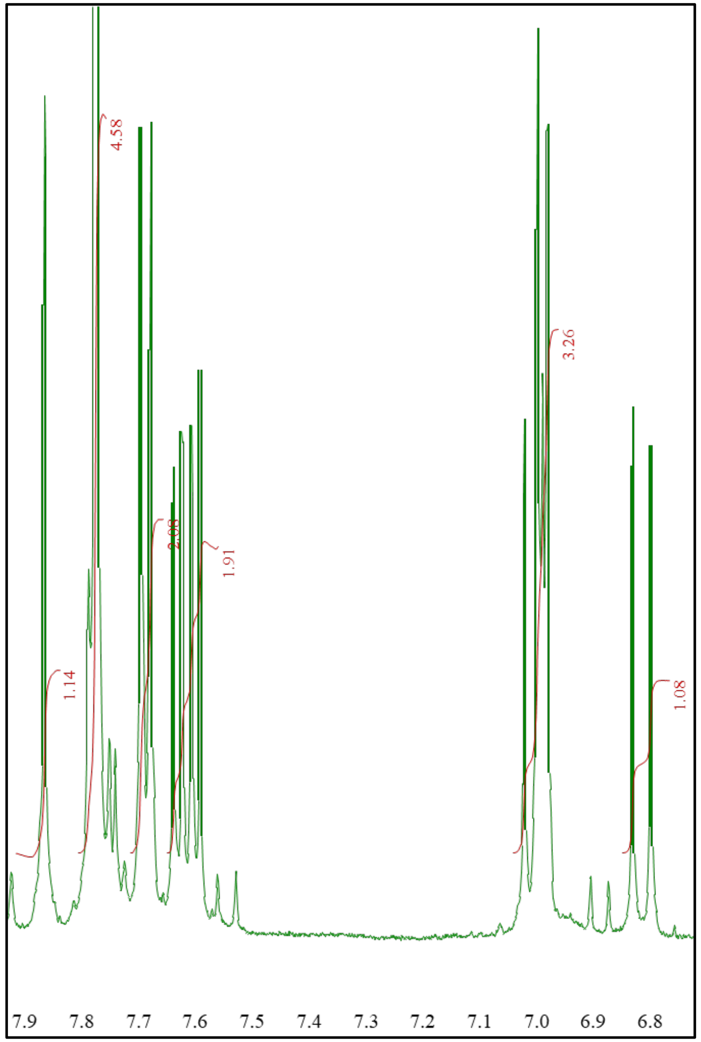

Figure S12. ^{1}H-NMR spectra of 9c / VS-183A

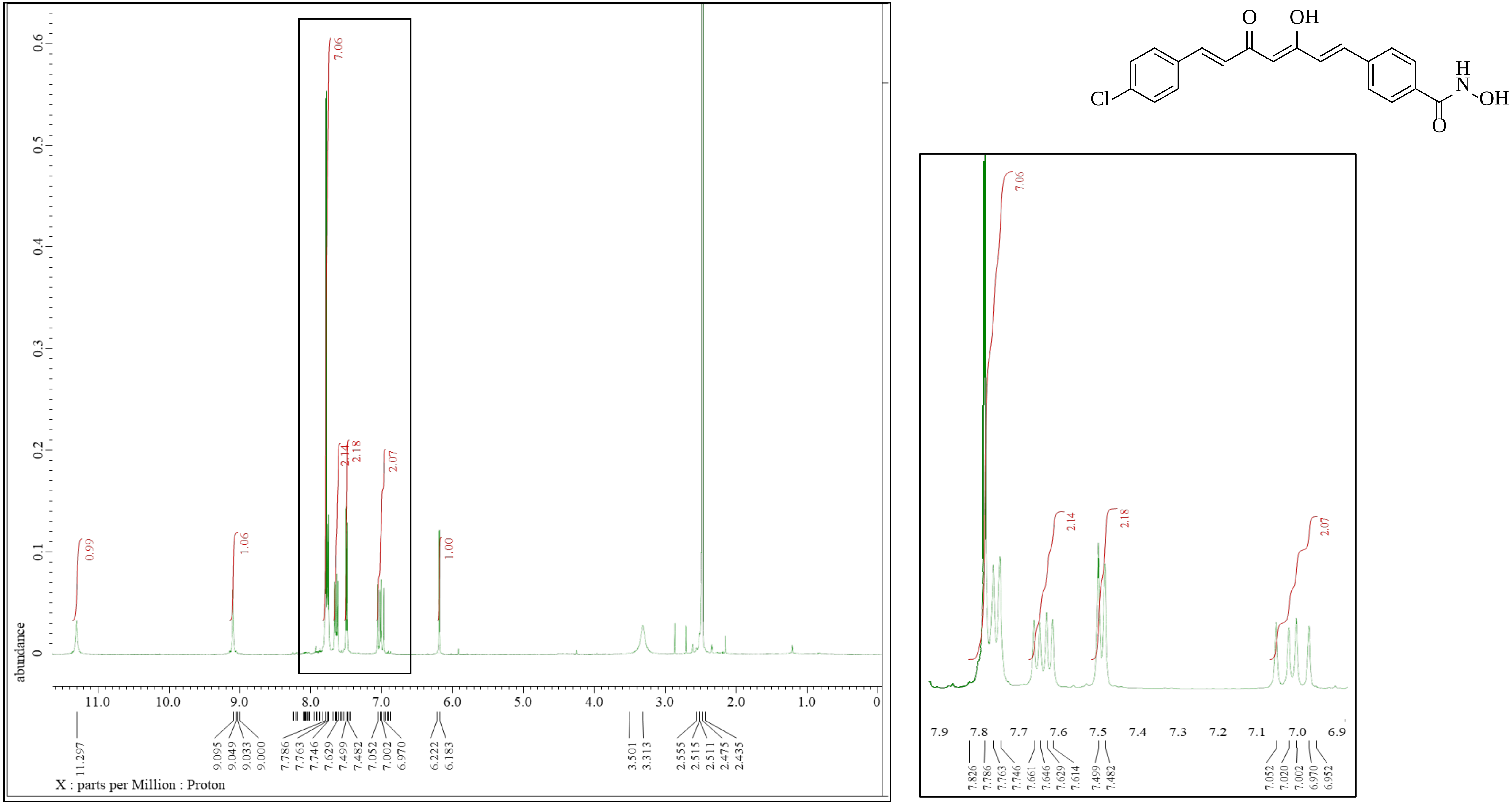

Figure S13. ¹H-NMR spectra of 9d / VS-183D

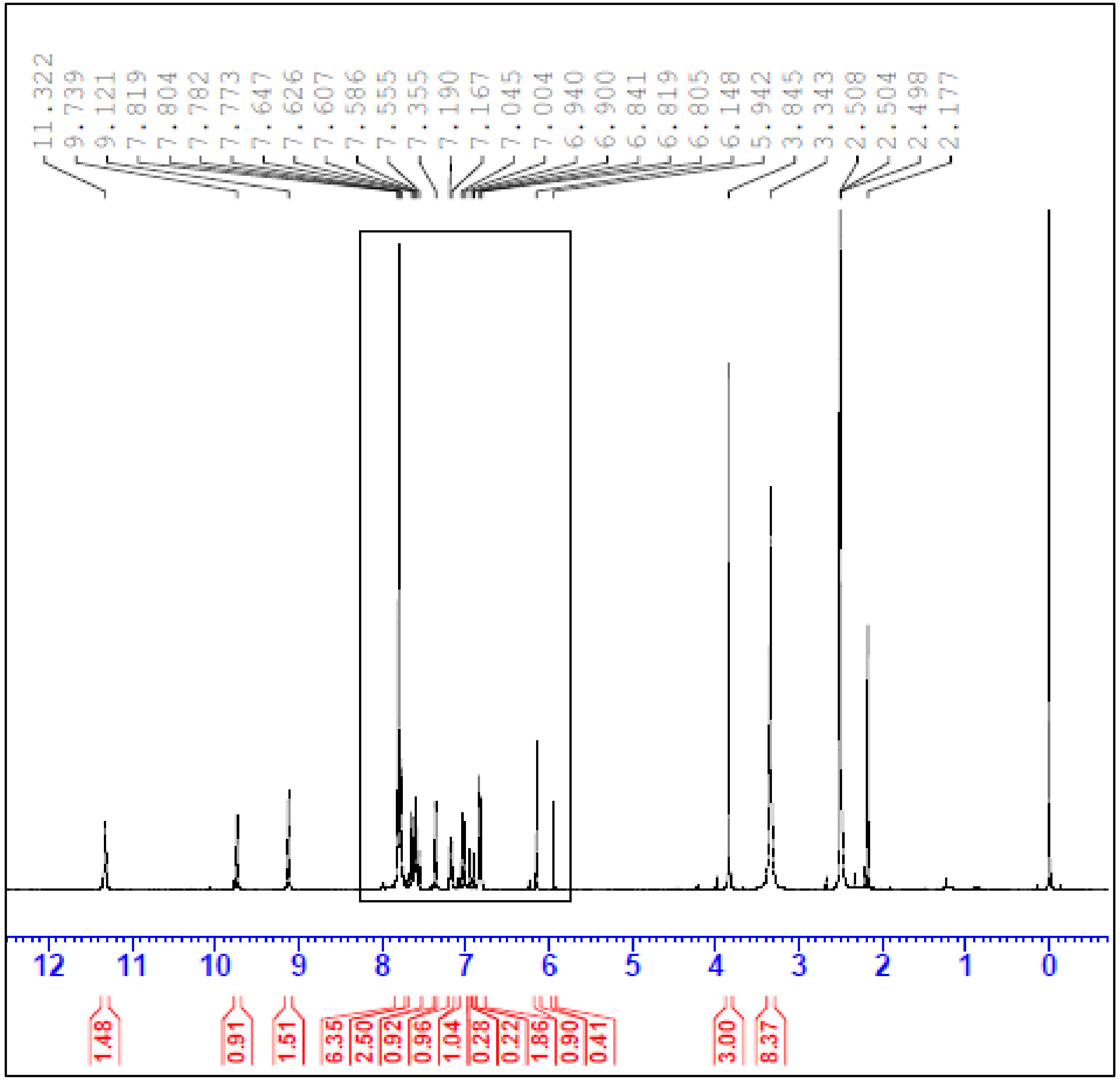

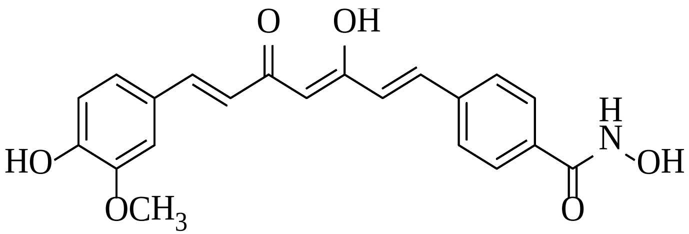

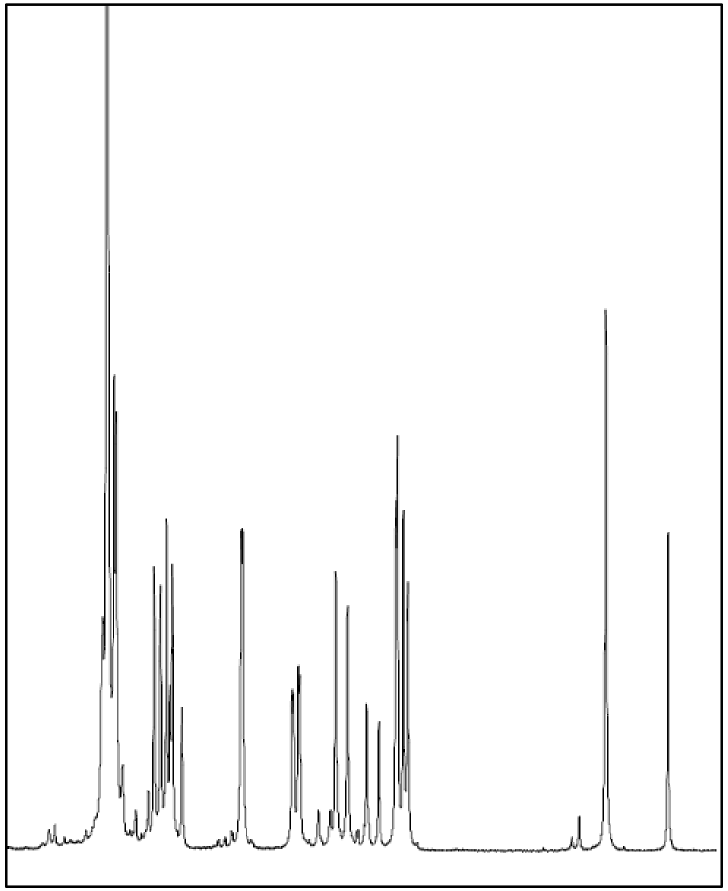

Figure S14. ^{1}H-NMR spectra of 9e / VS-186A

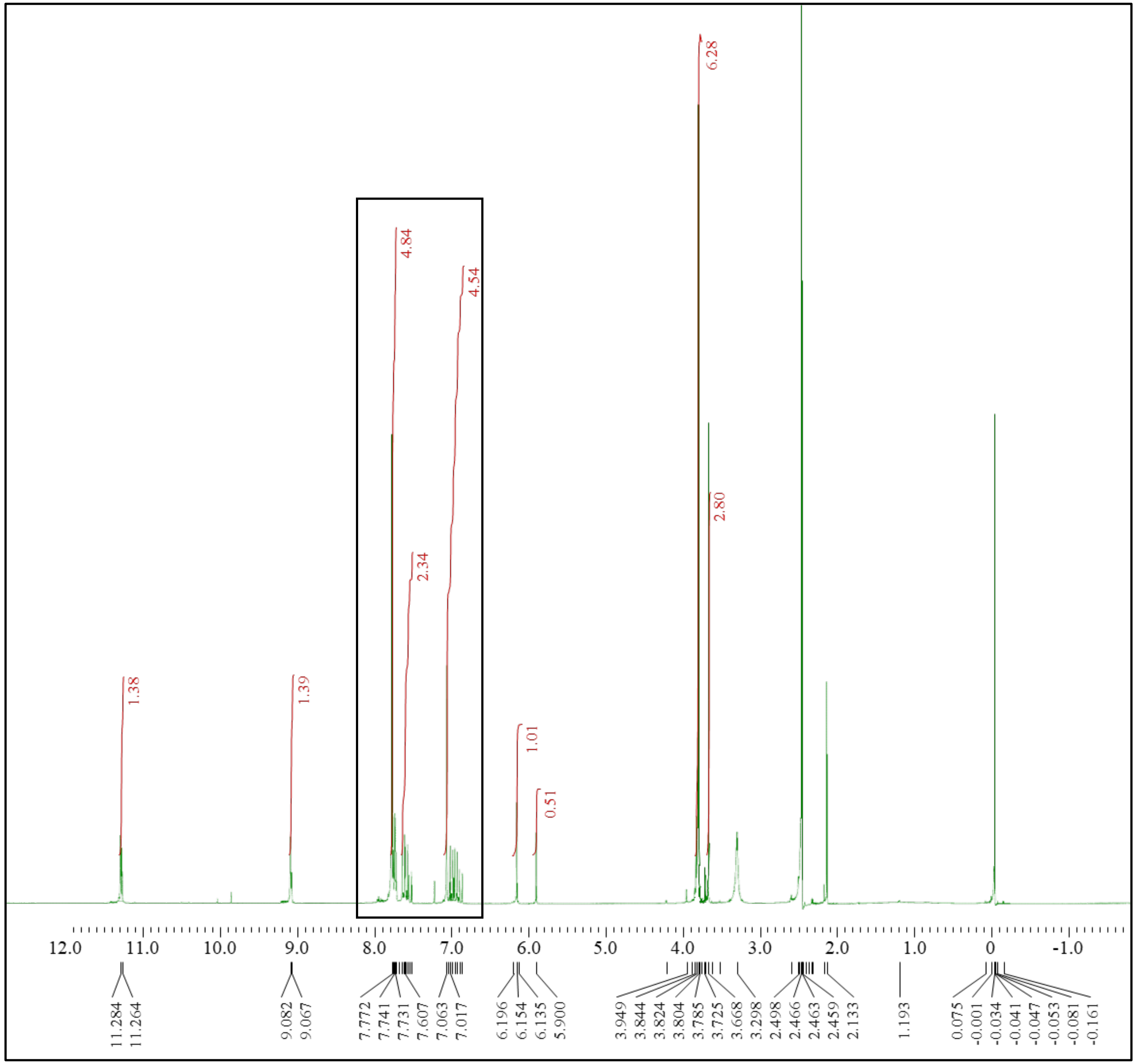

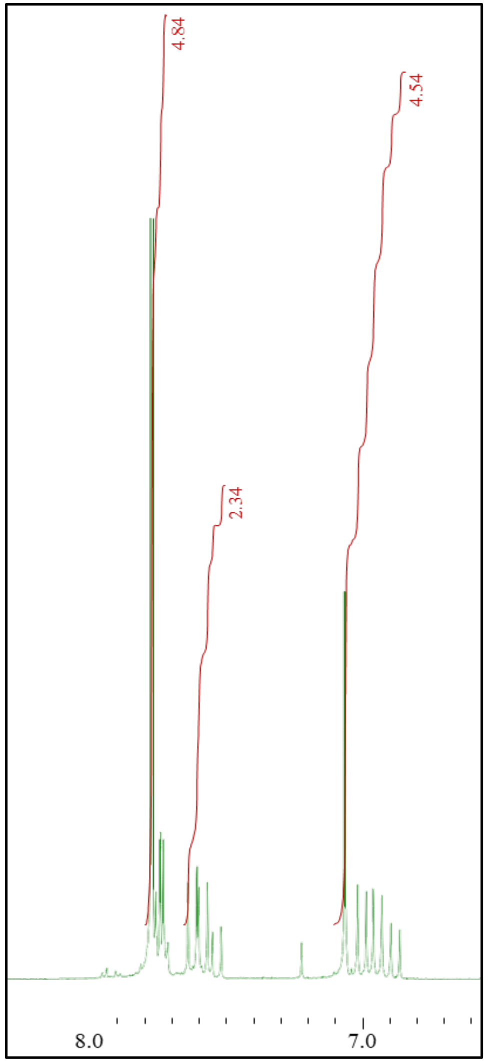

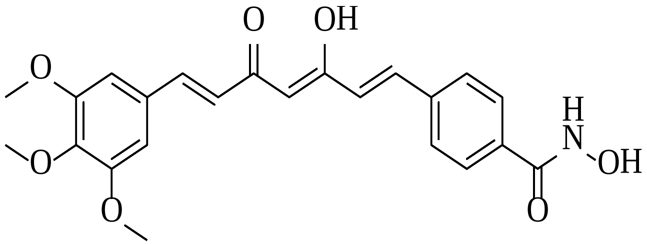

Figure S15. ^{1}H-NMR spectra of 9f / VS-186E

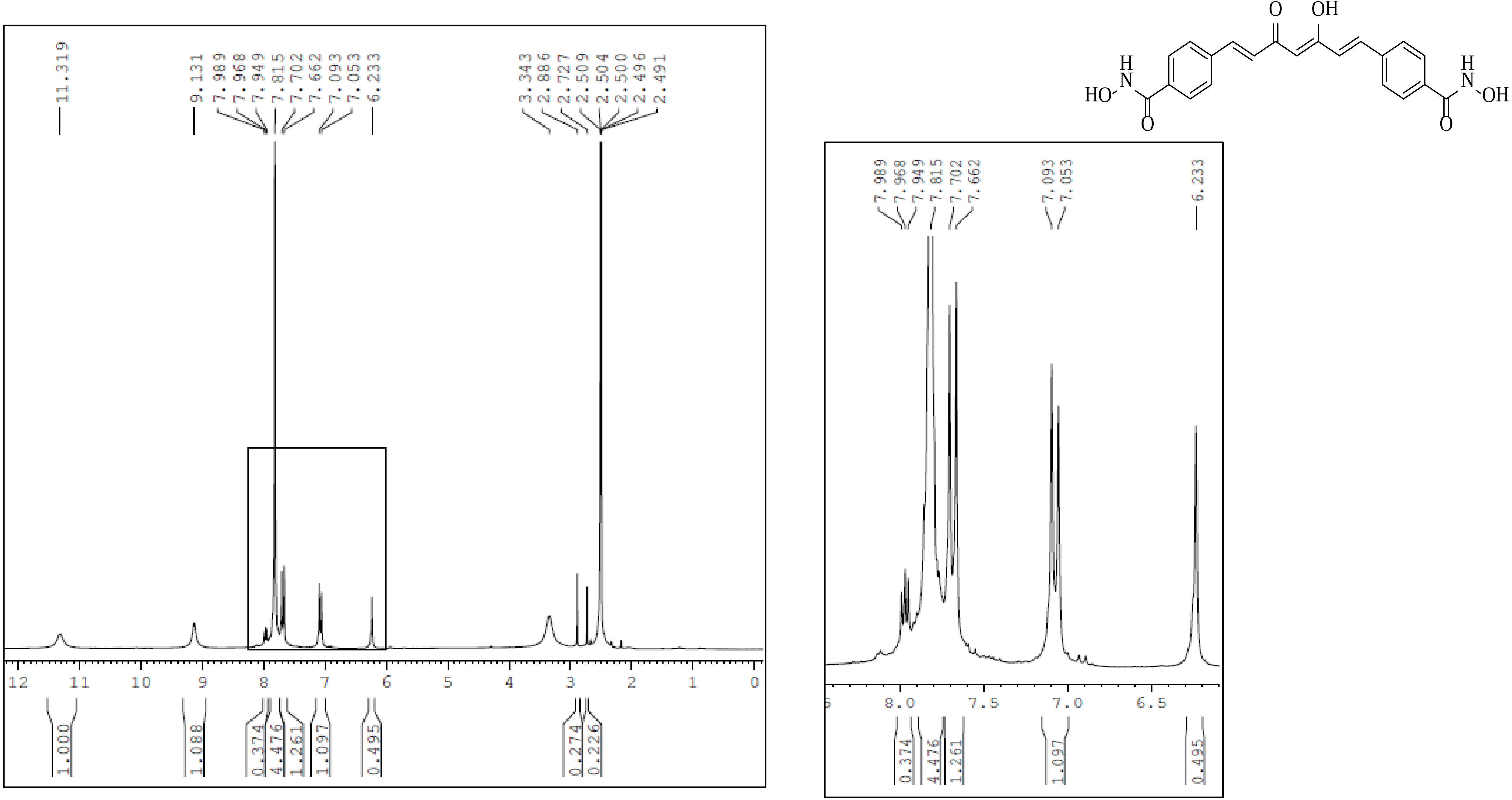

Figure S16. ¹H-NMR spectra of 9g / VS-169B

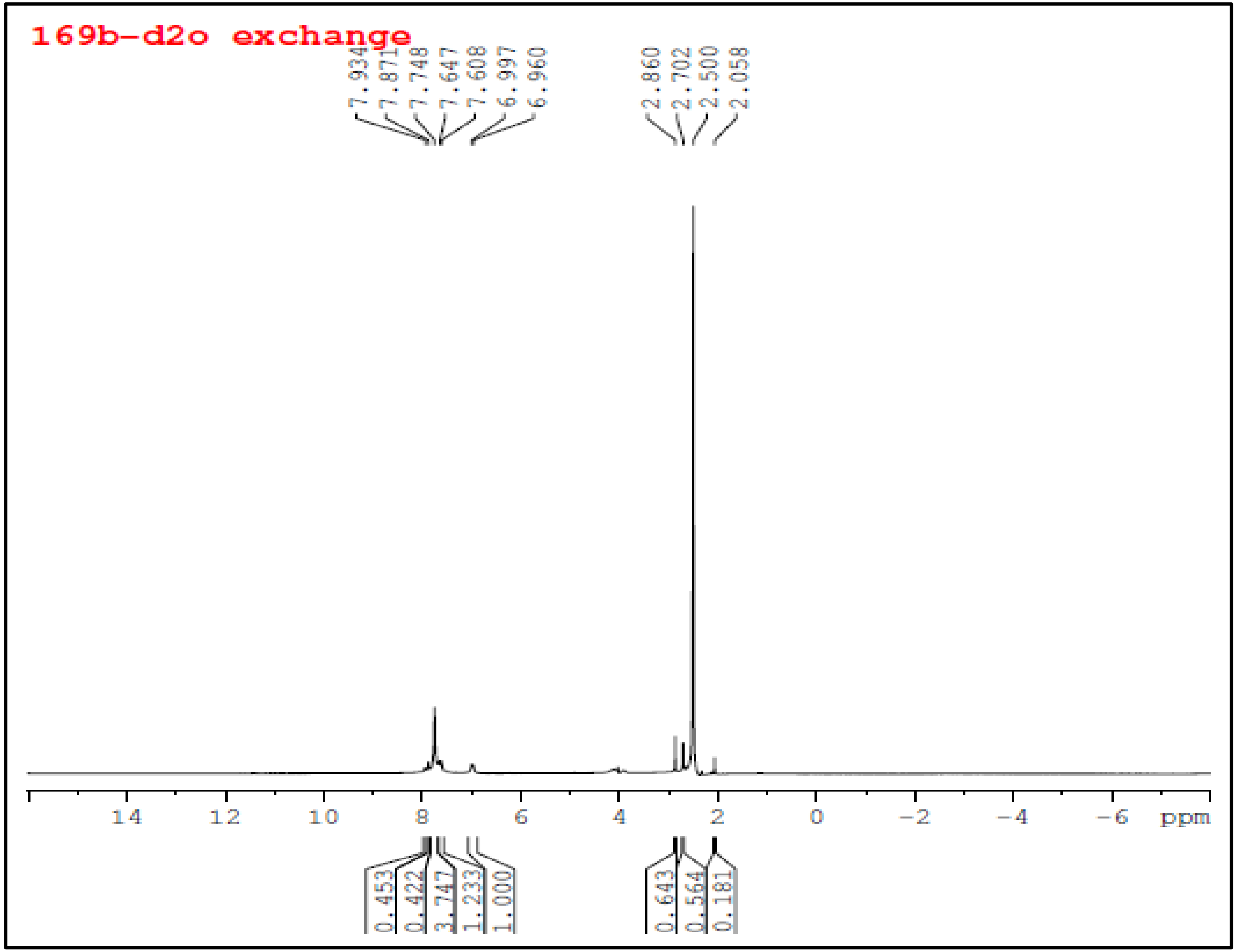

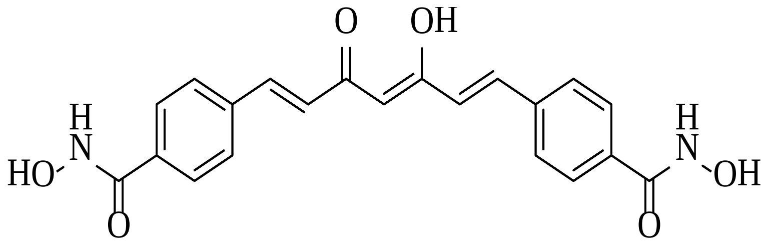

Figure S17. ^{1}H-NMR (D_2O exchange) spectra of 9g / VS-169B

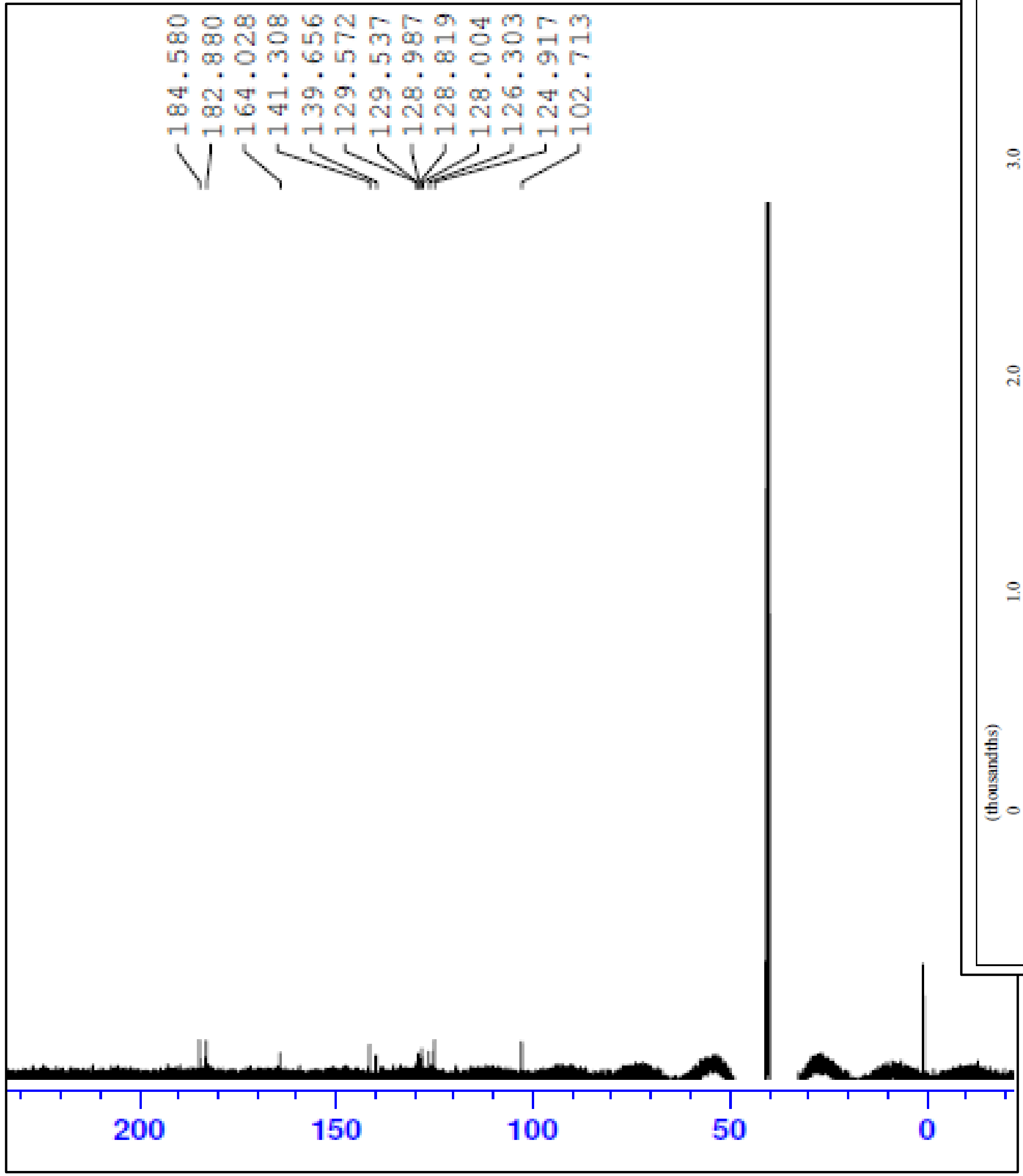

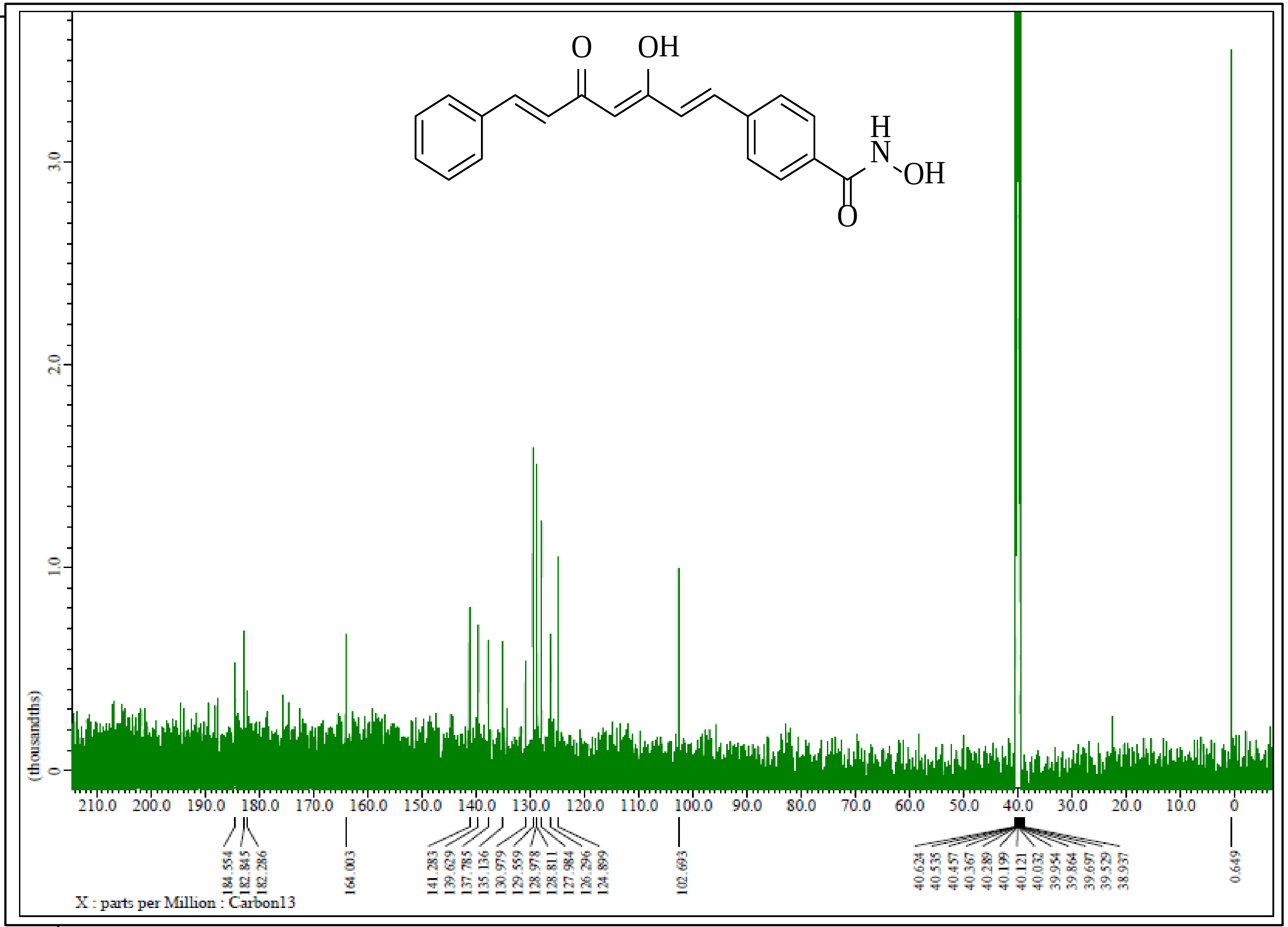

Figure S18. ^{13}C-NMR spectra of 9a / VS-186C

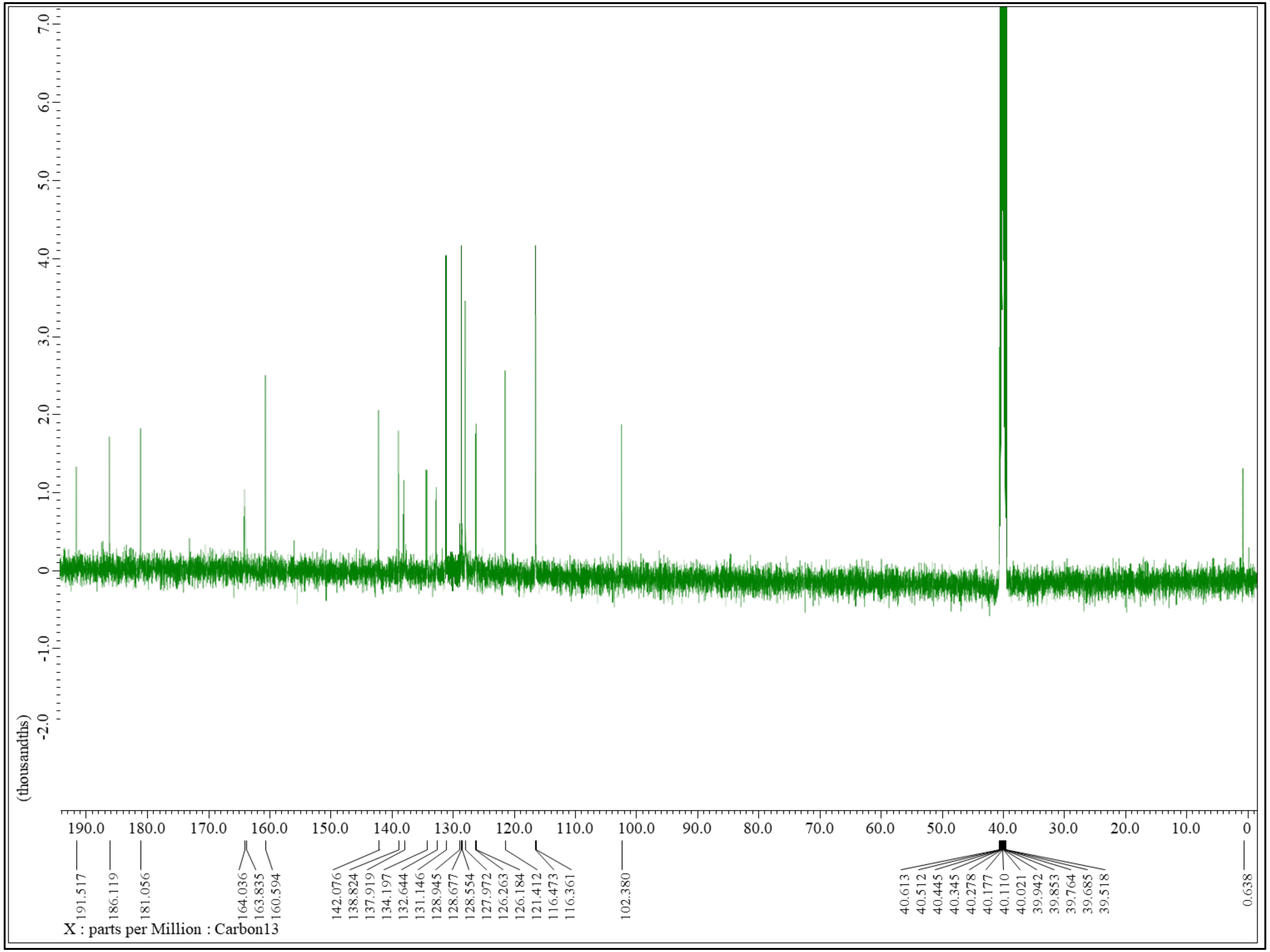

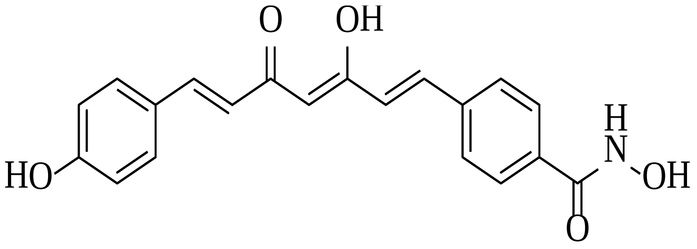

Figure S19. ^{13}C-NMR spectra of 9b / VS-186B

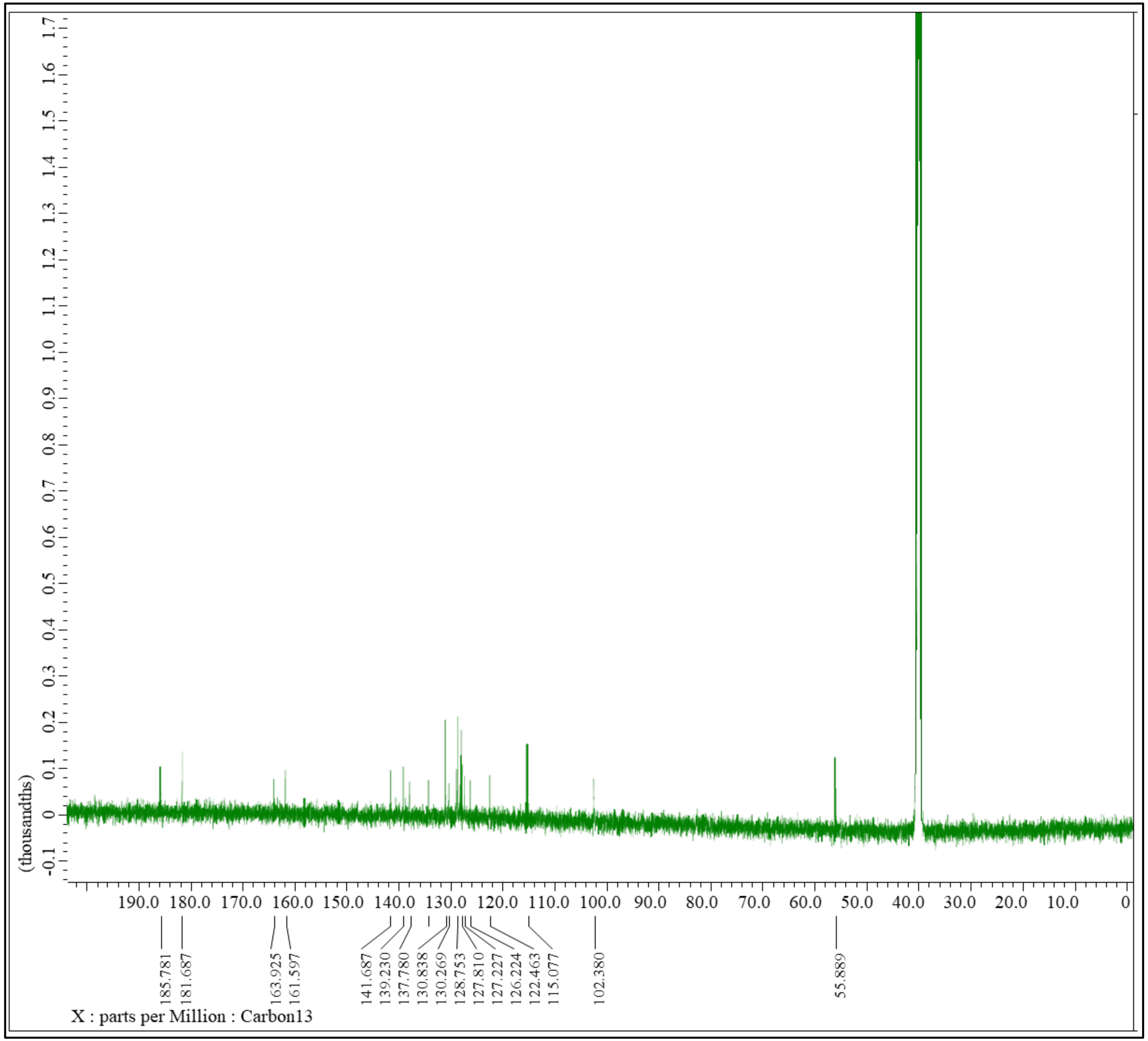

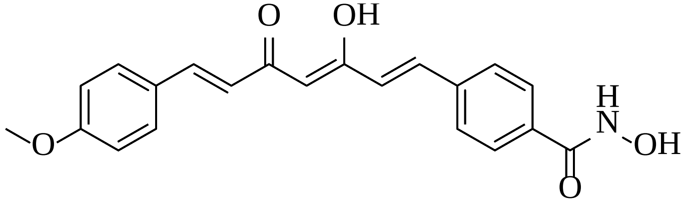

Figure S20. ^{13}C-NMR spectra of 9c / VS-183A

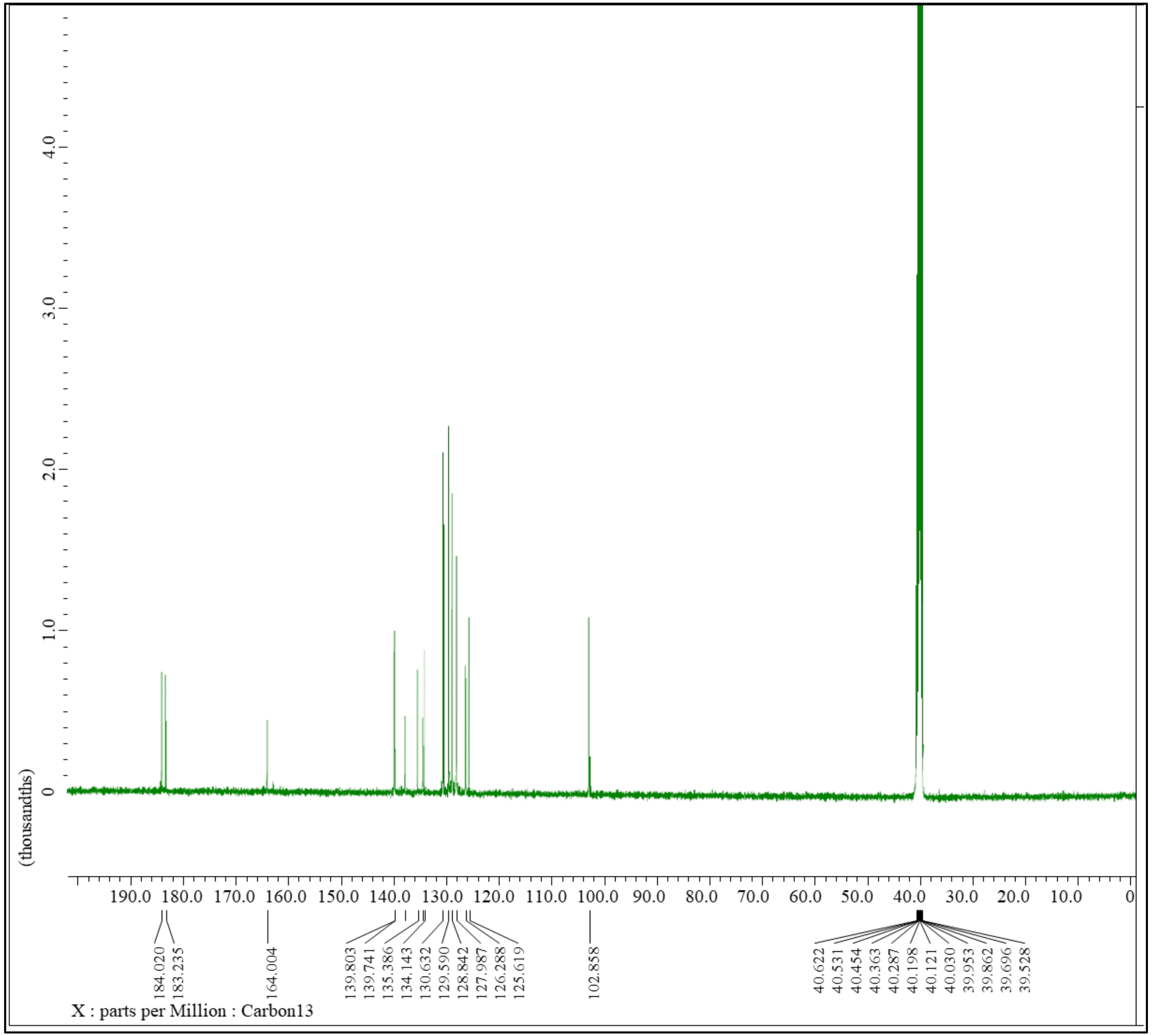

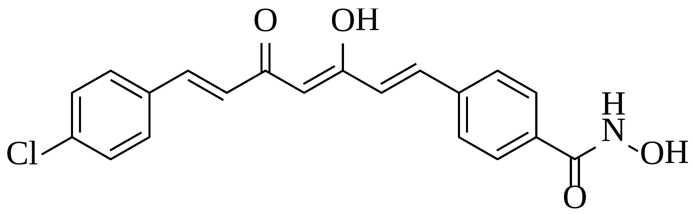

Figure S21. **^{13}C-NMR spectra of 9d / VS-183D**

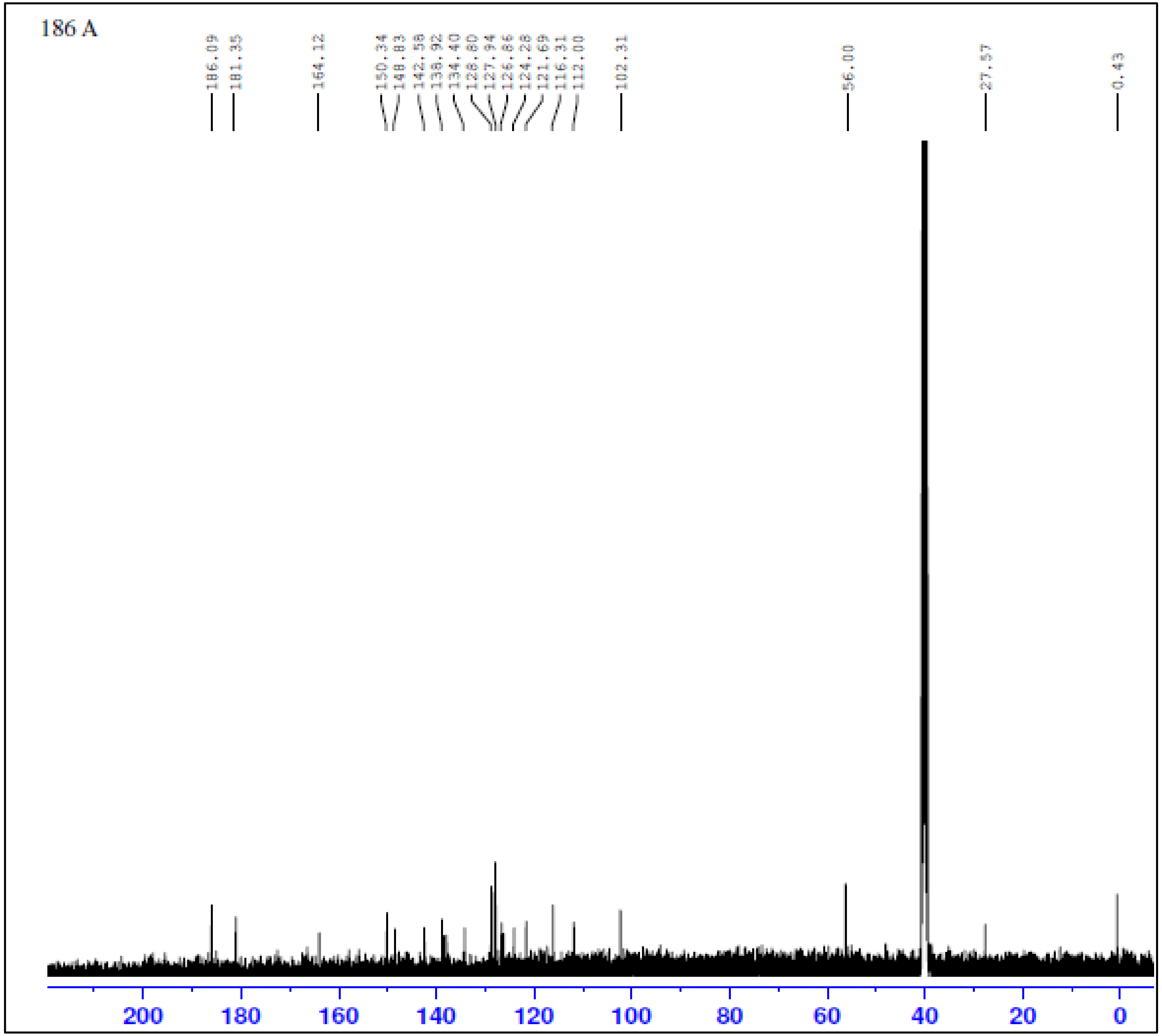

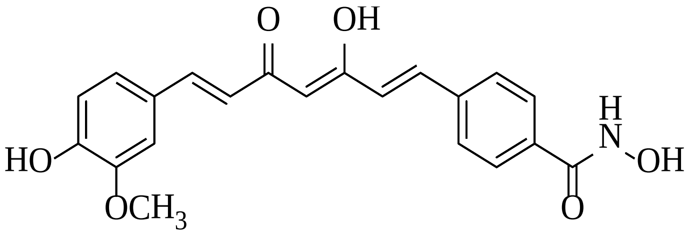

Figure S22. ^{13}C-NMR spectra of 9e / VS-186A

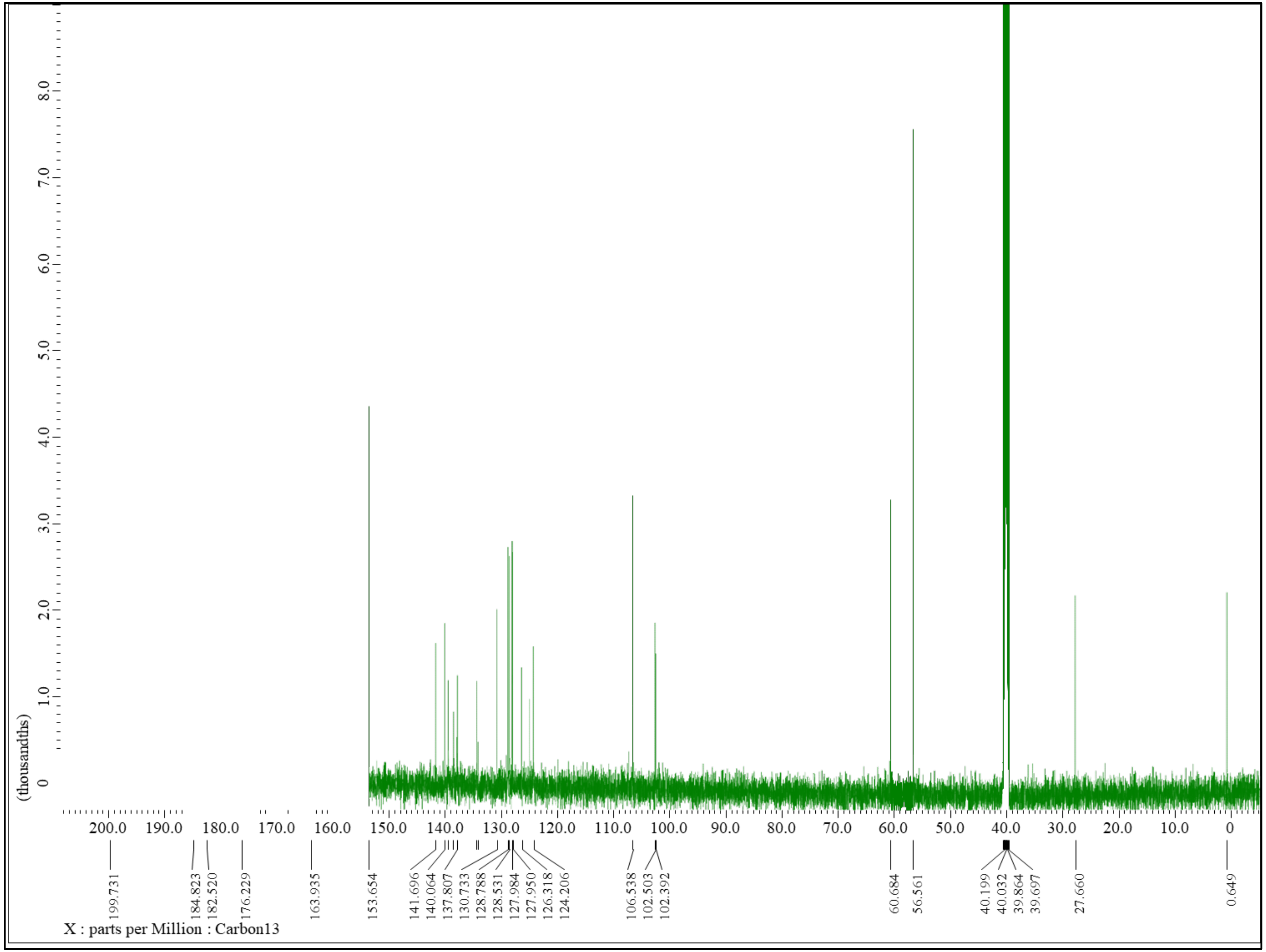

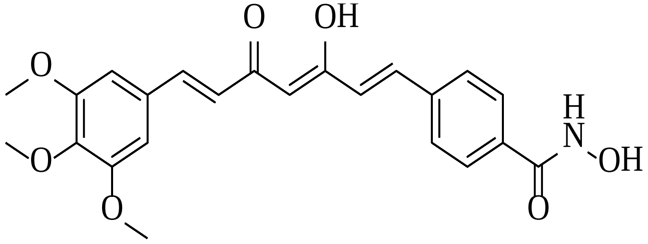

Figure S23. ^{13}C-NMR spectra of 9f / VS-186E

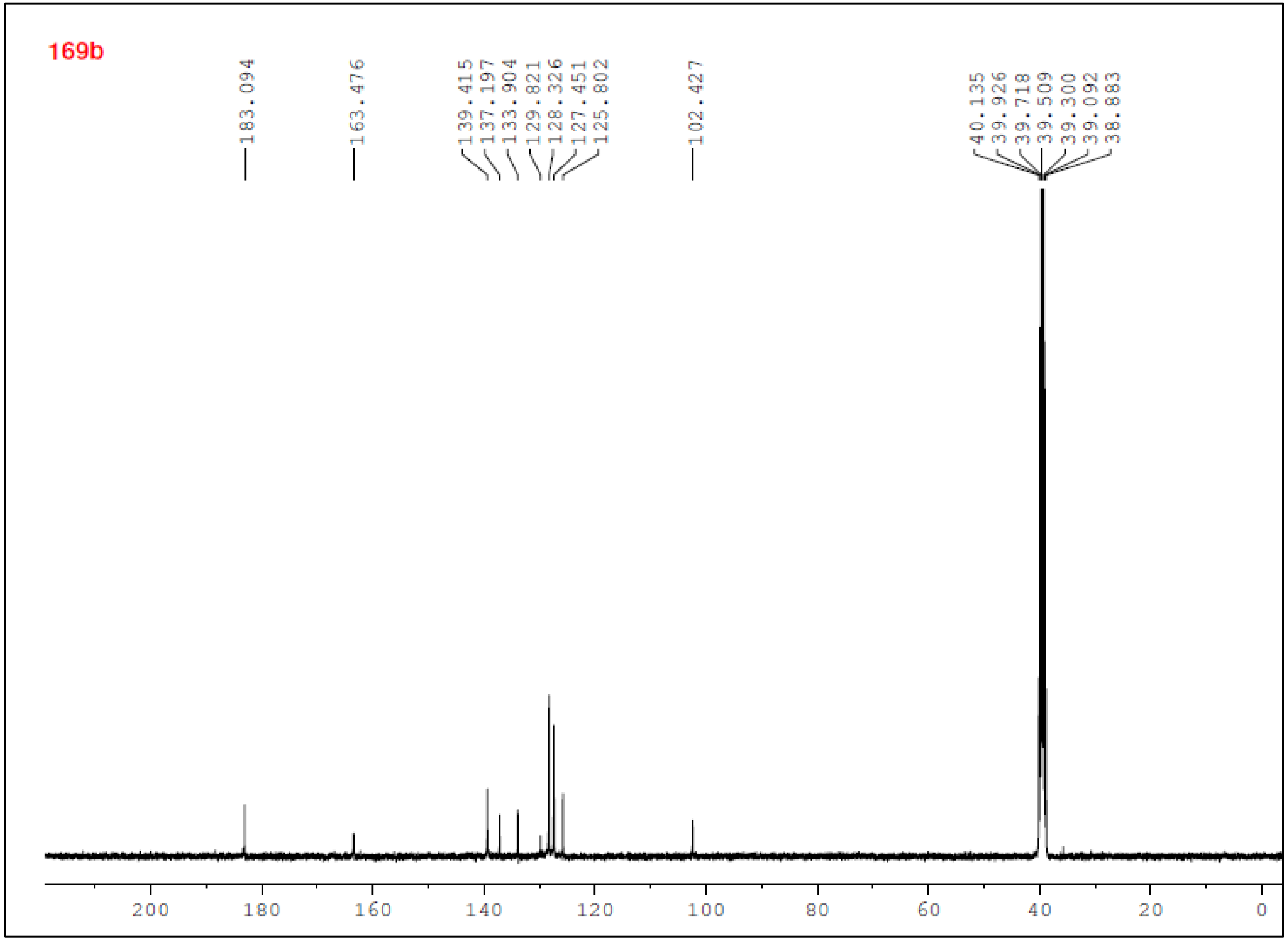

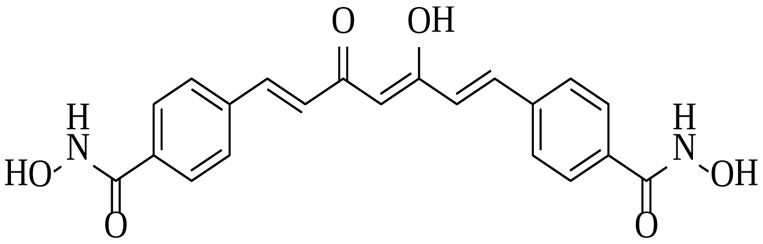

Figure S24. ^{13}**C-NMR spectra of 9g / VS-169B**

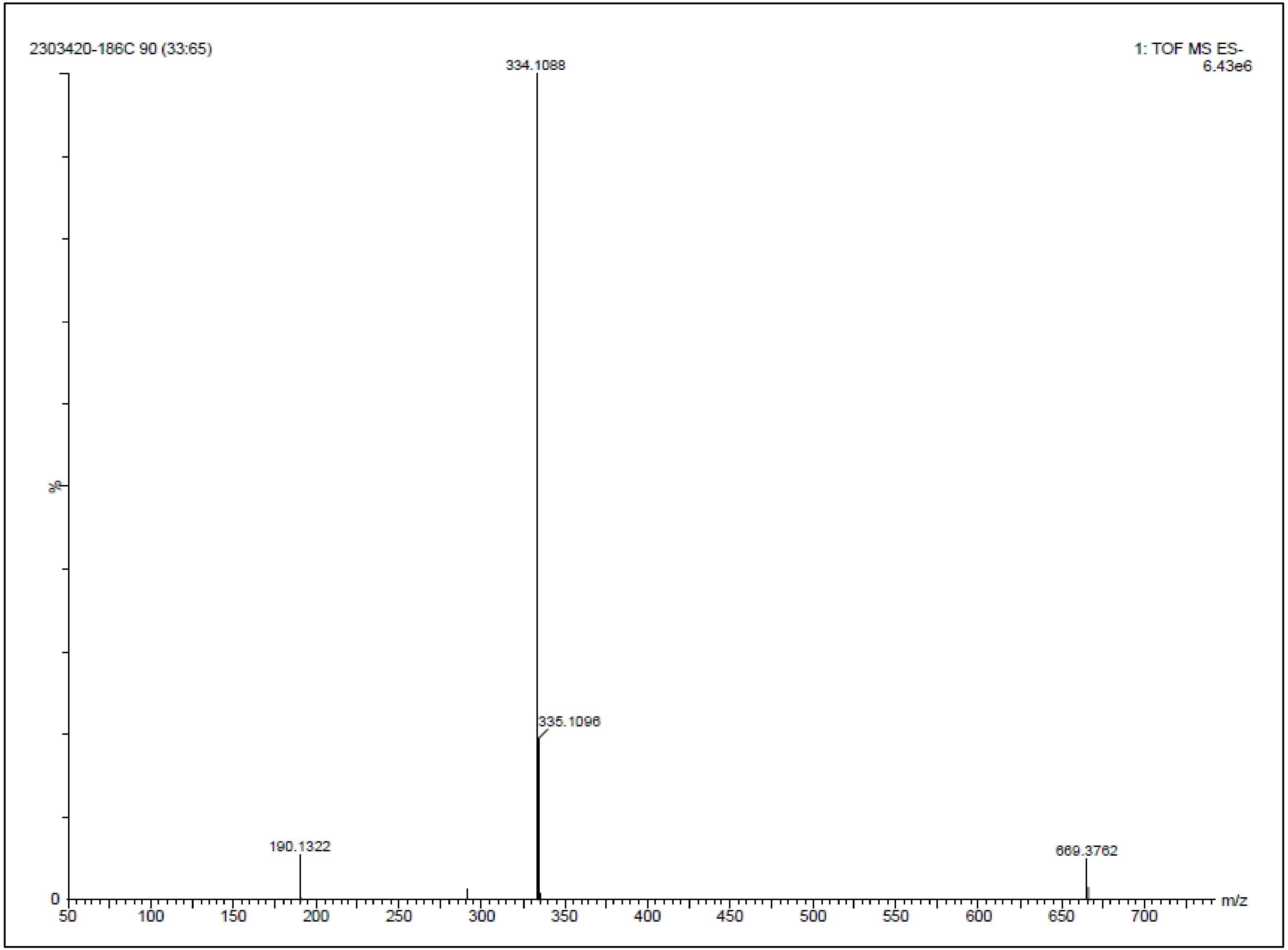

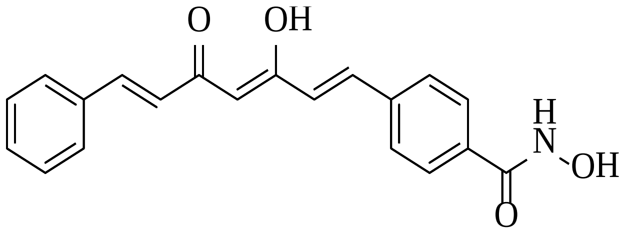

Exact Mass: 335.1158

Figure S25. HRMS spectra of 9a / VS-186C

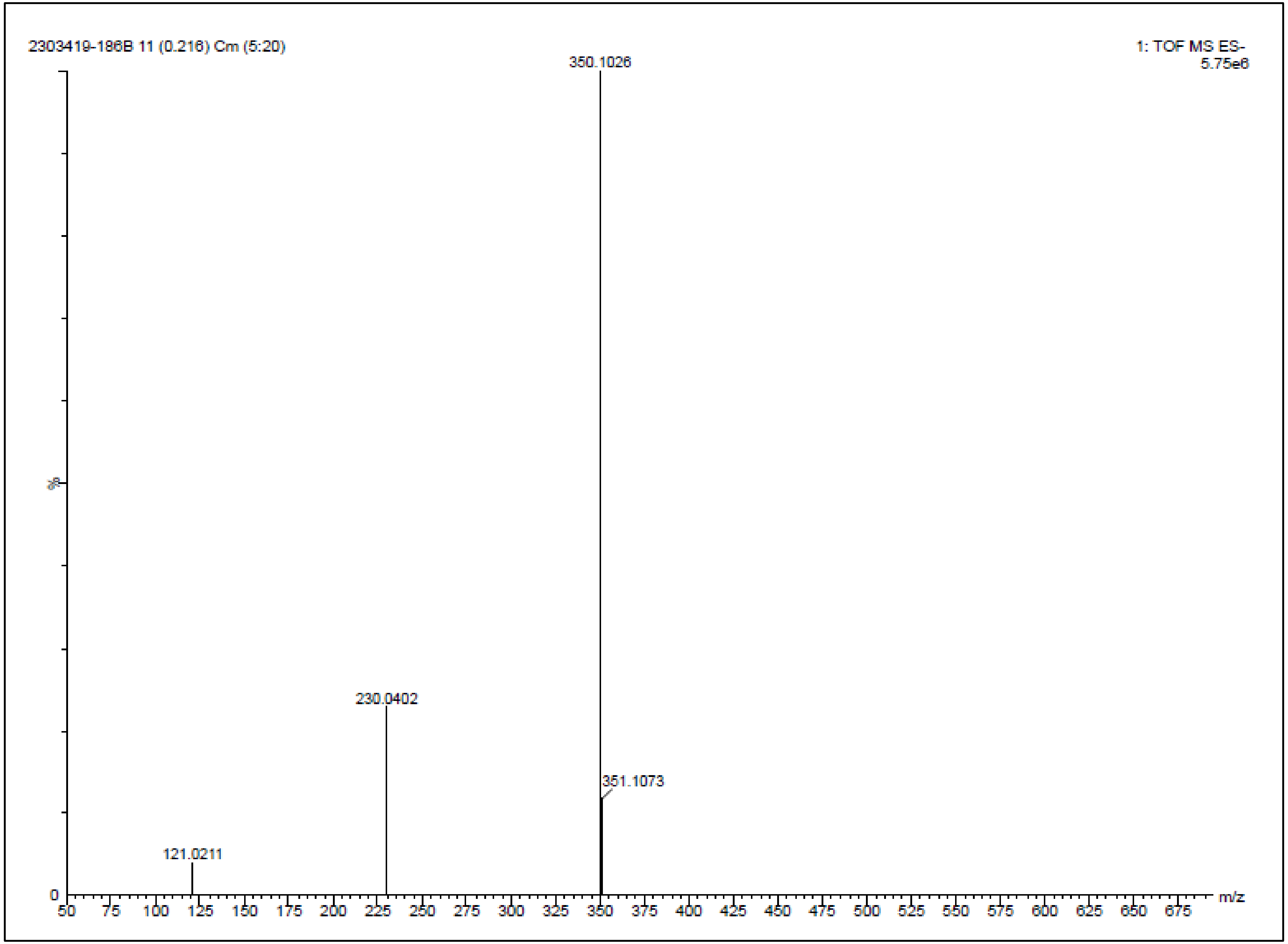

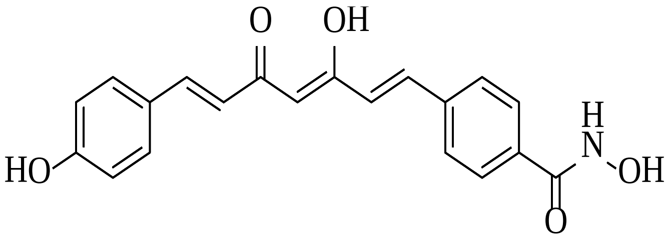

Exact Mass: 351.1107

Figure S26. HRMS spectra of 9b / VS-186B

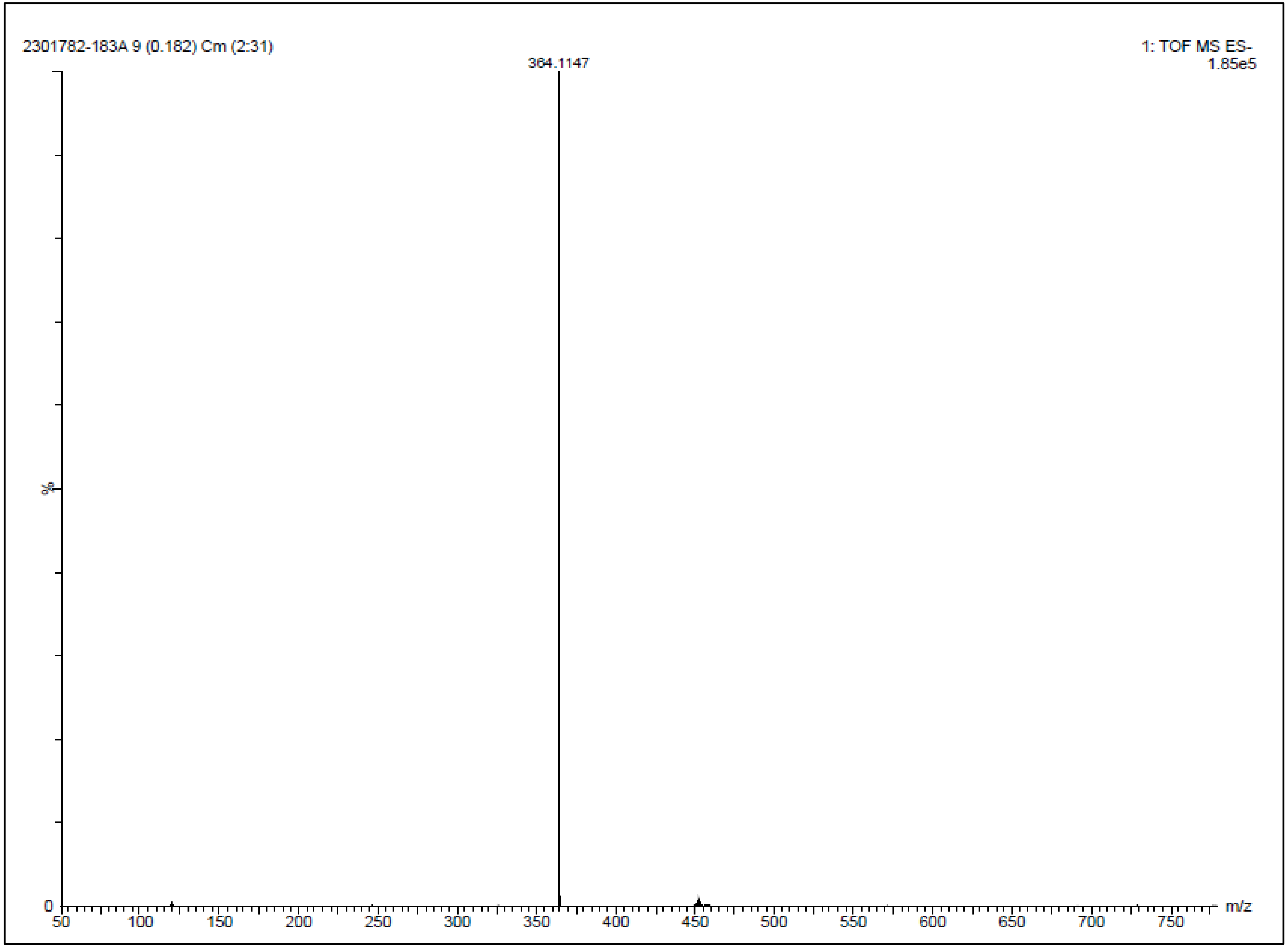

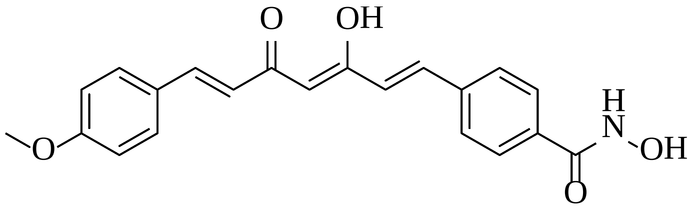

Exact Mass: 365.1263

Figure S27. HRMS spectra of 9c / VS-183A

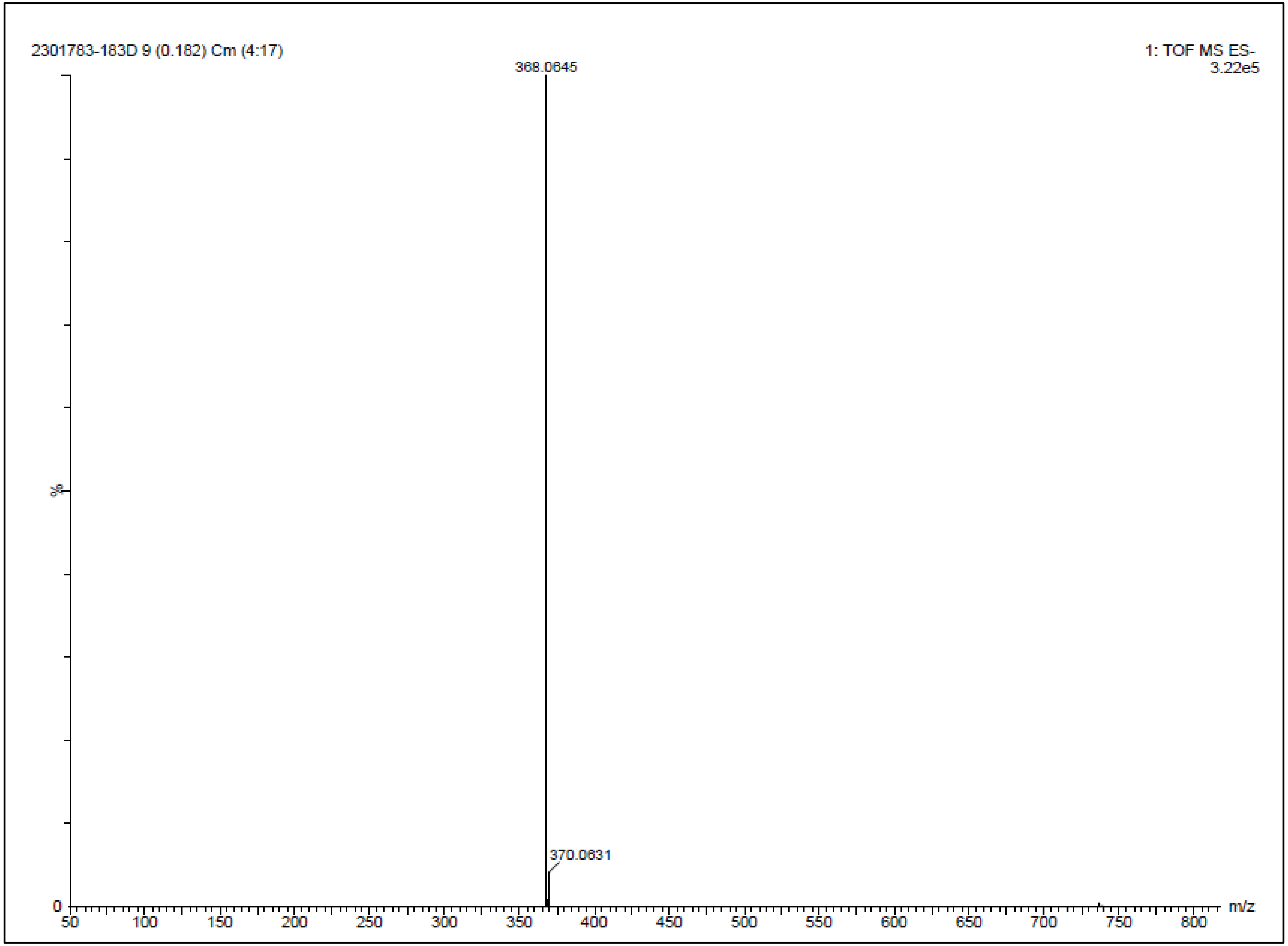

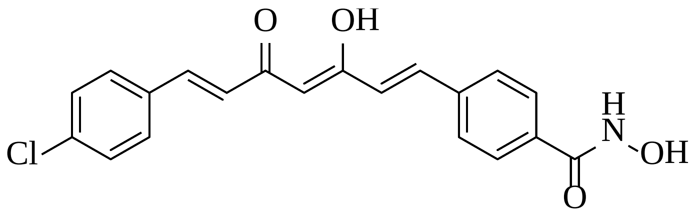

Exact Mass: 369.0768

Figure S28. HRMS spectra of 9d / VS-183D

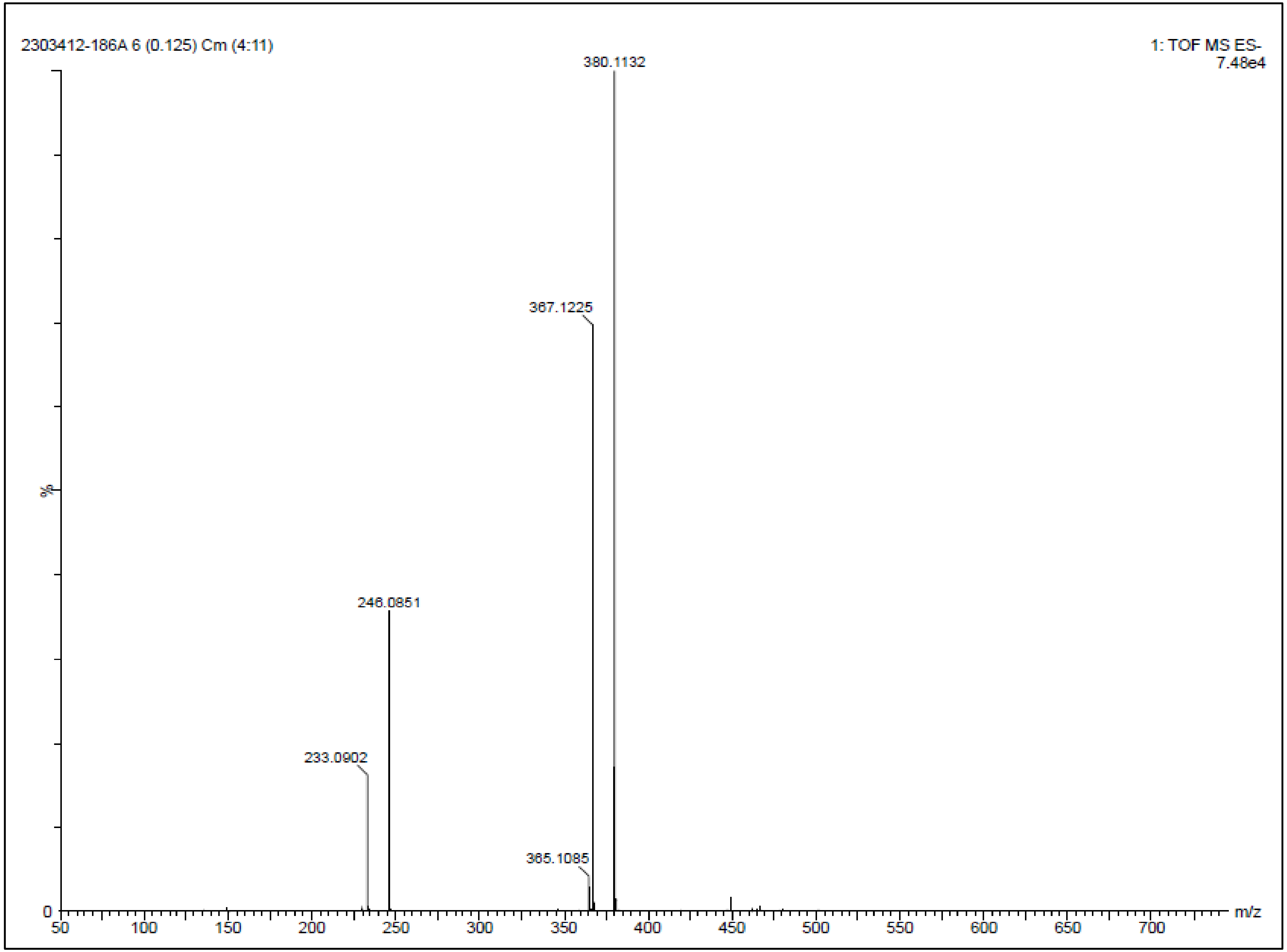

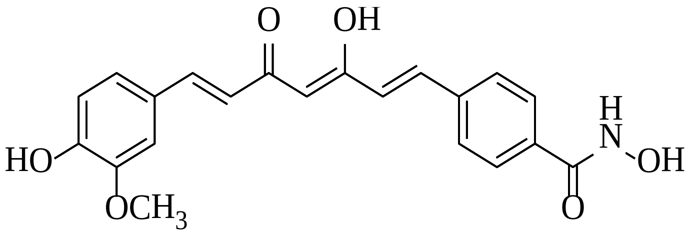

Exact Mass: 381.1212

Figure S29. HRMS spectra of 9e / VS-186A

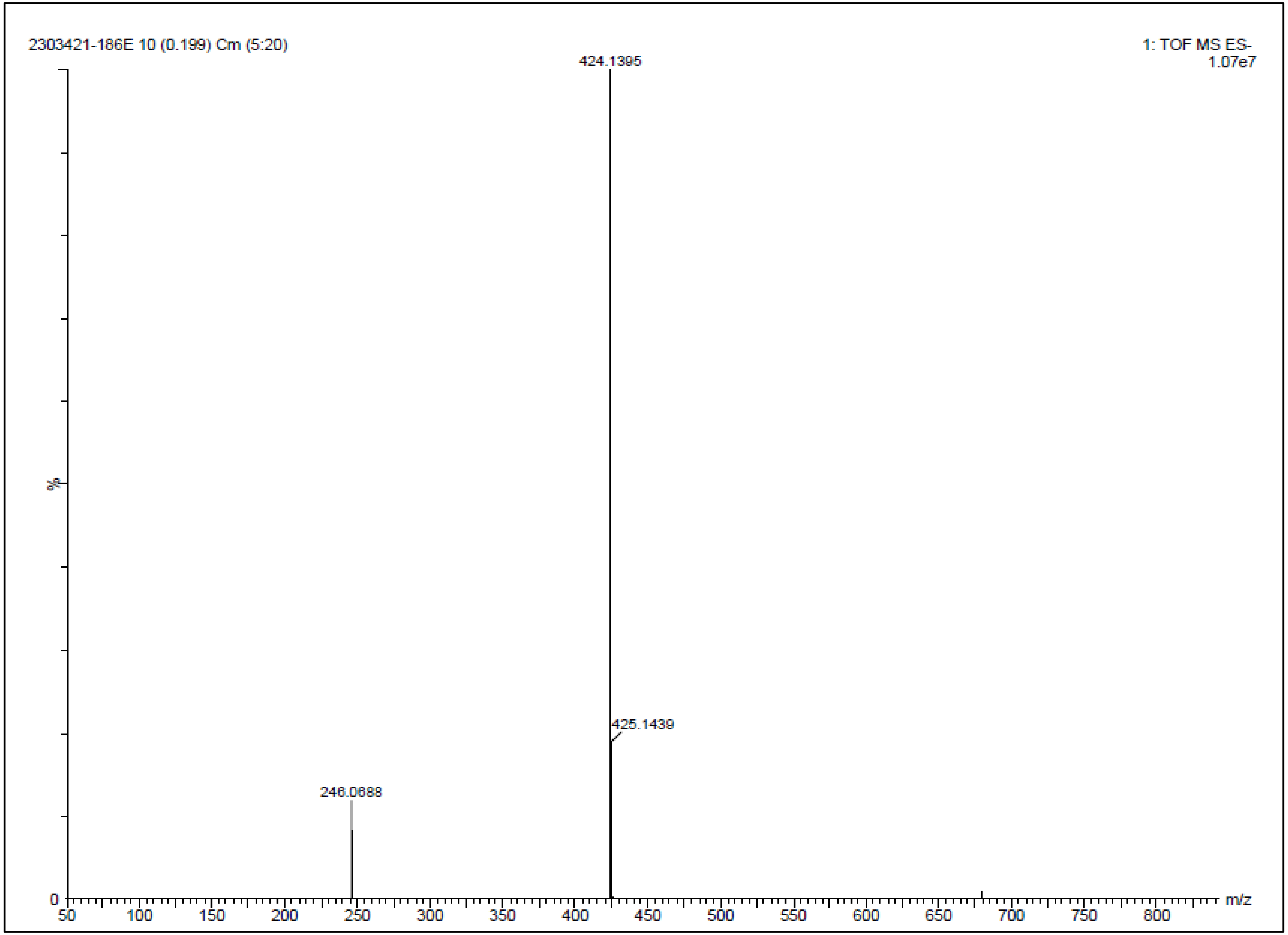

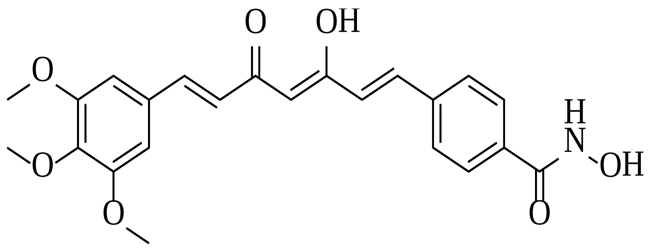

Exact Mass: 425.1475

Figure S30. HRMS spectra of 9f / VS-186E

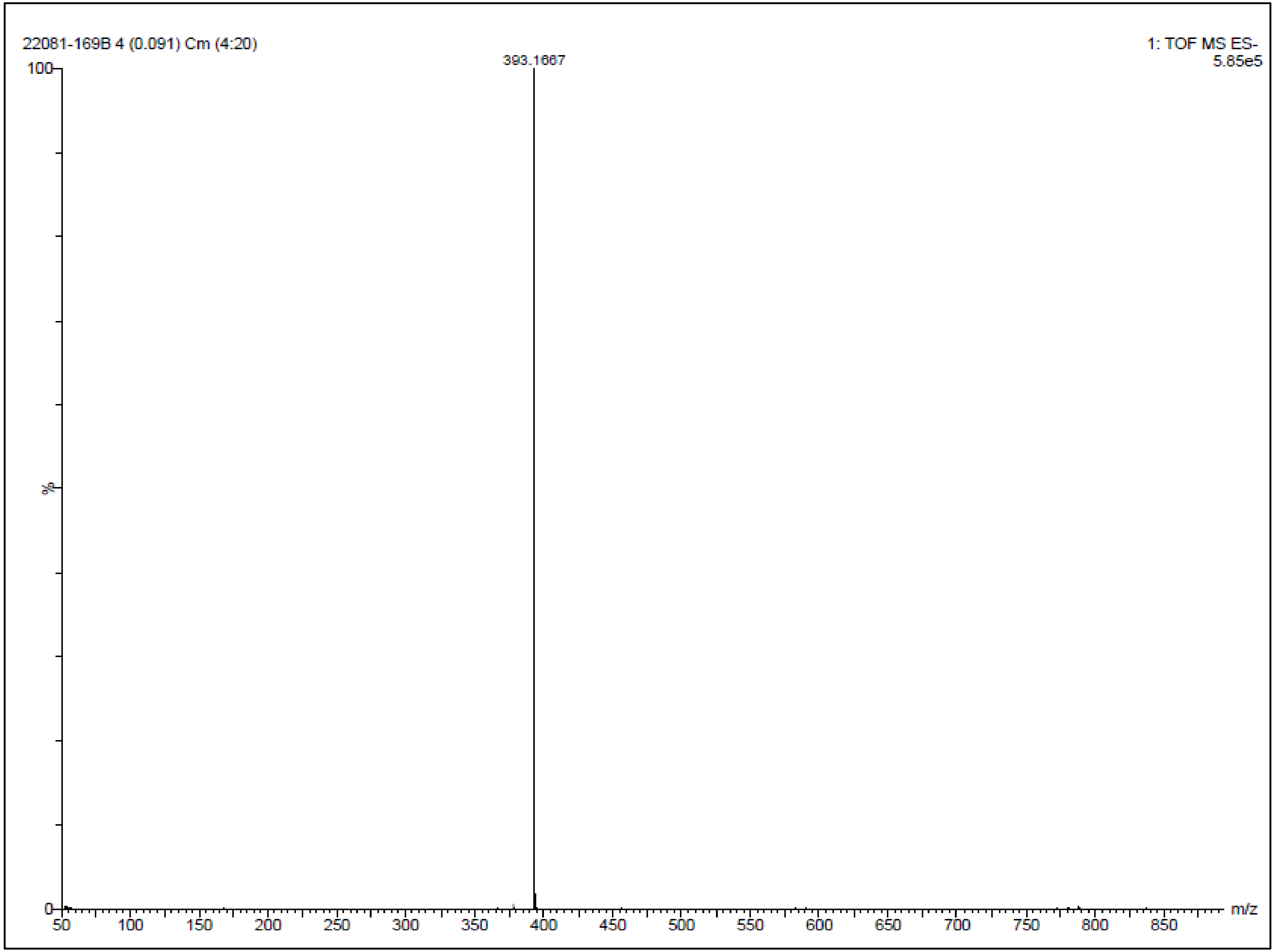

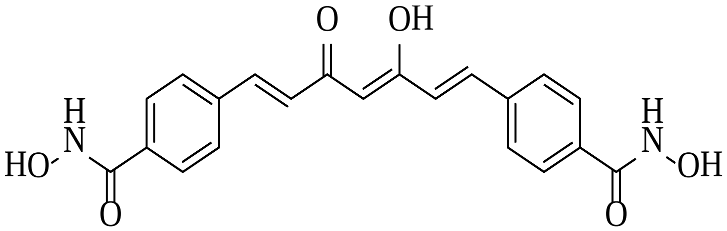

Exact Mass: 394.1165

Figure S31. HRMS spectra of 9g / VS-169B

Figure S32. Annexin V/PI flow cytometry plots

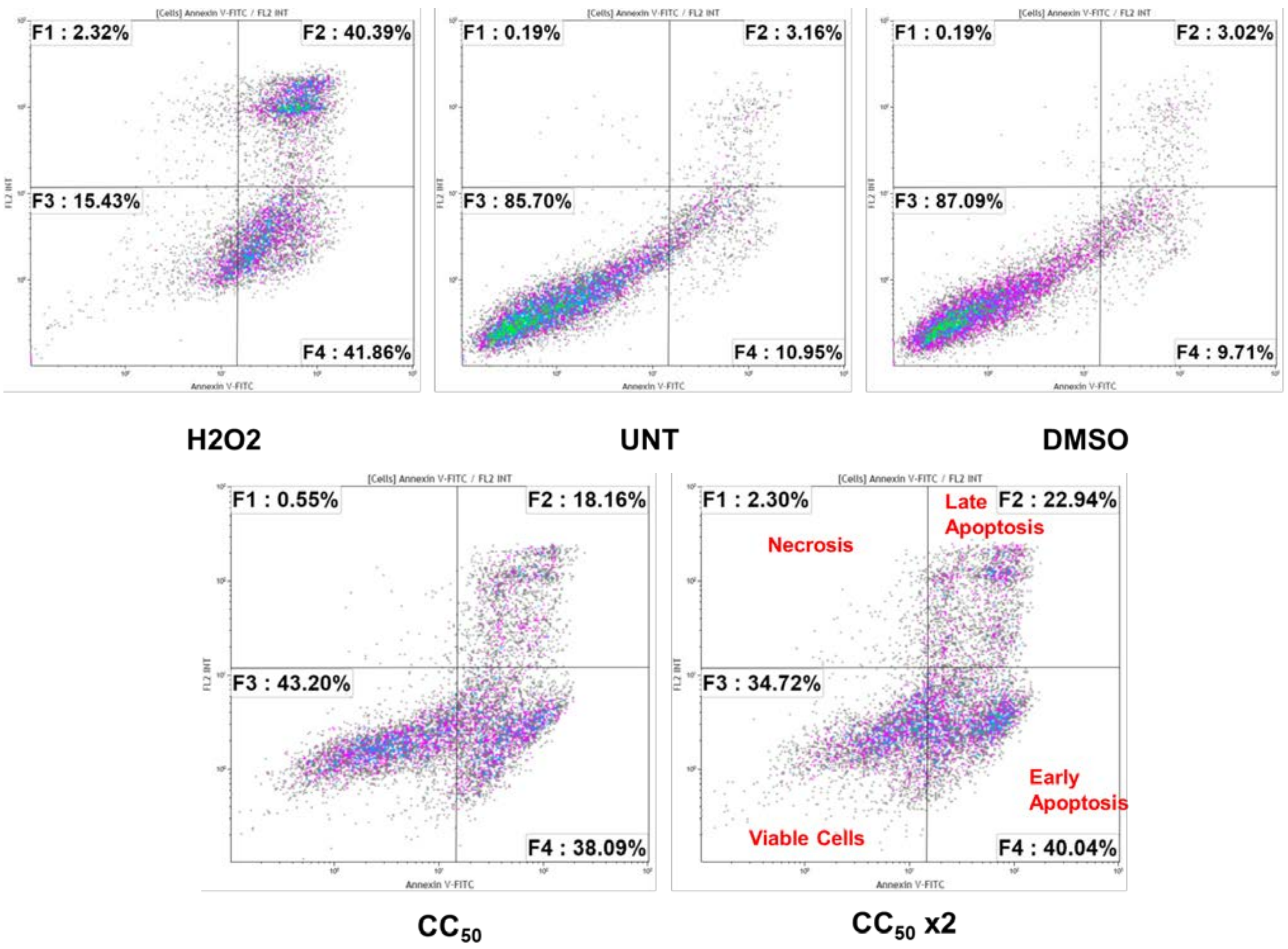

Supplementary Figure S32. Annexin V/PI flow cytometry plots for Jurkat cells treated with VS-186B and controls. Plots show populations of viable cells, early apoptotic cells, late apoptotic cells, and necrotic cells. Quantified results from these experiments are presented in Figure 4 of the main manuscript.

Figure S33. ROS flow cytometry plots

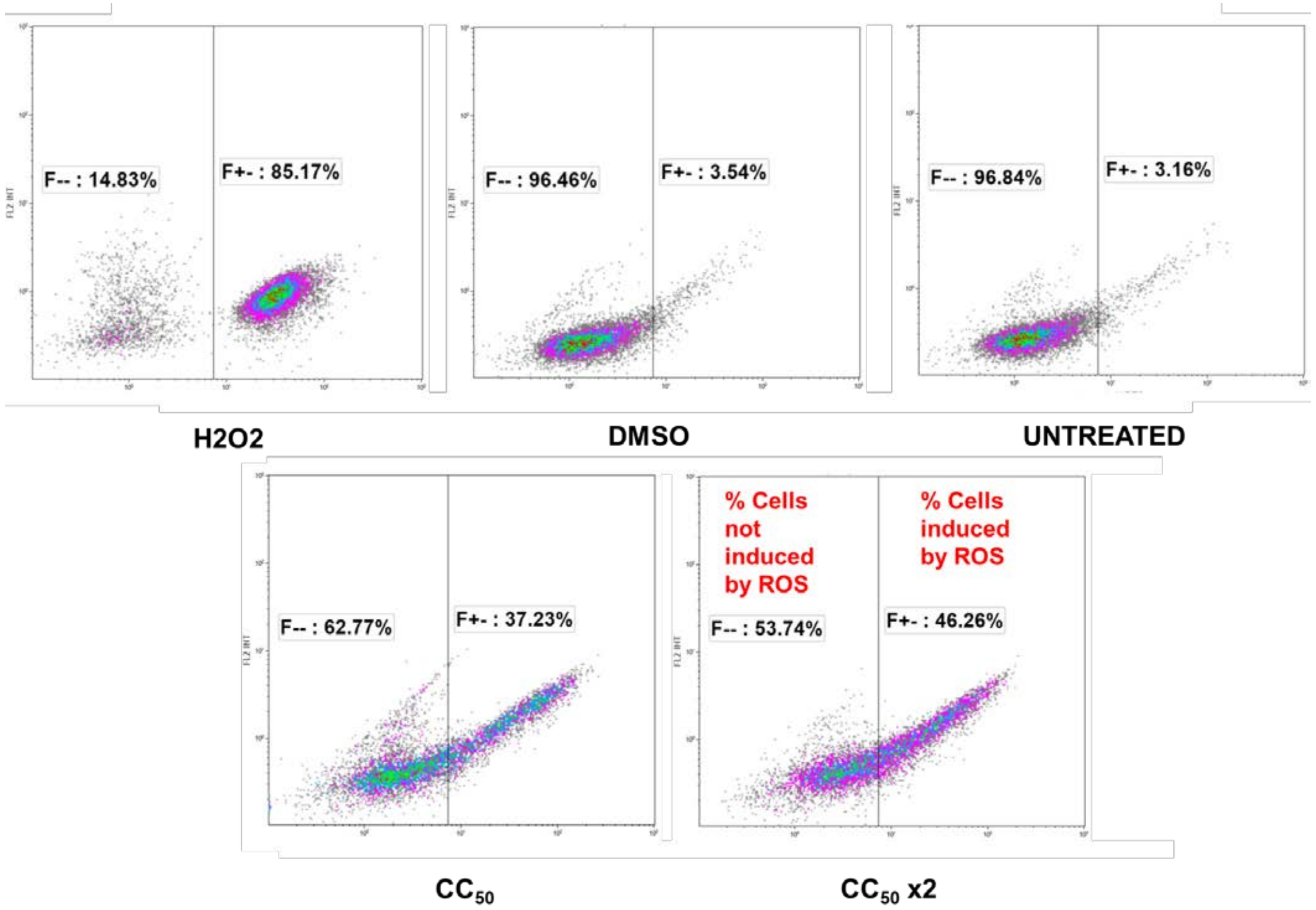

Supplementary Figure S33. ROS flow cytometry plots for Jurkat cells treated with VS-186B and controls. Plots show populations of cells with and without ROS induction. Quantified results from these experiments are presented in Figure 4 of the main manuscript.

Figure S34. JC-1 mitochondrial membrane potential flow cytometry plots

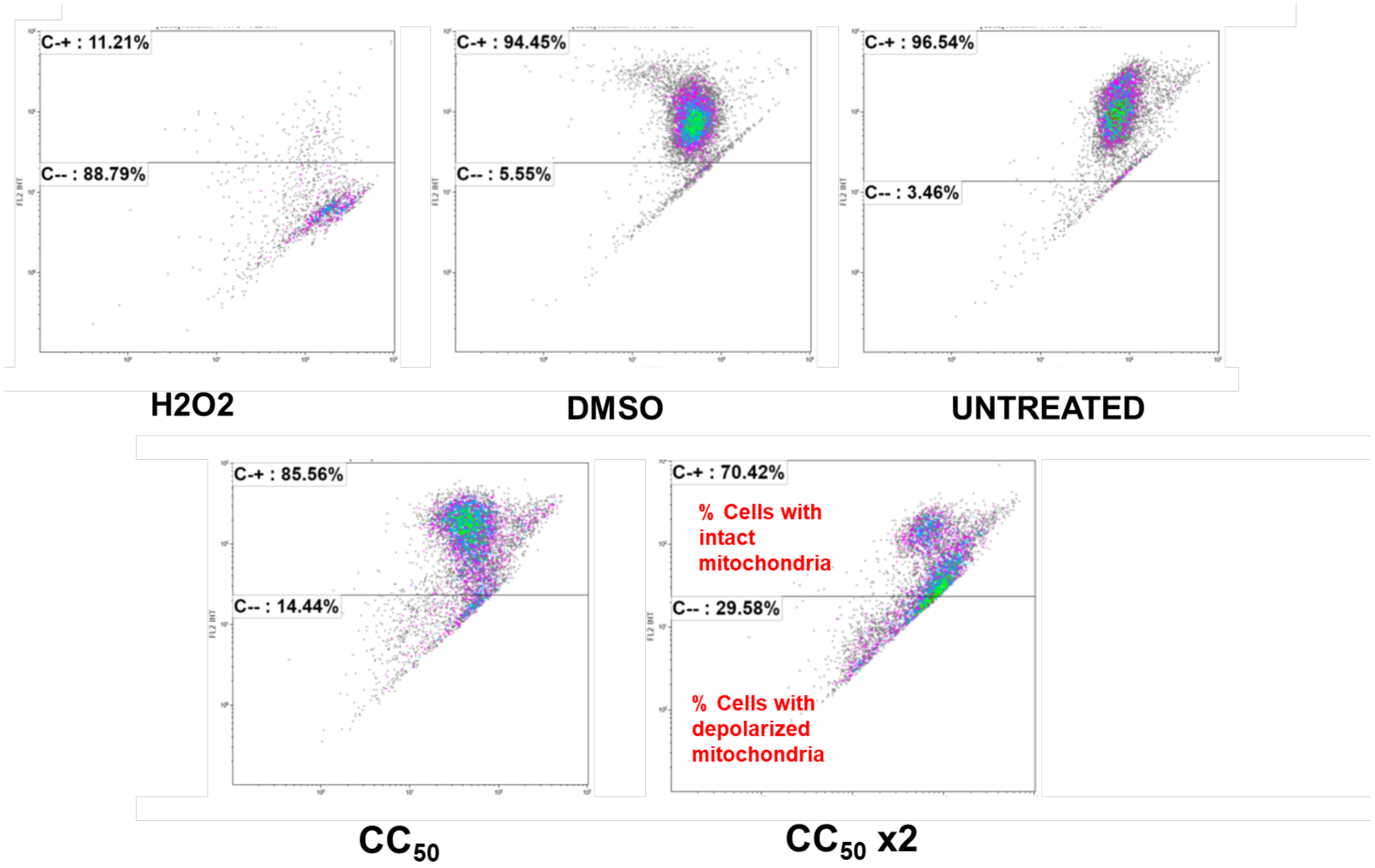

Supplementary Figure S34. JC-1 flow cytometry plots for Jurkat cells treated with VS-186B and controls. Plots show populations of cells with and without depolarized mitochondria. Quantified results from these experiments are presented in Figure 4 of the main manuscript.

Figure S35. Caspase-3/7 flow cytometry plots

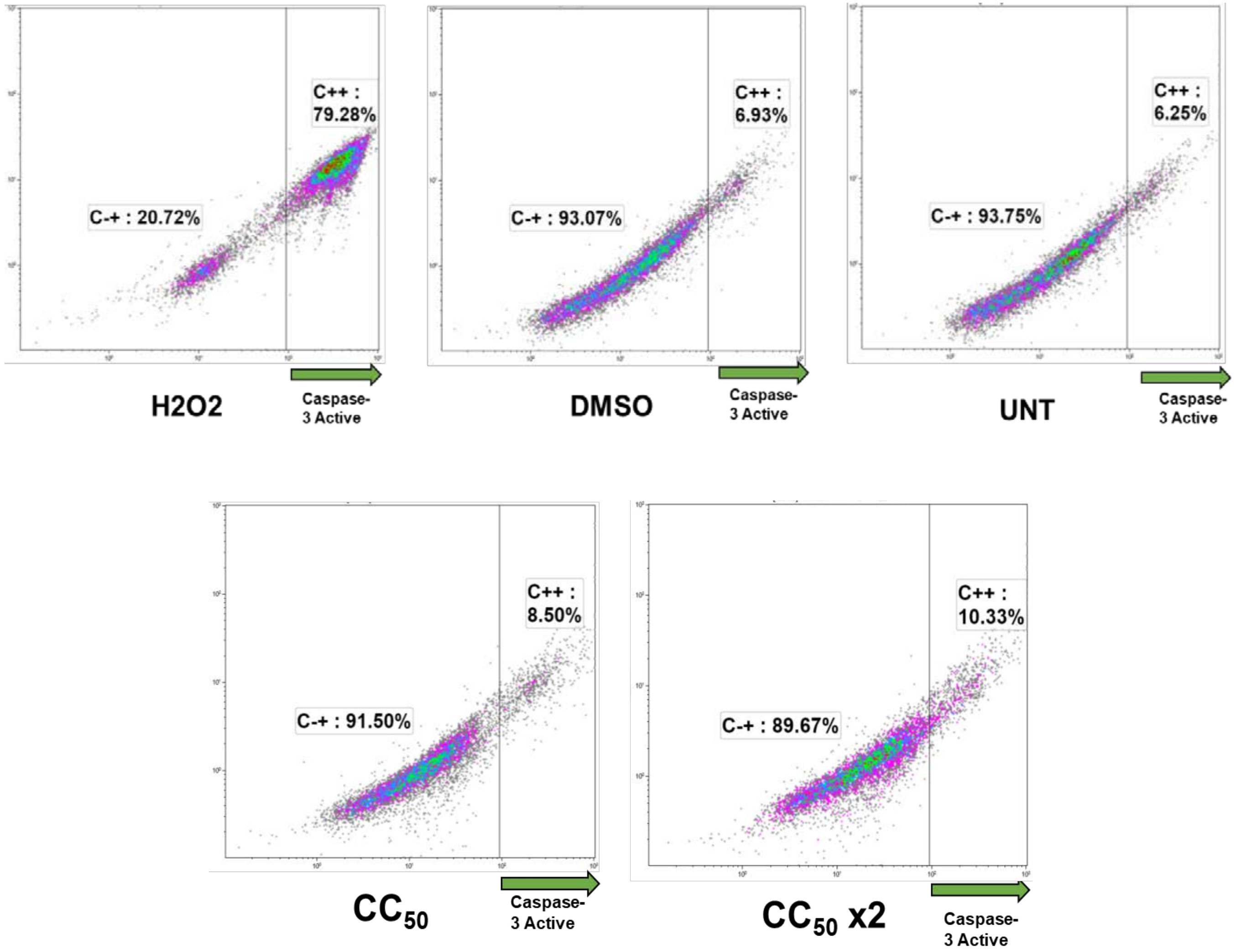

Supplementary Figure S35. Caspase-3/7 flow cytometry plots for Jurkat cells treated with VS-186B and controls. Plots show populations of cells with and without caspase activation. Quantified results from these experiments are presented in Figure 4 of the main manuscript.

Figure S36. Cell cycle flow cytometry plots

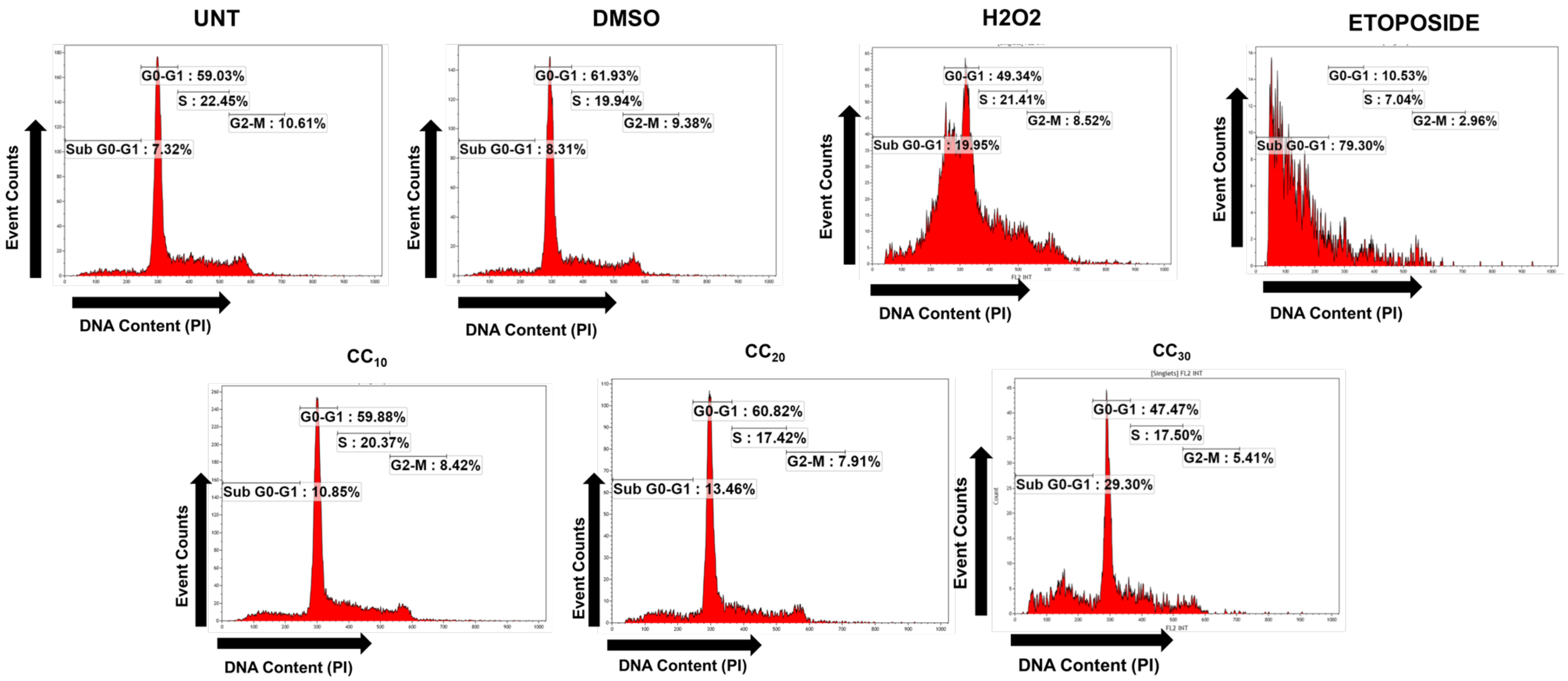

Supplementary Figure S36. Cell cycle flow cytometry plots for Jurkat cells treated with VS-186B and controls, indicating populations in G_0/G_1, S, and G_2/M phases, as well as sub-G_0/G_1 populations representing apoptosis. Quantified results from these experiments are presented in Figure 5 of the main manuscript.

Table S3. CC_{50} values of all novel HDAC inhibitors in multiple cell lines

CC_{50} (μM) and Standard Deviation (SD) in Cancer Cell Lines at 24 h		
**Cell Line**	**Cell Type**	**VS-186B**
**Jurkat**	T-Lymphocyte	3.63 ± 0.63
**MDA-MB-231**	TNBC	54.32 ± 4.95

CC_{50} (μM) and Standard Deviation (SD) in Hematological Cancer Cell Lines at 48 h								
**Cell Line**	**Cell Type**	**VS-169B**	**VS-183A**	**VS-183D**	**VS-186A**	**VS-186B**	**VS-186C**	**VS-186E**
**Jurkat**	T-Lymphocyte	18.59 ± 1.58	ND	ND	5.52 ± 0.13	4.86 ± 0.15	ND	ND
**CEM**	T-Lymphoblast	14.39 ± 1.8	ND	ND	15.97 ± 1.35	3.18 ± 0.25	ND	ND
**HL-60**	Leukemia	14.29 ± 2.18	4.96 ± 0.32	4.92 ± 0.52	8.23 ± 0.94	4.91 ± 0.22	9.30 ± 0.17	12.72 ± 1.56
**NALM6**	B-Cell Precursor Leukemia	25.82 ± 1.94	5.37 ± 0.48	20.32 ± 1.8	6.65 ±0.50	5.00 ± 0.70	26.33 ± 1.2	14.00 ± 2.21
**KMS-11**	Multiple Myeloma	38.80 ± 4.69	ND	>40	21.26 ±4.88	7.31 ± 1.08	32.58 ± 0.35	29.00 ± 2.16
**Ramos**	B-Lymphocyte	25.38 ± 0.67	6.14 ± 0.63	43.14 ± 4.98	8.62 ± 1.09	4.17 ± 1.35	16.29 ± 1.3	21.53 ± 2.35

CC_{50} (μM) and Standard Deviation (SD) in Cell Lines at 72 h								
**Cell Line**	**Cell Type**	**VS-169B**	**VS-183A**	**VS-183D**	**VS-186A**	**VS-186B**	**VS-186C**	**VS-186E**
**Jurkat**	T-Lymphocyte	6.75 ± 0.90	6.26 ± 0.47	3.99 ± 0.80	2.99 ± 0.98	2.91 ± 0.50	12.09 ± 1.26	9.09 ± 0.08
**CEM**	T-Lymphoblast	3.58 ± 1.43	5.49 ± 0.39	4.55 ± 0.73	4.25 ± 0.33	4.55 ± 0.73	7.84 ± 0.25	7.74 ± 0.37
**MM.1S**	Multiple Myeloma	39.98 ± 1.51	6.43 ± 0.18	8.25 ± 0.33	9.12 ± 1.82	4.71 ± 0.11	13.54 ± 1.70	7.62 ± 1.49
**MDA-MB-231**	TNBC	18.88 ± 0.64	28.21 ± 1.96	>40	24.36 ± 1.46	6.99 ± 0.15	32.97 ± 1.94	30.72 ± 0.50
**T-47D**	Breast Cancer (ER+)	ND	31.59 ± 1.60	>40	ND	5.00 ± 0.70	>40	>40
**MCF-7**	Breast Cancer (ER+)	ND	32.51 ± 2.59	>40	34.63 ± 2.19	3.18 ± 0.25	>40	>40
**HEP G2**	Liver Cancer	22.67 ± 2.80	42.35 ± 5.20	28.74 ± 4.84	29.36 ± 1.11	18.68 ± 1.43	47.44 ± 3.04	49.94 ± 2.67
**MCF 10A**	Breast epithelial	18.71 ± 1.90	19.29 ± 0.35	29.31 ± 4.61	23.85 ± 2.03	4.37 ± 0.37	32.34 ± 0.86	31.38 ± 0.83
**Hs27**	Foreskin fibroblast	>40	ND	ND	47.07 ± 1.58	46.82 ± 1.26	ND	ND

Supplementary Table S3. CC_{50} values and standard deviations (SD) of all novel HDAC inhibitors across cancerous and non-cancerous cell lines, at 48 and 72 hours. Jurkat's 24 hour CC_{50} was used for flow cytometry assays and MDA-MB-231's 24 hour CC_{50} was used for RNA sequencing analysis. ND indicates that a CC_{50} value was not determined.

Figure S37. CMap Heatmap

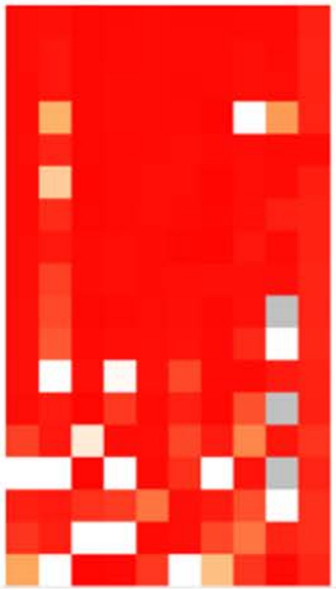

#	type	name	description
1		ISOX	HDAC inhibitor
2		vorinostat	HDAC inhibitor, cell cycle inhibitor
3		trichostatin-a	HDAC inhibitor, CDK expression enhancer, ID1 expression inhibitor
4		scriptaid	HDAC inhibitor
5		HDAC inhibitor	
6		THM-I-94	HDAC inhibitor, apoptosis stimulant, cell cycle inhibitor
7		trichostatin-a	HDAC inhibitor, CDK expression enhancer, ID1 expression inhibitor
8		belinostat	HDAC inhibitor, cell cycle inhibitor
9		HC-toxin	HDAC inhibitor
10		NCH-51	HDAC inhibitor
11		dacinostat	HDAC inhibitor
12		pyroxamide	HDAC inhibitor, cell cycle inhibitor
13		WT-171	HDAC inhibitor
14		apicidin	HDAC inhibitor
15		givinostat	HDAC inhibitor, interleukin receptor antagonist, interleukin synthesis inhibitor, tumor necrosis factor receptor antagonist
16		BI-2536	PLK inhibitor, apoptosis stimulant, cell cycle inhibitor, protein kinase inhibitor
17		panobinostat	HDAC inhibitor, apoptosis stimulant, cell cycle inhibitor
18		APHA-compound-8	HDAC inhibitor

Supplementary Figure S37. Data are based on RNA sequencing of VS-186B–treated MDA-MB-231 cells at 6 hours. Connectivity Map (CMap) analysis revealed that the transcriptional profile of VS-186B closely aligns with HDAC inhibitors.

Figure S38 BOILED-Egg pharmacokinetic analysis

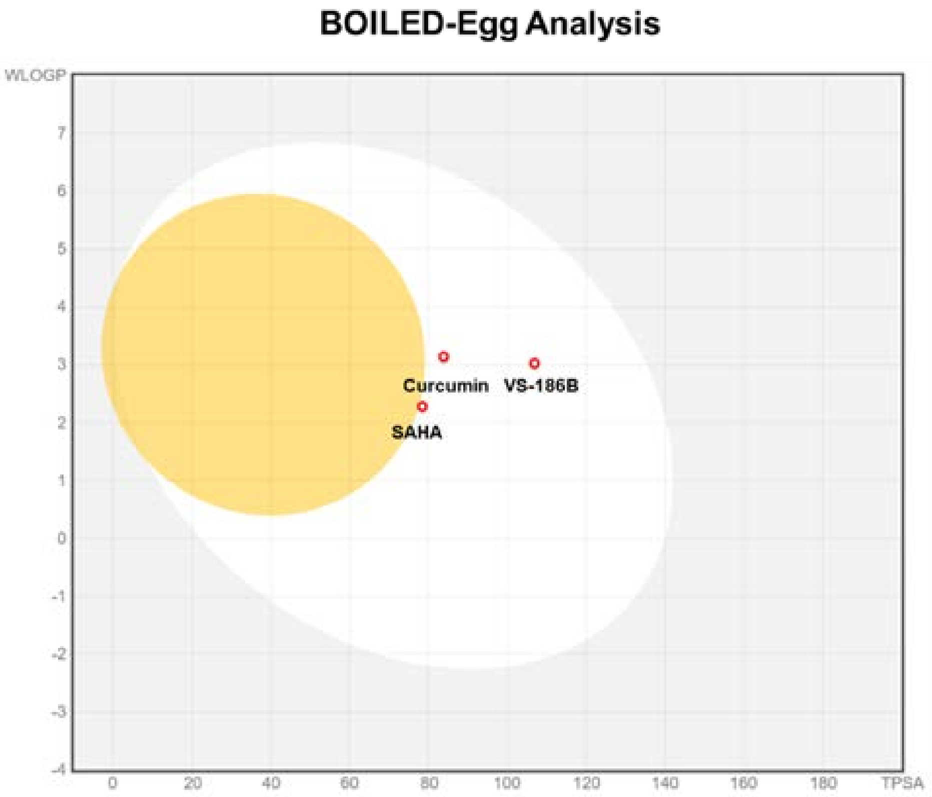

Supplementary Figure S38 BOILED-Egg Analysis comparing VS-186B, SAHA, and Curcumin. BOILED-Egg plot illustrates gastrointestinal absorption (white region), and blood brain barrier permeability (yellow region), along with P-glycoprotein (P-gp) efflux prediction.

Table S4. BOILED-Egg pharmacokinetic analysis

Compound:	TSPA (Å²)	GI Absorption	BBB Permeation	P-gp substrate	Bioavailability Score
VS-186B	106.86	High	No	No	0.56
SAHA	78.43	High	No	No	0.55
Curcumin	83.83	High	No	No	0.55

Supplementary Table S4. The table summarizes key properties including topological polar surface area (TSPA), GI absorption, blood-brain barrier (BBB) permeability, P-gp substrate prediction, and bioavailability score.
